# Histological observations and transcriptome analyses reveal the dynamic changes in the gonads of the blotched snakehead (*Channa maculata*) during sex differentiation and gametogenesis

**DOI:** 10.1186/s13293-024-00643-x

**Published:** 2024-09-07

**Authors:** Xiaotian Zhang, Yuxia Wu, Yang Zhang, Jin Zhang, Pengfei Chu, Kunci Chen, Haiyang Liu, Qing Luo, Shuzhan Fei, Jian Zhao, Mi Ou

**Affiliations:** 1 Key Laboratory of Tropical and Subtropical Fishery Resources Application and Cultivation, Ministry of Agriculture and Rural Affairs, Pearl River Fisheries Research Institute, Chinese Academy of Fishery Sciences, No. 1 Xingyu Road, Xilang, Liwan District, Guangzhou, 510380 Guangdong China; 2 College of Fisheries and Life Science, Shanghai Ocean University, Shanghai, 201306 China; 3 School of Life and Health Sciences, Hunan University of Science and Technology, Xiangtan, 411201 China; 4 College of Animal Science and Technology, Yangzhou University, Yangzhou, 225000 China

**Keywords:** *Channa maculata*, Sexual dimorphism, Sex differentiation, Gametogenesis

## Abstract

**Background:**

Blotched snakehead (*Channa maculata*) displays significant sexual dimorphism, with males exhibiting faster growth rates and larger body sizes compared to females. The cultivation of the all-male population of snakeheads holds substantial economic and ecological value. Nonetheless, the intricate processes governing the development of bipotential gonads into either testis or ovary in *C. maculata* remain inadequately elucidated. Therefore, it is necessary to determine the critical time window of sex differentiation in *C. maculata*, providing a theoretical basis for sex control in production practices.

**Methods:**

The body length and weight of male and female *C. maculata* were measured at different developmental stages to reveal when sexual dimorphism in growth initially appears. Histological observations and spatiotemporal comparative transcriptome analyses were performed on ovaries and testes across various developmental stages to determine the crucial time windows for sex differentiation in each sex and the sex-related genes. Additionally, qPCR and MG2C were utilized to validate and locate sex-related genes, and levels of E_2_ and T were quantified to understand sex steroid synthesis.

**Results:**

Sexual dimorphism in growth became evident starting from 90 dpf. Histological observations revealed that morphological sex differentiation in females and males occurred between 20 and 25 dpf or earlier and 30–35 dpf or earlier, respectively, corresponding to the appearance of the ovarian cavity or efferent duct anlage. Transcriptome analyses revealed divergent gene expression patterns in testes and ovaries after 30 dpf. The periods of 40–60 dpf and 60–90 dpf marked the initiation of molecular sex differentiation in females and males, respectively. Male-biased genes (*Sox11a*, *Dmrt1*, *Amh*, *Amhr2*, *Gsdf*, *Ar*, *Cyp17a2*) likely play crucial roles in male sex differentiation and spermatogenesis, while female-biased genes (*Foxl2*, *Cyp19a1a*, *Bmp15*, *Figla*, *Er*) could be pivotal in ovarian differentiation and development. Numerous biological pathways linked to sex differentiation and gametogenesis were also identified. Additionally, E_2_ and T exhibited sexual dimorphism during sex differentiation and gonadal development. Based on these results, it is hypothesized that in *C. maculata*, the potential male sex differentiation pathway, *Sox11a*–*Dmrt1*–*Sox9b*, activates downstream sex-related genes (*Amh*, *Amhr2*, *Gsdf*, *Ar*, *Cyp17a2*) for testicular development, while the antagonistic pathway, *Foxl2/Cyp19a1a*, activates downstream sex-related genes (*Bmp15*, *Figla*, *Er*) for ovarian development.

**Conclusions:**

This study provides a comprehensive overview of gonadal dynamic changes during sex differentiation and gametogenesis in *C. maculata*, establishing a scientific foundation for sex control in this species.

**Supplementary Information:**

The online version contains supplementary material available at 10.1186/s13293-024-00643-x.

## Plain language summary

Blotched snakehead (*Channa maculata*) exhibits significant sexual dimorphism, as males display faster growth rates and larger body sizes compared to females. The cultivation of the all-male population of snakeheads holds substantial economic and ecological value. However, the mechanisms underlying sex determination and differentiation in *C. maculata* remain insufficiently elucidated. In this study, sexual dimorphism in growth became evident starting from 90 dpf through the measurement of body length and weight of male and female *C. maculata* at different developmental stages. Histological observations indicated that morphological sex differentiation in females and males occurred at 20–25 dpf or earlier and 30–35 dpf or earlier, respectively, corresponding to the appearance of the ovarian cavity or efferent duct anlage. Transcriptome analyses revealed divergent gene expression patterns in male and female gonads after 30 dpf, suggesting that the period preceding 30 dpf might be the critical time window for sex control in *C. maculata.* The periods of 40–60 dpf and 60–90 dpf marked the initiation of molecular sex differentiation in females and males, respectively. Male-biased genes (*Sox11a*, *Dmrt1*, *Amh*, *Amhr2*, *Gsdf*, *Ar*, *Cyp17a2*) likely play crucial roles in testicular differentiation and spermatogenesis, while female-biased genes (*Foxl2*, *Cyp19a1a*, *Bmp15*, *Figla*, *Er*) could be pivotal in ovarian differentiation and oogenesis. Additionally, numerous biological pathways linked to sex differentiation and gametogenesis were identified. Moreover, sexual dimorphism was observed in the levels of E_2_ and T during gonadal differentiation and development. Based on these findings, it is hypothesized that in *C. maculata*, the potential male sex differentiation pathway, *Sox11a*–*Dmrt1*–*Sox9b*, activates downstream sex-related genes (*Amh*, *Amhr2*, *Gsdf*, *Ar*, *Cyp17a2*) for testicular development, while the antagonistic pathway, *Foxl2/Cyp19a1a*, activates downstream sex-related genes (*Bmp15*, *Figla*, *Er*) for ovarian development. This study provides a comprehensive overview of gonadal dynamic changes during sex differentiation and gametogenesis in *C. maculata*, thereby establishing a scientific foundation for sex control in this species.

**Supplementary Information:**

The online version contains supplementary material available at 10.1186/s13293-024-00643-x.

## Highlights

- Sexual dimorphism in growth became apparent starting from 90 dpf.
- The morphological differentiation of ovary and testis occurred at 20–25 dpf or earlier and 30–35 dpf or earlier, respectively, and the period preceding 30 dpf may be the critical time for sex control in *C. maculata.*
- Male-biased genes (*Sox11a*, *Dmrt1*, *Amh*, *Amhr2*, *Gsdf*, *Ar*, *Cyp17a2*) likely play crucial roles in testicular differentiation and spermatogenesis, whereas female-biased genes (*Foxl2*, *Cyp19a1a*, *Bmp15*, *Figla*, *Er*) could be pivotal in ovarian differentiation and oogenesis.
- The fate of undifferentiated primordial gonads in *C. maculata* may be determined by the antagonistic action of the *Sox11a*–*Dmrt1*–*Sox9b* and *Foxl2/Cyp19a1a* pathways*.*

**Supplementary Information:**

The online version contains supplementary material available at 10.1186/s13293-024-00643-x.

## Background

Sex has been a focal point in life sciences [1]. Fish, with their unique and pivotal role in the evolutionary trajectory of vertebrates, encompass nearly all known forms of sex chromosomes [2]. Sexual dimorphism is prevalent among most fish species, particularly in economically important traits such as growth rate and body size. Consequently, the cultivation of monosex population attains heightened significance due to their substantial economic value and ecological advantages [3]. Understanding the mechanism of sex determination and differentiation in fish is crucial for advancing aquaculture practices, not only for improving economically sex-related traits but also for contributing to uncover the evolutionary process of sex chromosomes in vertebrates.

The sex of teleost is determined and differentiated from bipotential gonads by genetic, environmental factors, or both, which is complex and plastic. Sex determination is the biological process by which sex is established. For genetically determined fish, sex is determined at fertilization depending on whether the individual has an X/Y or a Z/W chromosome [4]. The sex-determining gene acts as a master switch to bipotential gonads and initiates the process of sex determination and differentiation [3]. The first sex-determining gene identified in fish was *Dmy/Dmrt1b*^*Y*^, linked to the Y chromosome in Japanese medaka (*Oryzias latipes*) [5]. Subsequently, numerous key sex-determining genes have been unveiled in diverse fish species, such as *Amhr2* in tiger puffer (*Takifugu rubripes*) [6], *Sd*^*Y*^ in rainbow trout (*Oncorhynchus mykiss*) [7], *Gsdf*^*Y*^ in *O. luzonensis* [8], *Amh*^*Y*^ in Patagonian pejerrey (*Odontesthes hatcheri*) [9] and Nile tilapia (*Oreochromis niloticus*) [10], *Sox3*^*Y*^ in *O. dancena* [11], *Dmrt1* in half-smooth tongue sole (*Cynoglossus semilaevis*) [12], *Gdf6*^*Y*^ in turquoise killifish (*Nothobranchius furzeri*) [13], and *Bcar1* in channel catfish (*Ictalurus punctatus*) [14]. These findings indicate the diversity of sex-determining genes in fish, and multiple genes have the potential to traverse the genetic hierarchy to become new sex-determining gene through gene duplication or allele mutation.

Sex differentiation is the process by which the undifferentiated primordial gonads develop into either testes or ovaries after sex has been determined [4]. This process is labile and influenced by genes, hormones, and extrinsic factors, providing opportunities to manipulate sex ratios in fish [3]. Therefore, an accurate understanding of sex differentiation from morphological, cytological, and molecular levels is crucial for sex control in aquaculture. The determination of the precise timing of this process through histology and transcriptome analyses has provided valuable insights for production practices in common carp (*Cyprinus carpio*) [15, 16]. Unlike sex-determining genes, which exhibit limited conservation across vertebrates, the downstream sex-differentiation-related genes within the genetic network are relatively conserved across most vertebrates [4]. The expression profiles of these genes typically display sexual dimorphism during sex differentiation and gonadal development. For example, *Dmrt1*, *Amh*, and *Gsdf* are involved in testicular differentiation and spermatogenesis, whereas *Cyp19a1a*, *Foxl2*, and *Figla* are linked to ovarian differentiation and oogenesis [3]. *Dmrt1*, serving as the key sex-determining gene in birds [17] and *C. semilaevis* [12], initiates the male signaling pathway. Moreover, the sex-determining gene *Dmy/Dmrt1b*^*Y*^ on the Y chromosome of *O. latipes* [5] and *Dmw* on the W chromosome of African clawed frog (*Xenopus laevis*) [18] both derive from the duplication of *Dmrt1*. *Dmrt1* also plays a critical role in testicular differentiation in teleosts. In zebrafish (*Danio rerio*), the absence of *Dmrt1* suppresses *Amh* expression while promoting *Foxl2* expression, leading to defects in testicular development and an increased proportion of female individuals [19]. In *O. niloticus*, the deletion of *Dmrt1* results in reduced *Sox9b* expression, elevated expression of *Foxl2* and *Cyp19a1a*, increased 17β-estradiol levels, and severe testicular degeneration phenotypes, including degenerated spermatogonia or the complete absence of germ cells, as well as significantly increased proliferation of steroidogenic cells [20]. Additionally, over-expression of *Dmrt1* in female tilapia leads to decreased *Cyp19a1a* expression, reduced 17β-estradiol levels, delayed ovarian cavity development, follicular atrophy of varying degrees, and even sex reversal [21].

While *Dmrt1* is crucial for male sex determination and differentiation, *Foxl2* emerges as a key player in female sex determination and differentiation, as evidenced in goat (*Capra hircus*), where its absence could trigger sex reversal [22]. In most teleosts, *Foxl2* exhibits pronounced sexual dimorphism, with higher expression in ovaries than in testes [3]. In *D. rerio*, the cooperative interaction between *Foxl2a* and *Foxl2b* is essential for regulating ovarian development and maintenance. Dual mutations in these genes lead to sex reversal, which coincides with increased expression of testicular development genes such as *Sox9a*, *Amh*, and *Dmrt1*, along with a reduction in the expression of *Cyp19a1a* and *Cyp11a1* [23]. In gibel carp (*Carassius gibelio*), *Foxl2a-B*, *Foxl2b-A*, and *Foxl2b-B* collectively regulate folliculogenesis and ovarian development. The absence of *Foxl2a-B* may hinder ovarian development and cause sex reversal, while the absence of *Foxl2b-A* and *Foxl2b-B* significantly reduces the number of germ cells [24]. In *O. niloticus*, mutation in *Foxl2* leads to the up-regulation in the expression of genes such as *Sf1*, *Dmrt1*, and *Gsdf*, concurrent with a decrease in the expression of *β-cat1*, *β-cat2*, *Figla*, and *Cyp19a1a*, culminating in reduced estrogen levels and the occurrence of sex reversal [25]. Beyond genetic determinants, sex differentiation is also susceptible to environmental influences, notably the administration of exogenous sex steroid hormones, which can significantly alter gonadal development [26]. For instance, in *O. mykiss*, treatment with 17β-estrogen induces male-to-female sex reversal, marked by the up-regulation of genes crucial for early ovarian differentiation, like *Foxl2*, and the down-regulation of *Amh* expressed in *Leydig* cells for androgen synthesis [27]. Similarly, administration of exogenous androgen could induce female-to-male sex reversal, suppressing the expression of genes like *Cyp19a1* and *Foxl2*, essential for ovarian differentiation, while rapidly up-regulating the male-specific gene, *Dmrt1* [28]. Studies in *O. niloticus* have further confirmed that exogenous estrogen could counteract the sex reversal caused by *Foxl2* deletion [25]. In summary, sex steroid hormones are critical for sex differentiation and gonadal maintenance in teleosts.

As a prominent economic fish in China, blotched snakehead (*Channa maculata*) holds considerable favor among aquaculturists and consumers due to its tender meat, savory taste, few intermuscular spines, and high protein content. Importantly, it displays significant sexual dimorphism, with males exhibiting faster growth rates, larger body sizes, and lower feed coefficient compared to females [29]. Therefore, the cultivation of an all-male population of snakeheads holds substantial economic and ecological value. In our previous study, sex-specific molecular marker and an XX/XY chromosomal sex determination system were identified in *C. maculata* [30]. Meanwhile, XY sex-reversal females, YY super-males and XY all-male populations of *C. maculata* were successfully generated through the amalgamation of sex-specific molecular marker with hormone-induced sex reversal techniques [31]. Furthermore, comparative transcriptome analyses of the gonads of 6-month-old XX normal females, XY normal males, XY sex-reversal females and YY super-males were conducted, revealing numerous candidate genes involved in sex differentiation, gonadal development and growth sexual dimorphism [32]. Nonetheless, the intricate processes governing sex determination, sex differentiation, and gametogenesis in male and female *C. maculata* remain inadequately documented. In pursuit of elucidating the molecular mechanism of sex determination and differentiation in *C. maculata* and discerning the critical time windows for sex control on undifferentiated or differentiating gonads, the current study conducted histological observations and comparative transcriptome analyses on ovaries and testes across various developmental stages. Through this endeavor, the biased differential genes between males and females were identified, and the critical time windows for sex differentiation in each sex from morphological, cytological and molecular levels were determined, respectively. Additionally, significant sexual dimorphism in sex steroid hormones was elucidated. This investigation holds the potential to furnish comprehensive analyses of sex determination and differentiation, thereby establishing a scientific foundation for sex control in this species. Furthermore, the resultant data will serve as valuable genomic resource for innovative breeding strategies aimed at producing high-quality monosex germplasm in *C. maculata*.

## Materials and methods

### Fish and sampling

Blotched snakeheads were reared in the outdoor pond of the Fangcun Experiment Station at the Pearl River Fisheries Research Institute (Guangzhou City, Guangdong Province, China). Artificial reproduction of the stock, fry culture, and fingerling rearing were carried out as previously described [33]. Gonadal samples were systematically collected from individuals across various developmental stages, specifically at 10, 15, 20, 25, 30, 35, 40, 45, 60, 90, 120, 150, and 180 dpf. For each sampling, fish were anesthetized with 1000 mg/L MS-222 (AbMole, USA), and their tail fins were excised and preserved in ethanol for subsequent genetic sex determination as previously described [32]. Given the difficulty in dissecting gonadal samples at 10–45 dpf, every effort was made to meticulously remove the head, tail and muscles, leaving the trunk segment containing the gonad intact for histological examination and RNA extraction. Gonadal samples at 60–180 dpf were bisected, one half was fixed in Bouin’s solution (ABI, USA) for histological analysis, and the other half was promptly snap-frozen in liquid nitrogen for subsequent RNA extraction. Gonadal histology was conducted following the established protocols as described by Wang et al. [34]. After genetic sex determination, a total of 100, 100, 60, 60, and 60 trunk segments containing the gonad for each sex were separately pooled at 10, 15, 20, 25 and 30 dpf. Furthermore, three gonads per sex were selected at 60, 90, 120, 150, and 180 dpf for subsequent experimental analyses. All experiments adhered to animal welfare policies and were approved by the Ethical Committee for Animal Welfare, Pearl River Fisheries Research Institute, Chinese Academy of Fishery Sciences.

### Measurement of body length and weight

Blotched snakeheads were randomly selected from the outdoor pond of the Fangcun Experiment Station at the following developmental stages: 30, 60, 90, 120, 150, 180, and 360 dpf. Their body weight and length were measured with a precision of 0.1 g and 1 mm, respectively. Genetic sex was determined as previously reported [31], ensuring an equal representation of 50 male and 50 female individuals at each developmental stage.

### RNA extraction, library construction, and transcriptome sequencing

Total RNA was isolated from the gonads of males and females at ten developmental stages (10, 15, 20, 25, 30, 60, 90, 120, 150, and 180 dpf) using TriZol reagent (Invitrogen, USA) following the manufacturer’s instructions. The purity and concentration of RNA samples were assessed using a NanoDrop-2000 spectrophotometer (Thermo Fisher Scientific, USA), and their integrity was evaluated with an Agilent 2100/LabChip GX (Agilent Technologies, USA). Sequencing libraries were prepared with the NEBNext® UltraTM RNA Library Prep Kit for Illumina® (New England Biolabs, USA) following the manufacturer’s protocol. A total of 40 sequencing libraries (males at 10, 15, 20, 25, 30, 60, 90, 120, 150, and 180 dpf, and females at 10, 15, 20, 25, 30, 60, 90, 120, 150, and 180 dpf) were sequenced on the Illumina Novaseq 6000 platform. Raw data were processed via Trimmomatic to remove adapters, low-quality bases, and poly-N sequences [35]. Subsequently, clean reads were aligned to the blotched snakehead genome using HISAT2 [36].

### Identification of differentially expressed genes (DEGs)

Transcript quantification was performed by StringTie, adopting the maximum flow algorithm and normalizing with Fragments Per Kilobase of transcript per Million mapped fragments (FPKM), a measure of transcript or gene expression levels [37]. The edgeR and DESeq R packages were employed to identify DEGs across developmental stages [38, 39]. Specifically, edgeR [38] analyzed non-replicated samples from 10 to 30 dpf, and DESeq [39] assessed samples with three biological replicates spanning 60–180 dpf. The false discovery rate (FDR) was controlled using the Benjamini & Hochberg method, with p-values adjusted accordingly. Transcripts with |log_2_[fold change]| ≥ 1 and adjust FDR < 0.01 between any two groups were identified as significant DEGs. DEGs were clustered with Mfuzz in the R package, using a membership score of 0.5 and selecting 12 clusters [40]. UpSet and Venn diagrams were generated via OmicShare tools (www.omicshare.com/tools) to analyze the distribution of DEGs across male and female developmental stages.

### Functional enrichment of DEGs and protein–protein interaction (PPI) network analyses

All DEGs were functionally annotated based on the reference genome of *C. maculata* [41]. To uncover the biological functions and associated pathways of the DEGs, Gene Ontology (GO) and Kyoto Encyclopedia of Genes and Genomes (KEGG) enrichment analyses were performed utilizing the BMKCloud tool (https://www.biocloud.net/). PPI networks were established using STRING (https://string-db.org/), referencing previously reported protein interaction results in *D. rerio* (SRA Accession No. ERS2108068). The PPI network was visualized using Cytoscape (version 3.10.0, https://cytoscape.org/). Chromosome position information was analyzed using MG2C (http://mg2c.iask.in/mg2c_v2.1/).

### Quantitative real-time PCR (qPCR) verification

Eighteen sex-biased genes were selected to validate their expression in the gonads of male and female blotched snakeheads across different developmental stages using qPCR, with specific primers listed in Table 1. cDNA was synthesized using ReverTra Ace® qPCR RT Master Mix with gDNA Remover Kit (Toyobo, Japan) following the manufacturer’s instructions. The StepOnePlus™ Real-Time PCR System (ABI, USA) was employed for qPCR, with each sample assayed in triplicate. *β-Actin* served as the reference gene [42], and the 2^−ΔΔCt^ method was applied to normalize the C*t* values of each reaction [43]. The expression levels of *Amh* in female ovaries at 10 dpf were set as the baseline (1.0) for expression analysis.

**Table 1:** The primers involved in qPCR

Gene name	Primer name	Sequences (5′–3′)	Length (bp)
*Amh*	Amh-DL-F	ATAGAGAGGCTGGGGAGACTCAA	177
	Amh-DL-R	TCTCACTGGATTGTTGGGGTCT	
*Amhr2*	Amhr2-DL-F	ACAATGCCGCTTCAAATAAA	195
	Amhr2-DL-R	CAATGGCTGAGCCCAATGAC	
*Ar*	Ar-DL-F	CGGTGCACTAACCTGTGGTA	201
	Ar-DL-R	GCTCAGGCTCGGTTTAGGAG	
*Cyp17a2*	Cyp17a2-DL-F	CAGTCACTTACCTCATCCATTATCC	283
	Cyp17a2-DL-R	TTTTTCCATTCCTTCTCGTCAT	
*Dmrt1*	Dmrt1-DL-F	TCCCTGACCCCGTCCAAAG	159
	Dmrt1-DL-R	TCGCTGCCTCTCGGCTATC	
*Gsdf*	Gsdf-DL-F	AGGGCGTTGAATTCGTCCAT	92
	Gsdf-DL-R	CCATGATCCCAGCTCACAGG	
*Star*	Star-DL-F	GAACAGGCTGGCAGGTCC	188
	Star-DL-R	CAATGGTCCAGCCGTCCT	
*Sox11a*	Sox11a-DL-F	GGAGACGGTGCTGATGATTT	155
	Sox11a-DL-R	AAGAGGTGGAAGATGCTCAA	
*Bmp15*	Bmp15-DL-F	ATCAAAGCCTCATTCGTCCA	246
	Bmp15-DL-R	GGCAGTGAGGGTCAAGTGTG	
*Ctnd1*	Ctnd1-DL-F	CGCTACCTGTCATCTCTGTGGA	268
	Ctnd1-DL-R	GGAGGTCCGTAGTGGTAGTTGC	
*Ctnd2*	Ctnd2-DL-F	ACATCGGAGCGGAAAGTTAC	209
	Ctnd2-DL-R	TGCCCTCTAAGGTTGGGTTTG	
*Cyp19a1a*	Cyp19a1a-DL-F	AATACCCCTCGTCGTTACTT	137
	Cyp19a1a-DL-R	AAACCCTTATGGAGGCAAA	
*Er*	Er-DL-F	AGGTCCCGCGTCCTCTGTAT	146
	Er-DL-R	CCTGTTGAACCCGTGAATGTG	
*Figla*	Figla-DL-F	CAACGCCAAGGAACGACTG	251
	Figla-DL-R	CTCTCCATAGGTCATTGCCCA	
*Foxl2*	Foxl2-DL-F	GAGAAAAATAAAAAAGGTTGGCA	167
	Foxl2-DL-R	CGGCGTCTCCTGTAGTTCC	
*Pax4*	Pax4-DL-F	CAACGGGTGCGTCAGTAAGA	218
	Pax4-DL-R	TAGACGACACGCTGGGCAC	
*Sox11b*	Sox11b-DL-F	TTGGGCCGGTACTTGTAGTC	99
	Sox11b-DL-R	AATGCTCAAAGACGCGGAGA	
*Sox3*	Sox3-DL-F	ATGCTTATCATATCCCTCAGGTCC	175
	Sox3-DL-R	TGAACGCGGCTTCCACATAC	
*β-Actin*	β-actin-DL-F	AGCAAGCAGGAGTATGATGA	283
	β-actin-DL-R	AGAACGCCAGGGAGTTTTAT	

### Measurement of sex steroid hormone in serum

Blood samples were collected from the caudal vein of male and female snakeheads at different developmental stages. After collection, the samples were allowed to rest at room temperature for 15 min and then centrifuged at 4 °C at 2500 rpm for 15 min to obtain serum, which was subsequently stored at − 80 °C. The levels of E_2_ and T were quantified using the Enzyme-Linked Immunosorbent Assay (ELISA) Kit (NanJing Jiancheng, China) in accordance with the manufacturer’s instructions. Optical density at 450 nm (OD_450_) was measured using Multiskan™ FC microplate reader (Thermo Fisher, USA), and a standard curve was generated to calculate the concentration of E_2_ and T in these samples.

### Statistical analyses

The experimental data were presented as mean ± standard deviation (S.D.). Group differences were evaluated using one-way analysis of variance (ANOVA) followed by Dunnett’s Multiple Range Test using SPSS 26.0. Statistical significance was defined as *P* < 0.05. Additionally, the correlation between body weight and length was analyzed employing SPSS 26.0.

## Results

### Sexual dimorphism in growth of *C. maculata*

**Fig. 1:**
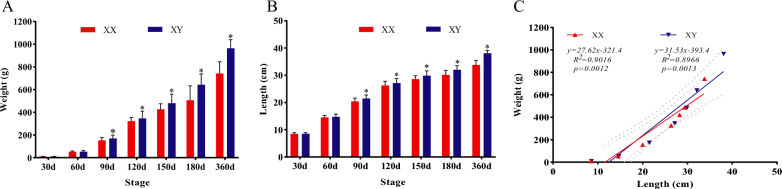
Characterization of sexual dimorphism in growth in blotched snakehead. **A** Body weight of males and females from 30 to 360 dpf, **B** body length of males and females from 30 to 360 dpf, **C** correlation analysis between body length and weight in males and females

To notarize sexual dimorphism in growth, the body weight and length of males and females across multiple developmental stages were measured. Between 30 and 60 dpf, no significant differences in body weight and length were observed between males and females (*P* > 0.05). However, discernible differences in growth emerged from 90 dpf onwards (*P* < 0.05). At 90 dpf, male snakeheads (173.1 ± 29.3 g, 21.5 ± 1.3 cm) were 13.07% heavier and 5.64% longer than females (153.6 ± 25.3 g, 20.4 ± 1.2 cm), respectively. This sexual dimorphism in growth intensified over time, with males ultimately weighing 29.91% heavier and measuring 12.69% longer than females at 360 dpf, respectively (Fig. 1A, B). Notably, a significant positive correlation between body length and weight of male and female snakeheads was evident (Fig. 1C).

### Histology and morphology of gonadal development in *C. maculata*

**Fig. 2:**
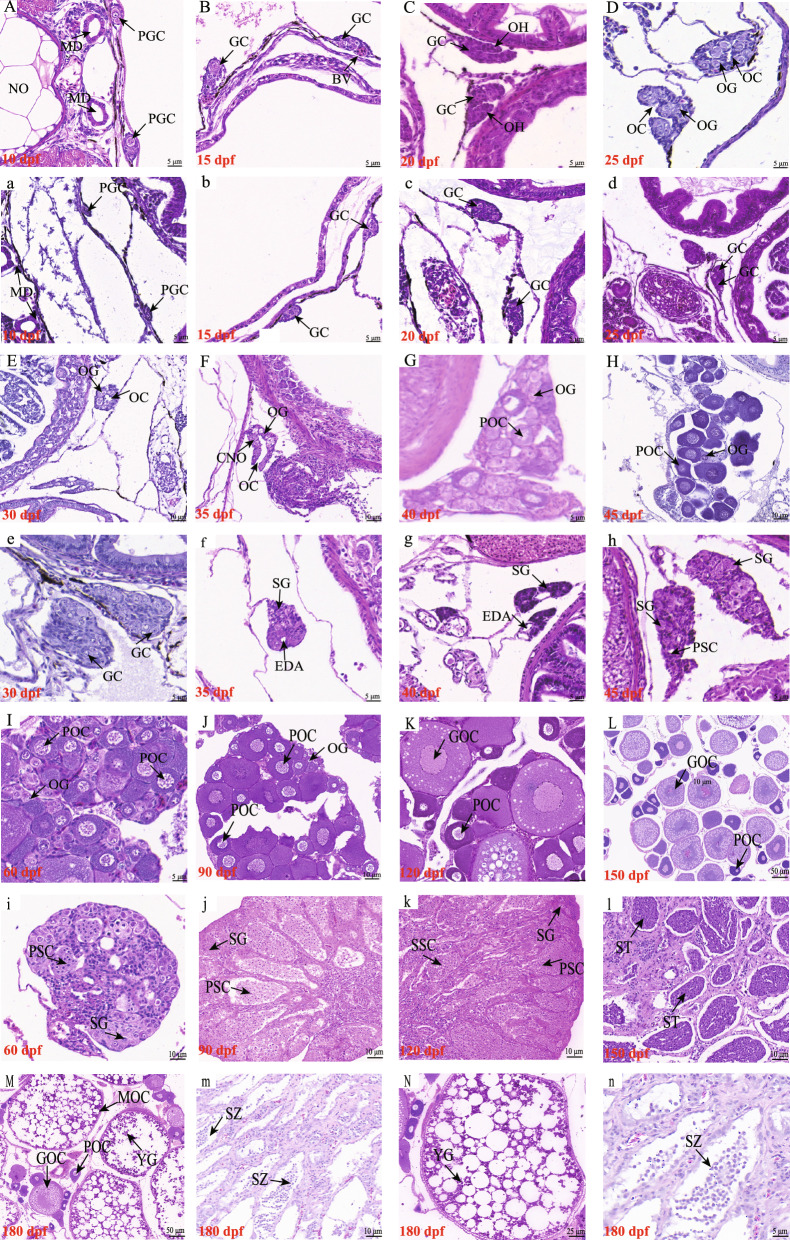
Histological analyses of ovaries and testes across different developmental stages in *C. maculata*. **A**–**M** Represent the ovary at 10, 15, 20, 25, 30, 35, 40, 45, 60, 90, 120, 150 and 180 dpf, respectively; **a**–**m** represent the testis at 10, 15, 20, 25, 30, 35, 40, 45, 60, 90, 120, 150 and 180 dpf, respectively. **N** and **n** show localized magnifications of the ovary and testis at 180 dpf, corresponding to **M** and **m**, respectively. *PGC* primordial germ cell, *MD* mesonephric duct, *NO* notochord, *BV* blood vessel, *GC* germ cell, *OH* organic heave, *OC* ovarian cavity, *OG* oogonia, *SG* spermatogonia, *CNO* chromatin-nucleolus oocyte, *EDA* efferent duct anlage, *POC* primary oocyte, *PSC* primary spermatocyte, *GOC* growing oocyte, *SSC* secondary spermatocyte, *MOC* mature oocyte, *ST* spermatids, *SZ* spermatozoa

To elucidate the interplay between sexual dimorphism and gonadal development, a thorough histological and morphological examination of *C. maculata* gonads were conducted at different developmental stages. Initially, at 10 dpf, primordial gonads in females were observed in pairs beneath the mesonephric duct, containing 1–2 primordial germ cells (PGCs) distinguishable from somatic cells by their large diameter (Fig. 2A). Subsequently, at 15 dpf, somatic cells rapidly proliferated, leading to an increase in the volume of primordial gonads, accompanied by the appearance of blood vessels in females (Fig. 2B). As gonadal development progressed, sectional cell growth and organic heave manifestation occurred at 20 dpf (Fig. 2C), followed by the formation of an incipient ovarian cavity at 25 dpf, alongside further extension of the organic heave, indicating the onset of morphological sex differentiation in females (Fig. 2D). Oogonia was first observed in the germinal epithelium at 25 dpf (Fig. 2D). By 30 dpf, a fully developed ovarian cavity was evident in female gonads, with numerous oogonia undergoing mitosis (Fig. 2E). The chromatin-nucleolus oocytes and continued enlargement of the ovarian cavity were observed at 35 dpf (Fig. 2F). At 40 dpf, primary oocytes emerged in the ovary (Fig. 2G), which exhibited significantly larger volumes compared to oogonia, indicating the onset of cytological ovarian differentiation. Their numbers continued to increase by 45 dpf (Fig. 2H). Between 60 and 90 dpf, the ovary progressed to Stage II, due to the proliferation of primary oocytes and the appearance of growing oocytes (Fig. 2I, J). At 120 dpf, the ovary transitioned to Stage III, assuming an elliptical and cylindrical shape with visible circular eggs. Concurrently, a mass of growing oocytes were observed in the ovary (Fig. 2K). At 150 dpf, a plethora of growing oocytes filled the ovary, exhibiting significantly augmented volumes due to continuous synthesis and accumulation of nutrients, surrounded by two layers of follicular membranes (Fig. 2L). At this stage, oil droplets in growing oocytes increased markedly compared to primary oocytes, while yolk granules began appearing within the oil droplets, scattered in the cytoplasm. By 180 dpf, the ovary advanced to Stage IV, rapidly increasing in size and occupying most of the abdominal cavity. The surface of the ovarian membrane was decorated with blood vessels, and golden-yellow eggs became visible to the naked eye. Mature oocytes predominated, characterized by increased yolk granules and gradual disintegration of the nucleus (Fig. 2M, N).

In contrast, the onset of testicular differentiation in males occurred later than that in ovaries. During the early post-fertilization phase, male juveniles exhibited few PGCs in their primordial gonads, with most remaining inactive in mitosis (Fig. 2a). Between 15 and 30 dpf, both gonad size and germ cell numbers increased dramatically, although no evident morphological signs for testicular differentiation were observed (Fig. 2b–e). A distinct histological transition occurred at 35 dpf, marked by the appearance of the efferent duct anlage and limited round spermatogonia (Fig. 2f), indicating the initiation of morphological sex differentiation in the testis. Subsequently, the number of spermatogonia increased at 40 dpf (Fig. 2g), with primary spermatocytes becoming evident by 45 dpf, signifying the onset of cytological sex differentiation in the testis (Fig. 2h). Compared to spermatogonia, primary spermatocytes were smaller and had more intensely basophilic nuclei. The testis entered Stage II at 60 dpf, with primary spermatocytes arranged in bundles, forming seminiferous lobules (Fig. 2i). By 90 dpf, plentiful secondary spermatocytes were observed within seminiferous lobules, arranged in a regular radial pattern, with a central cavity appearing (Fig. 2j). The testis transitioned to Stage III at 120 dpf, characterized by being light-red, slightly enlarged in volume, and flat in shape, with histological sections displaying the simultaneous presence of spermatogonia, primary spermatocytes, and secondary spermatocytes (Fig. 2k). With ongoing testicular development, the testis volume significantly enlarged, with a darkening color and prominent large blood vessels on the surface, indicative of Stage IV. At 150 dpf, in addition to a few spermatogonia, primary spermatocytes, and secondary spermatocytes, a large number of spermatids appeared with spermatogenetic cysts, nearly filling the entire testis (Fig. 2l). Shortly thereafter, spermatozoa were observed in testis at 180 dpf (Fig. 2m, n).

### Global transcriptome profiles of gonads in *C. maculata*

Comparative transcriptome analyses of gonads in female and male *C. maculata* were conducted across ten distinct developmental stages, spanning from 10 to 180 dpf. After removing low-quality reads, 273.66 Gb high-quality data were obtained from 40 samples (BioProject Accession No.PRJNA1087676), averaging 5.75 Gb per sample, with Q30 values ranging from 90.74% to 97.97%. The clean data were successfully mapped to the high-quality reference genome of *C. maculata* (SRA Accession No. PRJNA730430) [41]. However, during the correlation assessment and principal component analysis (PCA) of samples, one biological replicate (XX-90d-3) showed significant divergence from the other two replicates (XX-90d-1 and XX-90d-2) (Fig. S1A). Specifically, the correlation coefficients between sample XX-90d-3 and samples XX-90d-1 and XX-90d-2 were only 0.08 and 0.06, respectively (Fig. S1B). To ensure the accuracy of the results, sample XX-90d-3 was excluded from the subsequent analyses. In total, 33,266 transcripts were acquired, of which 24,115 corresponded to annotated protein-coding genes, while the remaining 9151 transcripts represented newly identified genes. Inter-sample correlation analysis and PCA revealed notable aggregation in the testes and ovaries transcriptomes before 30 dpf, followed by progressive divergence after 30 dpf (Fig. S1A, C).

### Sex-related DEGs in male and female gonads: identification, spatiotemporal dynamics, functional annotation, and PPI analysis

A total of 72,330 DEGs were identified from pairwise comparisons between male and female samples at the same developmental stages (10, 15, 20, 25, 30, 60, 90, 120, 150, and 180 dpf), with 30,524 up-regulated and 41,806 down-regulated DEGs (Figs. S2, S3). Significantly, the number of up-regulated DEGs rapidly increased from 10 to 60 dpf, sharply decreased at 90 dpf, and then markedly rose again between 120 and 180 dpf. Likewise, down-regulated DEGs increased from 10 to 60 dpf, experienced a slight decline at 90 dpf, and then elevated again between 120 and 180 dpf (Fig. S3). Comparative analyses revealed notable up-regulation of genes such as *Dmrt1*, *Amh*, *Amhr2*, *Star*, *Gsdf*, *Sox9b*, and *Ar* in males compared to females, primarily participating in testicular differentiation and spermatogenesis. Conversely, DEGs associated with ovarian differentiation and oogenesis, including *Foxl2*, *Cyp19a1a*, *Figla*, and *Bmp15*, were significantly up-regulated in females relative to males. To comprehensively investigate the functional roles of DEGs between females and males at each time point, GO and KEGG enrichment analyses were conducted (Figs. S4, S5, Tab. S1–S4).

**Fig. 3:**
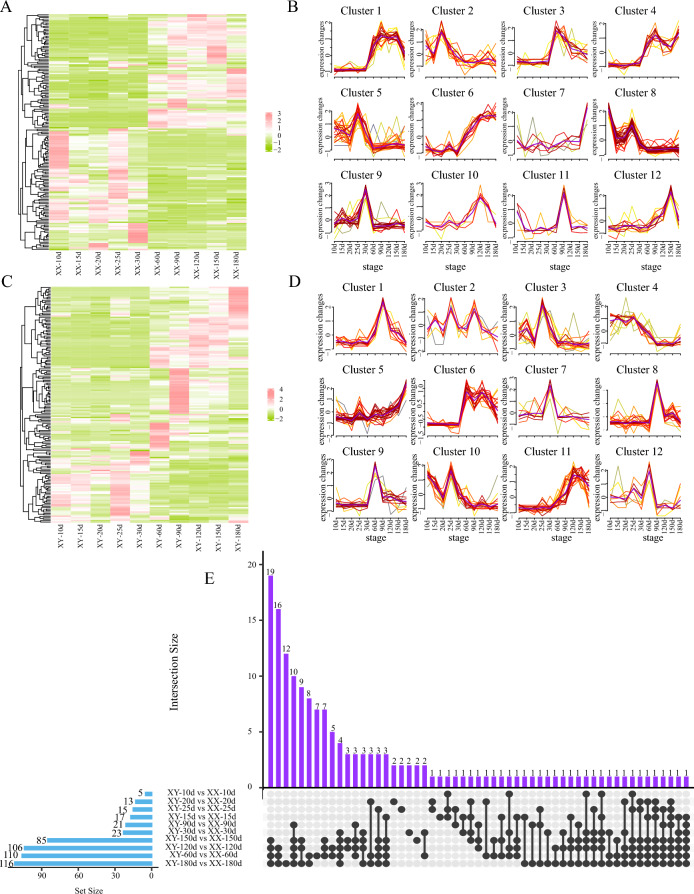
Heatmaps illustrate the expression levels of 165 sex-related DEGs in ovaries (**A**) and testes (**B**), respectively. The cluster analyses of these DEGs across ten key developmental stages in females (**C**) and males (**D**). UpSet network plots reveal the distribution of sex-related DEGs during crucial phases of male and female gonadal development (**E**). The upper bar chart shows the gene count in each group, and the lower left bar chart displays the number of sex-related DEGs at each gonadal development stage

Subsequently, the expression profiles of 165 sex-related DEGs (Tab. S5, S6) were analyzed across ten developmental stages in females (Fig. 3A) and males (Fig. 3B), respectively. To elucidate the temporal dynamics of the transcriptomic datasets across these stages, the Mfuzz clustering method was used to categorize these 165 sex-biased DEGs into 12 clusters (Fig. 3C, D). Notably, the expression patterns observed in Cluster 5 and Cluster 8 of the female samples were particularly noteworthy, wherein pivotal transcription factors including *Sox8*, *Sox9b*, *Sox11a*, *Sox17*, *Foxo3*, *Gata3*, and *Lhx8* decreased sharply from 10 dpf, surged again between 20 and 25 dpf, and then sharply declined and manifested low expression levels between 60 and 180 dpf, suggesting a potential role in early ovarian differentiation (Fig. 3C). Additionally, significant up-regulation in the transcription levels of meiosis marker genes, such as *Sycp3* and *Spo11*, was observed in Clusters 3 and 4 during 30–60 dpf. This up-regulation is consistent with the observation of primary oocytes at 40 dpf, reinforcing the pivotal role of these genes in the onset of meiotic process. Key genes involved in follicular development in Cluster 3 (e.g., *Figla*, *Wnt4*, *Zp4*, *Hsd17b12*, *Er*) were not expressed from 10 to 30 dpf, but showed continuous expression from 60 to 180 dpf (Fig. 3C), revealing the crucial roles of these genes in female gonadal development. In contrast, major male-differentiation-related genes in Cluster 6 (*Dmrt1*, *Dmrtb1*, *Amhr2*, *Star*, *Ar*) were prominently expressed between 30 and 60 dpf (Fig. 3D), aligning with the commencement of morphological testicular differentiation at 35 dpf (Fig. 2f) and cytological testicular differentiation at 45 dpf (Fig. 2h).

To better understand the interactions among these 165 sex-related DEGs over ten developmental stages, an UpSet plot was constructed (Fig. 3E). Notably, *Star* exhibited pronounced male-biased expression spanning six developmental stages post 30–180 dpf, suggesting its potential significance in the regulation of testicular development. Additionally, *Figla*, *Sox11b*, *Cyp19a1a*, and *Ctnd2* showed significant female-biased expression between 60 and 180 dpf, likely related to the development and maintenance of female gonads. Meanwhile, *Sox3* and *Sox11a* exhibited significant male-biased expression prior to 30 dpf, suggesting their crucial role in early testicular differentiation.

**Fig. 4:**
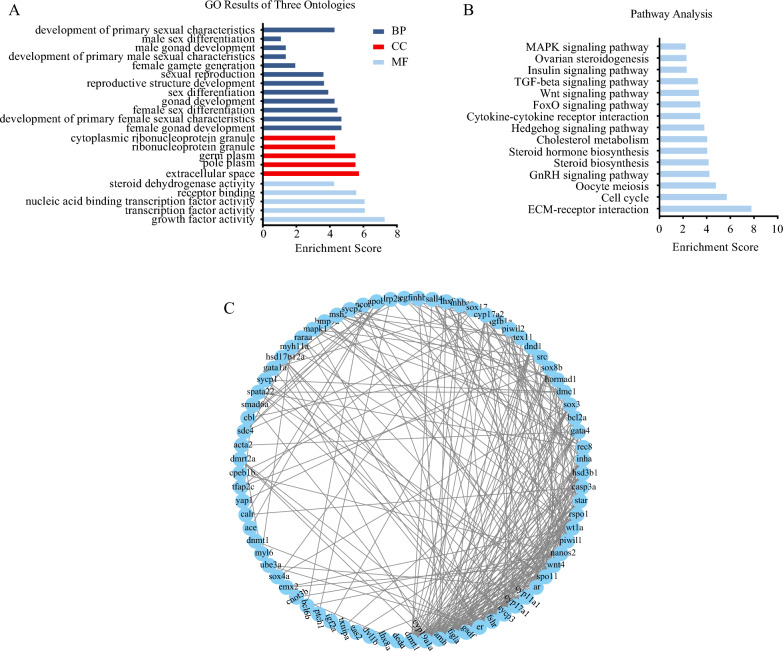
The GO (**A**), KEGG enrichment (**B**) and PPI network (**C**) analyses of 165 sex-related DEGs during gonadal development of the ovaries and testes

The GO and KEGG functional enrichment analyses were performed on these 165 sex-related DEGs. Several sex-related GO terms were identified, including development of primary sexual characteristics (GO:0045137), sex differentiation (GO:0007548), female gonad development (GO:0008585), and female sex differentiation (GO:0046660) (Fig. 4A, Tab. S7). Additionally, significantly enriched KEGG pathways were observed, such as ECM-receptor interaction (ko04512), Cell cycle (ko04110), Oocyte meiosis (ko04114), and GnRH signaling pathway (ko04912) (Fig. 4B, Tab. S8). To further explore the interrelationships among selected DEGs, a PPI network was developed, encompassing pivotal sex-related genes like *Dmrt1*, *Amh*, *Foxl2*, *Cyp19a1a*, *Wnt4*, *Er*, and *Figla*. Within this network, 10 core genes, including *Dmrt1*, *Amh*, *Cyp19a1a*, *Gsdf*, *Er*, *Figla*, *Ar*, *Fshr*, *Cyp11a1*, and *Cyp17a1*, emerged as being highly interconnected (Fig. 4C).

### DEGs in female gonads: identification, spatiotemporal dynamics, functional annotation, and PPI analysis

**Fig. 5:**
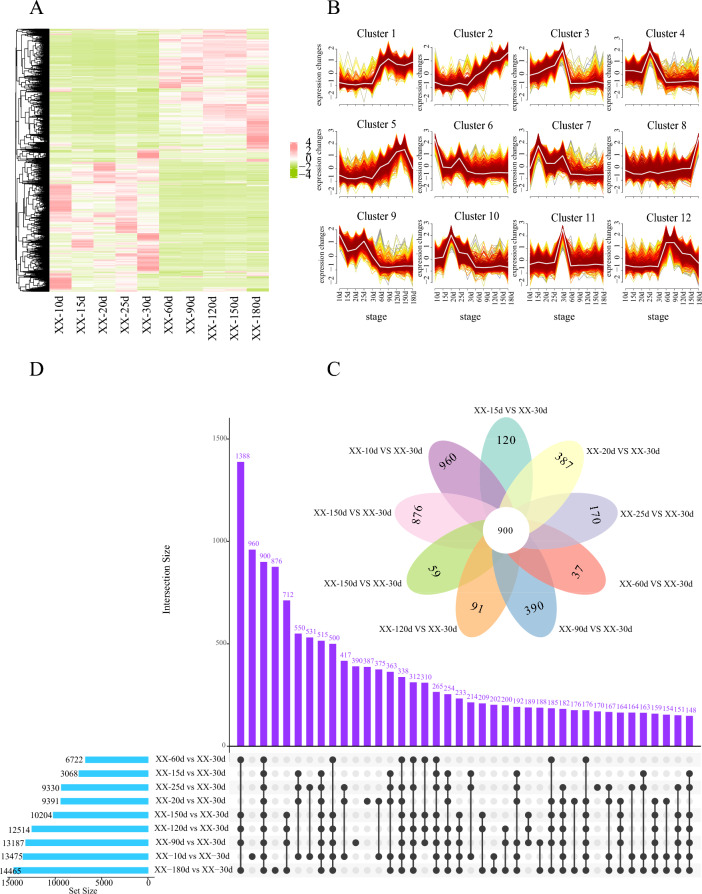
Heatmap illustrates the expression levels of all DEGs across ten key stages of ovarian development (**A**), cluster analysis of DEGs at ten critical periods in ovarian development using Mfuzz (**B)**, Venn (**C**) and UpSet plots (**D**) show the distribution of DEGs at ten critical periods in ovarian development. The upper bar chart shows the gene count in each group, and the lower left bar chart displays the number of sex-related DEGs at each gonadal development stage

Comparative analyses of ovarian tissues across nine female sample groups (10, 15, 20, 25, 60, 90, 120, 150, and 180 dpf) relative to 30 dpf ovary benchmark revealed a total of 96,716 DEGs, including 50,854 up-regulated and 45,862 down-regulated DEGs (Fig. 5A, S6A). These DEGs were classified into 12 distinct clusters, each showing discernible expression patterns, as illustrated in Fig. 5B.

In Cluster 9, *Sox17*, *Bmp2*, *Gnrh3*, and *Tfap2c* exhibited high expression levels at 10 dpf, followed by a decline, reaching another peak at 25 dpf before rapidly decreasing (Fig. 5B). These DEGs are associated with the development of PGCs and female germ cells (FGCs). The findings suggest that the initial time of early ovarian differentiation occurs at 20–25 dpf or earlier, consistent with histological observations where the ovarian cavity and oogonia were first observed at 25 dpf (Fig. 2D). Well-known genes involved in FGCs development and ovarian differentiation, such as *Dazl*, *DDx4*, *Sycp3*, and *Spo11* in Cluster 1, exhibited a sharp increase from 30 to 90 dpf, followed by a slight decrease in expression levels, but they remained relatively high. Similarly, other well-known genes involved in FGCs development and female gonadal differentiation, also observed in Cluster 12, like *Nanog*, *Sall4*, *Sycp1*, *Sycp2*, *Figla*, and *Zar1*, exhibited a sharp increase from 30 to 60 dpf, maintained high expression levels at 90 dpf, and subsequently declined gradually. Combining the first observation of primary oocytes at 40 dpf (Fig. 2G), it is inferred that molecular ovarian differentiation occurs between 40 and 60 dpf. Importantly, DEGs involved in estrogen synthesis were also identified in Cluster 1, such as *Foxl2*, *Cyp19a1a*, *Zp3* and *Zp4*. In Cluster 5, the expression levels of *Gdf9*, *Sox3*, and *Bmp15* continuously increased from 30 to 150 dpf and slightly decreased at 180 dpf, mainly involved in the follicle growth/vitellogenesis process. Additionally, DEGs involved in the response to steroid hormone were also identified, including *Er*, *Pgrmc1*, and *Pgrmc2*.

**Fig. 6:**
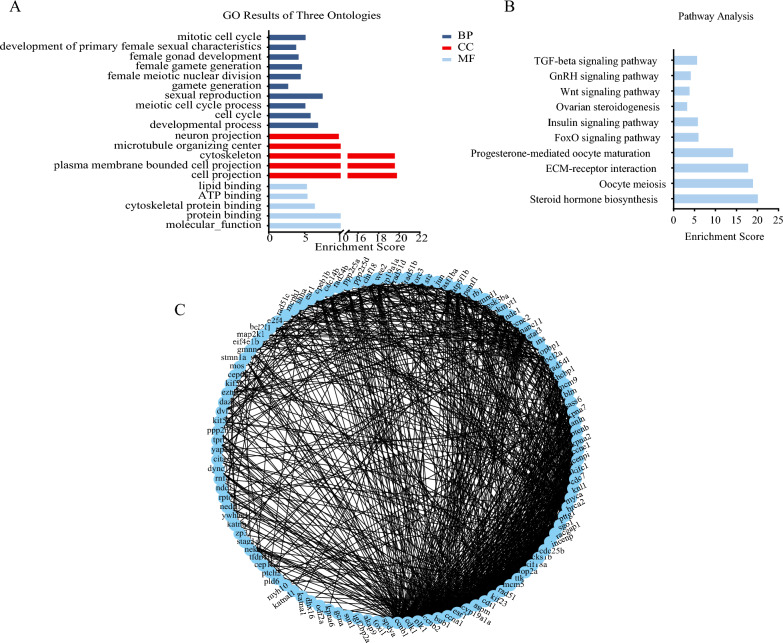
The GO (**A**), KEGG enrichment (**B**) and PPI network (**C**) analyses of all DEGs at ten key stages of ovarian development

Based on UpSet and Venn diagram analyses (Fig. 5C, D), a total of 900 overlapping DEGs were identified across ten female developmental stages, encompassing critical genes involved in female gonadal differentiation and steroid hormone biosynthesis, notably including *Cyp1a1*, *Hsd11b1*, *Cyp7b1*, *Akr1d1*, *Ugt1a1*, *Ugt2a3*, *Sult1a4* and *Sts*. Furthermore, DEGs at various developmental stages in females underwent analysis for GO and KEGG pathways. The pivotal GO terms associated with sex included sexual reproduction (GO:0019953), female gonad development (GO:0008585), and female gamete generation (GO:0007292), as shown in Fig. 6A and Tab. S9. The majority of identified KEGG pathways were linked to steroid hormone biosynthesis (ko00140), oocyte meiosis (ko04114), and ECM-receptor interaction (ko04512), as indicated in Fig. 6B and Table S10. PPI analysis highlighted central genes, such as *Cyp19a1a*, *Er*, *Ccnb1*, and *Cdk1* (Fig. 6C).

### DEGs in male gonads: identification, spatiotemporal dynamics, functional annotation, and PPI analysis

In male samples, a total of 51,681 DEGs were revealed through comparisons between 30 dpf and other developmental stages (10, 15, 20, 25, 60, 90, 120, 150, and 180 dpf), comprising 26,283 up-regulated and 25,398 down-regulated DEGs. Remarkably, there was a discernible upward trend in the number of DEGs as testicular development progressed, indicating an increasing involvement of genes in the regulatory processes of testicular differentiation and development (Fig. S6B).

**Fig. 7:**
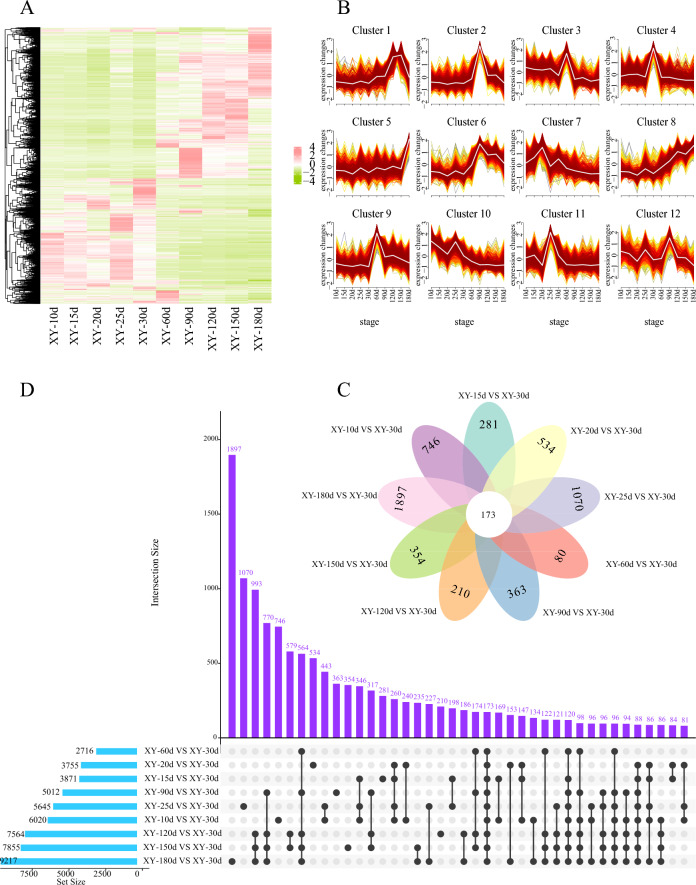
Heatmap illustrates the expression levels of all DEGs across ten key stages of testicular development (**A**), cluster analysis of DEGs at ten critical periods in testicular development using Mfuzz (**B**), Venn (**C**) and UpSet plots (**D**) show the distribution of DEGs at ten critical periods in testicular development. The upper bar chart shows the gene count in each group, and the lower left bar chart displays the number of sex-related DEGs at each gonadal development stage

Temporal trend analysis revealed 12 unique expression profiles for these DEGs in males. Marker genes present in PGCs and the undifferentiated spermatogonia, including *Tfap2c*, *Tet1*, *Bmp2*, *Id4*, *Gnrh3* and *Gfar1*, showed significant up-regulation in Cluster 7 from 10 to 30 dpf, despite minor fluctuations in expression levels at certain intervals (Fig. 7A, B). Subsequently, their expression levels declined, remaining relatively low. Notably, the expression levels of *Dmrt1* and *Hormad1* in Cluster 9 were significantly increased within 30–60 dpf (Fig. 7B). Combined with the initial detection of the efferent duct anlage and spermatogonia at 35 dpf (Fig. 2f), this suggests a potential occurrence of early testicular differentiation between 30 and 35 dpf or earlier. The expression of numerous meiosis-related genes (e.g., *Spo11*, *Sycp3*, *Spag6*, *Dmc1*) in Cluster 6 sharply increased from 60 dpf, peaked at 90 days, then gradually declined, but remained at high levels until decreasing again at 150 dpf. Given the first observation of secondary spermatocytes at 90 dpf, it is indicated that 60–90 dpf may be the key time window for molecular testicular differentiation. Furthermore, DEGs involved in testicular development, such as *Amh* and *Star*, were also identified in Cluster 6*.* Notably, *Ar* and *Cyp17a2* in Cluster 8 continued to rise (Fig. 7B), which are associated with responses to steroid hormone stimulation.

**Fig. 8:**
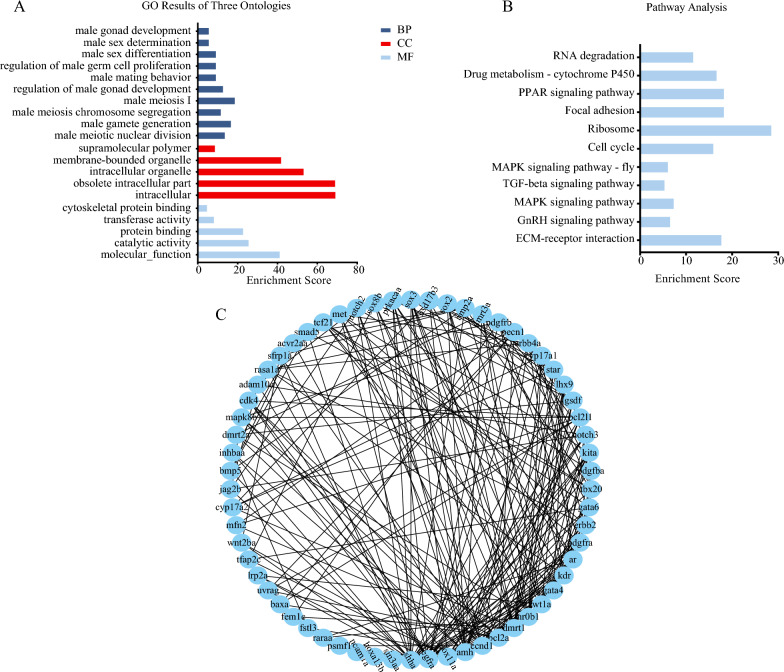
The GO (**A**), KEGG enrichment (**B**) and PPI network (**C**) analyses of all DEGs at ten key stages of testicular development

Utilizing UpSet and Venn diagram analyses (Fig. 7C, D), a consensus of 173 DEGs shared across ten male developmental stages were identified. This cohort of DEGs was implicated in steroid metabolism processes, including *Star*, *Nrob2*, *Npc1l1*, *Ugt2a3*, *Apob*, *Malrd1* and *Igf1*. GO functional analysis revealed that terms related to male reproductive development were primarily enriched in categories such as male meiosis I (GO:0007141), male gamete generation (GO:0048232), and male sex differentiation (GO:0046661) (Fig. 8A, Table S11). Likewise, KEGG enrichment analysis identified key pathways such as ECM-receptor interaction (ko04512), MAPK signaling (ko04010), and TGF-β signaling (ko04350), as indicated in Fig. 8B and Table S12. To further explore the interactions among these DEGs, the PPI network was constructed, identifying six central genes: *Dmrt1*, *Amh*, *Ccnd1*, *Nrob1*, *Bcl2a*, and *Wt1* (Fig. 8C).

Subsequently, we identified a subset of 81 overlapping genes (Fig. S7A) shared between 900 overlapping DEGs in females (Fig. 5C) and 173 in males (Fig. 7C). These genes exhibited significantly biased expression in the 30 dpf ovaries, despite their lower expression levels at other developmental stages (Fig. S7B). GO enrichment analysis revealed that these 81 overlapping genes were predominantly enriched in the brush border (GO:0005903), clusters of actin-based cell projections (GO:0098862), response to lipopolysaccharide (GO:0032496), and transaminase activity (GO:0008483) (Fig. S7C). Furthermore, KEGG enrichment analysis identified several key metabolic pathways, including fat digestion and absorption (ko4975), cholesterol metabolism (ko4979), and phenylalanine, tyrosine, and tryptophan biosynthesis (ko00400) (Fig. S7D).

### Validation and structural analyses of sex-related DEGs

**Fig. 9:**
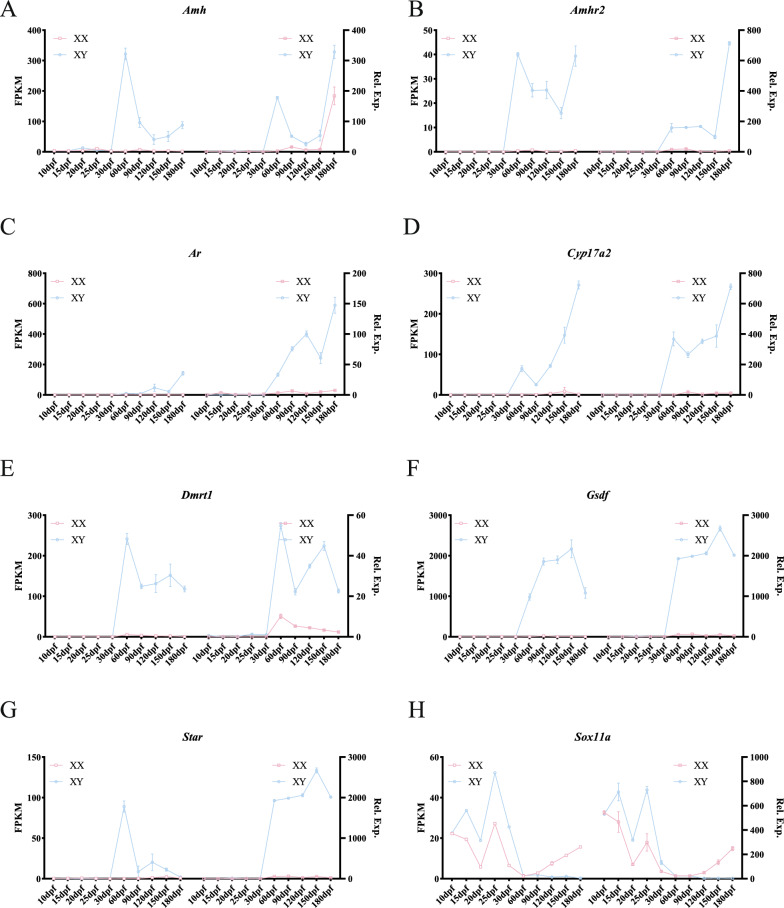
qPCR analyses of critical male-biased DEGs during gonadal development. **A** *Amh*, **B** *Amhr2*, **C** *Ar*, **D** *Cyp17a2*, **E** *Dmrt1*, **F** *Gsdf*, **G** *Star* and **H** *Sox11a*. The results of RNA-seq and qPCR in each panel were shown in the left and right, respectively

**Fig. 10:**
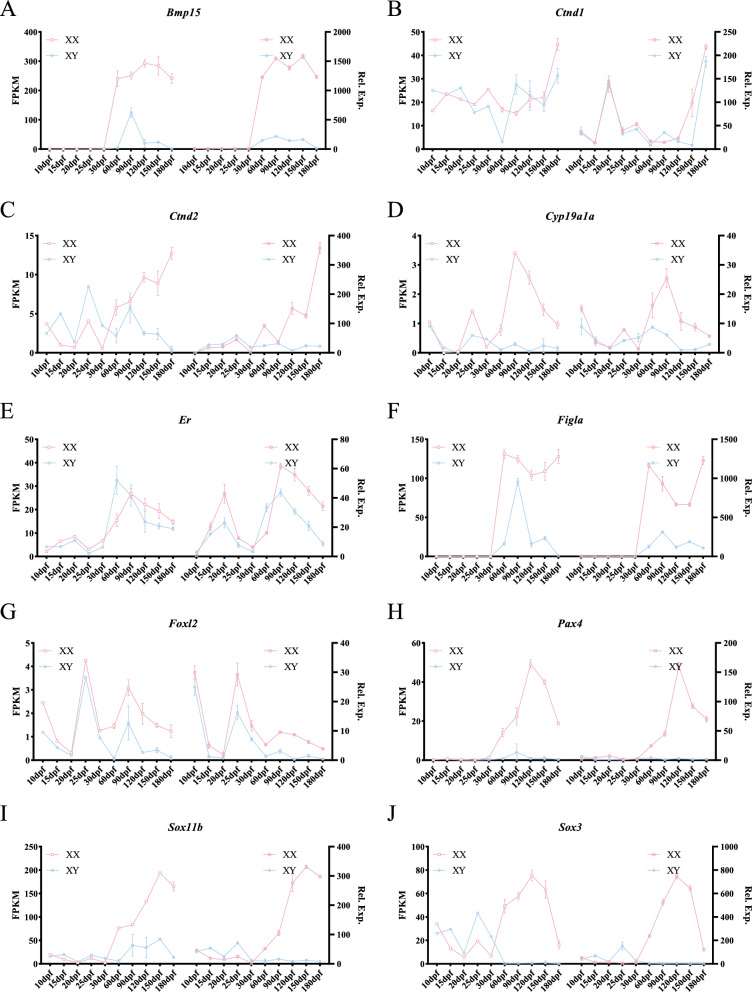
qPCR analysis of critical female-biased DEGs during gonadal development. **A** *Bmp15*, **B** *Ctnd1*, **C** *Ctnd2*, **D** *Cyp19a1a*, **E** *Er*, **F** *Figla*, **G** *Foxl2*, **H** *Pax4*, **I** *Sox11b*, **J** *Sox3*. The results of RNA-seq and qPCR in each panel were shown in the left and right, respectively

To validate the accuracy of our transcriptome data, 18 sex-biased DEGs were randomly selected, and their expression patterns in male and female gonads across different developmental stages (10, 15, 20, 25, 30, 60, 90, 120, 150, and 180 dpf) were analyzed. The expression trajectories of these DEGs were visually depicted over time and space, along with their chromosomal locations. Figures 9 and 10 demonstrate that the expression trajectories from qPCR analyses closely aligned with those inferred from RNA-seq data. Our analysis revealed that *Amhr2*, *Amh*, *Dmrt1*, *Cyp17a2*, *Ar*, *Gsdf*, and *Star* were situated on chromosomes LG05, LG09, LG13, LG15, LG16, LG17, and LG20, respectively (Fig. S8), exhibited significantly higher expression in males (Fig. 9), indicating their crucial roles in male sex differentiation and spermatogenesis. Notably, *Sox11a*, located on sex chromosome LG02 [30], exhibited high expression in males before 30 dpf, which is the key period of early sex differentiation. Then, its expression decreased sharply at 60 dpf in both sexes. Subsequently, its expression in males was extremely low, while its expression in ovaries gradually increased after 90 dpf. Conversely, *Er*, *Cyp19a1a*, *Ctnd1*, *Foxl2*, *Sox3*, *Figla*, *Ctnd2*, *Bmp15*, *Sox11b*, and *Pax4*, located on chromosomes LG02, LG04, LG06, LG08, LG12, LG13, LG14, LG15, LG18, and LG21, respectively (Fig. S8), showed female-biased expression patterns (Fig. 10), suggesting their significance in female sex differentiation and oogenesis.

### The levels of sex steroid hormone in serum

**Fig. 11:**
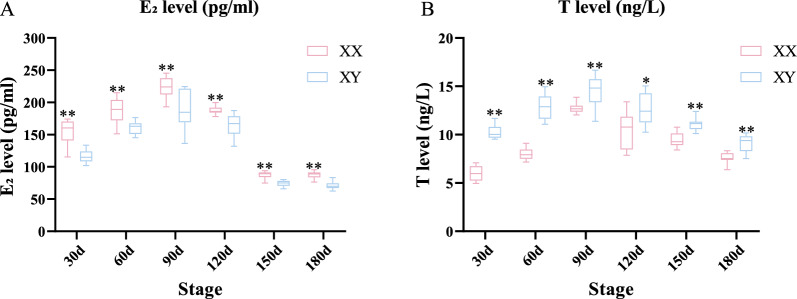
Comparison of E_2_ (**A**) and T (**B**) levels across six developmental stages in female and male snakeheads. Asterisks represent significant differences between males and females. *P < 0.05, and **P < 0.01

The fish ELISA kit was utilized to ascertain the levels of E_2_ and T during gonadal differentiation and development in male and female snakeheads. E_2_ levels exhibited pronounced sexual dimorphism, being significantly more abundant in females, suggesting that E_2_ may play a pivotal role in female gonadal differentiation and maintenance (Fig. 11A). Likewise, T levels in males were significantly higher than those in females, highlighting the potential critical role of T in male sex differentiation and maintenance (Fig. 11B).

## Discussion

Growth stands as one of the most economically valuable traits for genetic improvement in fish. During the early stages of embryonic development in fish, germ cells segregate from somatic cells. Somatic cells are responsible for the development and growth of most organs, while gonadal development is primarily driven by the proliferation of germ cells [2]. An antagonism between gonadal development and somatic growth is commonly observed in teleosts. For instance, in species such as *C. carpio* [16], Japanese flounder (*Paralichthys olivaceus*) [44], and *C. semilaevis* [12], ovaries typically mature later than testes, with most nutrients being converted into body growth during sexual maturation, resulting in females exhibiting larger sizes compared to males. Conversely, in species like *O. niloticus* [10], yellow catfish (*Pelteobagrus fulvidraco*) [45], *I. punctatus* [14], and four-eyed sleeper (*Bostrychus sinensis*) [46], testes mature later than ovaries, and males demonstrate faster growth rates during sexual maturation, consequently leading to larger male individuals. In this study, male individuals of *C. maculata* exhibited larger body sizes compared to females since 90 dpf, with differences accentuating further upon sexual maturity at 360 dpf (Fig. 1), similar to *O. niloticus* [10], *P. fulvidraco* [45], *I. punctatus* [14], and *B. sinensis* [46]. In teleost, female early sex differentiation is marked by the formation of the ovarian cavity, while male early sex differentiation is denoted by the formation of the efferent duct anlage [34]. In *C. maculata*, the ovarian cavity was first observed at 25 dpf (Fig. 2D), while the efferent duct anlage first emerged at 35 dpf (Fig. 2f). It is speculated that the periods between 25 and 35 dpf represent the critical time window to determine the fate of undifferentiated primordial gonads, and sexual dimorphism in growth becomes apparent 2 months after gonadal sexual dimorphism. The earlier initiation of ovarian differentiation may divert a significant amount of energy from somatic growth, potentially contributing to the observed sexual dimorphism in growth of *C. maculata*. Similar observations were reported in *B. sinensis*, where the initiation of oogenesis preceded spermatogenesis by at least 1 month [46]. Transcriptome analyses conducted on gonadal tissues across different developmental stages provided further support for this finding. The gene expression patterns of testes and ovaries exhibited similarity between 10 and 30 dpf, but subsequently diverged in opposite directions after 30 dpf (Fig. S1). Therefore, the period preceding 30 dpf emerges as a critical time window for implementing sex steroid hormones to induce sex reversal in *C. maculata*, which is vital for sex control in this species.

Transcription factors serve a crucial role in the genetic network governing sex determination, sex differentiation and gametogenesis, ensuring the proper functioning of chromosomal mechanism [8]. Among these factors, the *Sox* gene family holds particular significance in growth, development, sex determination and differentiation, and neurogenesis [47]. Since the identification of *Sry* as the sex-determining gene in mammals, extensive research has explored the functions of other members of the *Sox* gene family in mammals [48]. Due to unique whole-genome duplication events, fish possess a greater number of *Sox* genes compared to other animals [49]. *Sox3* has been identified as the core gene for male sex determination in *O. dancena* [11], exerting significant roles in testicular differentiation, steroidogenesis, and spermatogenesis [49], and also holds a crucial role in oogenesis [50]. In *O. niloticus*, *Sox11a* is involved in the regulation of gonadal differentiation, whereas *Sox11b* plays a pivotal role in spermatogenesis and vitellogenesis [49]. Our study revealed that *Sox3* and *Sox11a* exhibit high expression levels during early sex differentiation (10–30 dpf), predominantly in males (Figs. 9H, 10J). In our previous study, the high-density linkage map and QTL suggest that LG02 may be the sex-related chromosome in *C. maculata* [30]. Genome-wide association studies on 500 snakeheads (250 females and 250 males) using GEMMA software’s MLM model identified the sex-determining region on chromosome 2 (unpublished data). Combined with the previous transcriptome analysis of the gonads of 6-month-old snakeheads [32], it was discovered that *Sox11a* was a sex-biased DEG located in the sex-determining region (unpublished data). In this study, chromosome position analysis using MG2C further confirmed that *Sox11a* is located on LG02 (Fig. S8). These data lead us to hypothesize that *Sox11a* may serve as a potential sex-determining gene in *C. maculata*. Further research on *Sox11a* is certainly warranted to test this hypothesis. For instance, CRISPR/Cas9 can be employed to knock out *Sox11a*, allowing for a more in-depth investigation of its role in sex determination and differentiation. *Sox3* expression consistently increased during ovarian differentiation and vitellogenesis (30–120 dpf) (Fig. 8J), consistent with findings in European sea bass (*Dicentrarchus labrax*) [51] and *O. latipes* [11], indicating that *Sox3* plays a critical role in ovarian differentiation and oogenesis in *C. maculata*. As for *Sox11b*, its expression significantly increased after 60 dpf (Fig. 10I) and remained elevated during the stages of vitellogenesis, aligning with the findings in *O. niloticus* [49]. Overall, the *Sox* gene family plays a critical role in sex differentiation and gametogenesis in *C. maculata*. However, further investigations are needed to understand the specific molecular mechanism and interactive networks involved.

Once the master sex-determining gene initiates its expression, it subsequently activates downstream sex-differentiation-related genes, ultimately leading to the synthesis of sex steroid hormones that control the fate of bipotential gonads through synergistic actions [45]. In our investigation, we unveiled sexually dimorphic expression patterns of male-biased and female-biased genes during gonadal differentiation in *C. maculata*. *Dmrt1*, a pivotal transcription factor in sex determination and differentiation, is regarded as one of the most ancient sex-related genes. It exhibits specific and high expression in the testes of various fish species, including *O. niloticus* [50], *O. latipes* [5], *Schizothorax kozlovi* [52], and *C. semilaevis* [53]. In *O. niloticus*, the absence of *Dmrt1* leads to spermatogonia degeneration and abnormal testicular development, accompanied by a significant diminution in the expression levels of *Amh*, *Gsdf*, and *Sox9b* in the testes of homozygous mutants, alongside a substantial elevation in the expression of *Foxl2*, *Cyp19a1a*, and *42sp50* [50]. Similarly, the deficiency of *Dmrt1* can instigate sex reversal in *C. semilaevis*, resulting in a marked increase in the expression of female-associated genes *Foxl2* and *Cyp19a1a* in the gonads of *Dmrt1*^*−/−*^ homozygous mutants, whereas the expression levels of male-associated genes *Sox9a* and *Amh* are significantly reduced [53]. In our study, *Dmrt1* emerged as a male-biased DEG. In males, it was almost not expressed at 10–30 dpf, dramatically increased at 30–60 dpf, slightly decreased at 90 dpf, then up-regulated to another peak at 150 dpf, while remaining minimal in females (Fig. 9E). Comparable observations have been noted in *C. carpio* [16], *O. niloticus* [54], and spotted scat (*Scatophagus argus*) [55], demonstrating the significant role of *Dmrt1* in testicular differentiation and gonadal maintenance in *C. maculata*.

*Amh* also plays a crucial role in male sex determination and differentiation. Specifically, *Amh* binding to its receptor, Amhr2, facilitates the transcription of downstream target genes, thereby regulating the synthesis of sex steroid hormones and the meiotic division of germ cells [56]. In *O. hatcheri*, knockdown of the *Amh* replica on specific Y chromosome (*Amhy*) results in up-regulation of *Foxl2* and *Cyp19a1a* mRNA and ovarian differentiation [9]. In *O. niloticus*, knockdown of *Amh* leads to the significant reduction in the transcription levels of *Dmrt1*, *Gsdf*, and *Cyp19a1a* [57]. In this study, *Amh* expression sharply increased in males between 30 and 60 dpf, then decreased sharply and rose again from 120 dpf, with minimal expression in females. Concurrently, both *Foxl2* and *Cyp19a1a* maintained low expression levels in male gonads. We speculate that *Dmrt1* and *Amh* play crucial roles in the male gonadal differentiation and maintenance in *C. maculata* by suppressing the expression of *Foxl2* and *Cyp19a1a*. Furthermore, *Sox9* plays a crucial regulatory role in sex differentiation and gonadal development, with its absence in mice leading to male-to-female sex reversal [58]. Owing to whole-genome duplication events, two duplicate genes of *Sox9* have been identified in fishes such as *D. rerio* [59], fugu (*Takifugu rubripes*) [60], and *P. olivaceus* [61]. Further investigation revealed the presence of *Sox9a* transcripts in *Sertoli* cells of the zebrafish testes, whereas *Sox9b* was detected in the ovaries [59]. In *T. rubripes*, the expression level of *Sox9a* is higher in testes than in ovaries, while *Sox9b* is exclusively detected in ovaries [60]. Similarly, two *Sox9* genes exist in *C. maculata*, where *Sox9a* exhibited low expression levels in both male and female gonads without significant differences, akin to *C. carpio* [16]. In contrast, *Sox9b* showed biased expression in testes during 25–180 dpf (Fig. 3D). This suggests that *Sox9b* may play a pivotal role in the testicular differentiation and maintenance in *C. maculata*. Additionally, we identified several other male-biased genes, such as *Cyp17a2*, *Gsdf*, and *Star*, which play significant roles in male gonadal development. Overall, our findings suggest that the *Sox11a*–*Dmrt1*–*Sox9b* pathway may activate downstream sex-differentiation-related genes, thereby facilitating male gonadal development and testes formation.

**Fig. 12:**
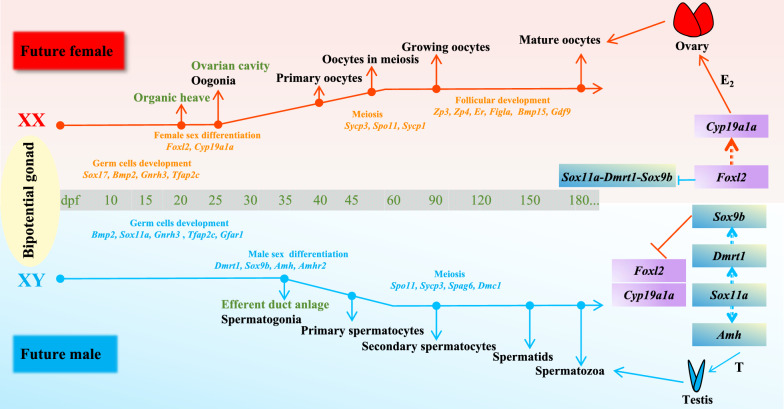
Overview of key genes and potential pathways that bipotential gonads develop to either testis and ovary development in *C. maculata*. In females, the expression levels of *Foxl2* and *Cyp19a1a* began to increase at 20 dpf. In males, the expression levels of *Sox11a*, *Dmrt1* and *Sox9b* began to increase at 20, 30, and 30 dpf, respectively. The ovarian cavity and efferent duct anlage were first observed at 25 dpf and 35 dpf in female and male gonads, respectively. Therefore, the periods of 20–25 dpf or earlier and 30–35 dpf or earlier were the morphological ovarian and testicular differentiation, respectively. *Sycp3* and *Spo11* exhibited a sharp increase from 30 to 90 dpf in female gonads, combining the first observation of primary oocytes at 40 dpf, it is inferred that molecular ovarian differentiation occurs at 40–60 dpf. In contrast, *Sycp3* and *Spo11* sharply increased from 60 to 90 dpf, with the first observation of secondary spermatocytes at 90 dpf, indicating that 60–90 dpf may be the key time window for molecular testicular differentiation. In general, it is hypothesized the fate of undifferentiated primordial gonads in *C. maculata* might be determined by the antagonistic action of the *Sox11a*–*Dmrt1*–*Sox9b* and *Foxl2/Cyp19a1a* pathways. Dashed arrows with direction indicate regulatory interactions between genes, and T-shaped arrows represent antagonistic relationships

The research demonstrated that key genes involved in female sex differentiation and oogenesis, notably *Foxl2*, *Cyp19a1a*, *Bmp15*, and *Figla*, exhibited a more pronounced expression in females compared to males (Fig. 10). Many female-differentiation-related genes, such as *Foxl2* and *Cyp19a1a*, commence expression when germ cells transform into oogonia under the crosstalk of germ cells and somatic cells [15]. In this study, *Foxl2* expression exhibited a marked increase in females between 20 and 25 dpf, followed by down-regulation and subsequent increase at 60 dpf (Fig. 10G). Similar expression patterns were also observed in *C. carpio* [16], *D. rerio* [23], and *O. niloticus* [25], where *Foxl2* expression initiates early ovarian differentiation and persists into adulthood. Histological observations revealed the first appearance of ovarian cavity at 25 dpf, indicating that 20–25 dpf may mark the onset of morphological ovarian differentiation. These results suggest that *Foxl2* plays an important role in female sex differentiation and maintenance in *C. maculata.* Importantly, as a key gonadal aromatase involved in ovarian differentiation, the expression pattern of *Cyp19a1a* mirrored that of *Foxl2* in *C. maculata*, accompanied by an increase in serum E_2_ levels during ovarian differentiation (30–90 dpf) (Fig. 11A). Notably, studies have shown that *Foxl2* can regulate *Cyp19a1a* expression to produce E_2_ for ovarian differentiation [16, 25, 62]. Homozygous mutants of *Foxl2*^*−/−*^ or *Cyp19a1a*^*−/−*^ in XX *O. niloticus* displayed female-to-male sex reversal, with reduced germ cell numbers and up-regulated expression levels of male pathway genes such as *Sf1*, *Dmrt1*, and *Gsdf*. Furthermore, this mutant phenotype could be rescued by exogenous E_2_ treatment [25]. In this study, *Dmrt1* was expressed later than *Foxl2*, sharply increasing after 30 dpf, supporting the notion that *Foxl2* is epistatic to *Dmrt1*, as evidenced by the underdeveloped testes resulting from the double mutation of *Foxl2*/*Dmrt1* [50]. Based on these findings, a potential pathway leading from bipotential gonads to either testicular or ovarian development in *C. maculata* was proposed. The activation of the *Sox11a*–*Dmrt1*–*Sox9b* cascade promotes the male sex differentiation and gonadal development, ultimately leading to testes formation. Conversely, an antagonistic *Foxl2*/*Cyp19a1a* cascade initiates the activation of downstream genes contributing to the development of female ovaries (Fig. 12). Nevertheless, functional studies are imperative to validate the fundamental mechanism underlying sex determination and differentiation in *C. maculata*.

The onset of meiosis in germ cells signals the commencement of molecular sex differentiation in many teleosts [16]. Therefore, detecting the expression of meiotic marker genes can assist in determining the time window for molecular sex differentiation in teleost. *Sycp3* [63, 64] and *Spo11* [65, 66] are highly suitable molecular markers for identifying the entry of spermatogonia or oogonia into meiosis. In *D. rerio*, the knockout of *Sycp3* leads to meiosis cessation, followed by the gradual apoptosis of germ cells, ultimately resulting in complete male sterility [63]. In black porgy (*Acanthopagrus schlegelii*), *Sycp3* is crucial for meiotic competence, with its expression levels being down-regulated following estrogen treatment, thereby affecting germ cell meiosis [64]. The Spo11 antibody has demonstrated the capability to detect pre-meiotic germ cells in Japanese eel (*Anguilla japonica*) [65]. Additionally, male homozygous mutants of *Spo11*^*−/−*^ in *D. rerio* exhibit complete sterility [66]. In our study, the expression of *Sycp3* and *Spo11* sharply increased in females from 30 to 60 dpf, coinciding with the first observation of primary oocytes at 40 dpf (Fig. 2G). This suggests that the time window for molecular ovarian differentiation is between 40 and 60 dpf. In male gonads, *Sycp3* and *Spo11* were significantly up-regulated at 60–90 dpf (Fig. 7B), aligning with the first observation of secondary spermatocytes at 90 dpf (Fig. 2j). Therefore, the crucial time window for molecular testicular differentiation in *C. maculata* is between 60 and 90 dpf. These results reaffirmed that testes mature later than ovaries in *C. maculata*, and provide theoretical support for sexual dimorphism in body sizes and growth rates.

Furthermore, it holds significant importance to evaluate the expression patterns of sex-differentiation-related genes in elucidating the mechanism of gonadal maintenance and gametogenesis in teleost. As a member of the TGF-β superfamily, *Bmp15* plays a pivotal role in follicle formation and granulosa cell proliferation [67]. Notably, targeted mutations of *Bmp15* in female zebrafish result in abnormal *Cyp19a1a* expression in granulosa cells, leading to sex reversal during the middle to late juvenile stages [68]. Our research indicated *Bmp15* expression in females increased substantially after 30 dpf, with negligible expression observed prior to this period (Fig. 10A). This suggests that *Bmp15* is crucial for ovarian differentiation and maintenance in females. Furthermore, *Figla*, an oocyte-specific transcription factor, plays a critical role in oocyte growth and development [29]. In *O.latipes*, XX *Figla*^*–/–*^ mutants fail to form follicles, and the expression of female-specific genes (*Gdf9* and *Bmp15*) is reduced [69]. The destruction of *Figla* in *D. rerio* blocks the transition from cystic CN oocytes to individual follicular perinucleolar oocytes, resulting in all-male phenotype in homozygous mutant [70]. In this study, *Figla* expression gradually rose from 30 dpf and reached its peak at 180 dpf (Fig. 10F), similar to that in *O. niloticus* [71], *P. olivaceus* [72], suggesting that *Figla* plays an important role in ovarian development and oogenesis in *C. maculata*. *Gsdf*, as a member of the TGF-β superfamily, has been demonstrated to be a downstream gene of the sex-determining gene *Dmy* in *O. latipes*, playing a pivotal role in initiating the male sex differentiation pathway [73]. In *O. niloticus*, knockout of *Gsdf* results in the production of XY females [74]. Furthermore, over-expression of *Gsdf* in female medaka also induces the generation of fertile XX males [73]. These findings underscore the critical role of *Gsdf* in testicular differentiation. In this study, *Gsdf* was almost not expressed in females, whereas its expression significantly increased in male snakeheads after 30 dpf (Fig. 9F), suggesting that *Gsdf* is indispensable for testicular differentiation and maintenance in *C. maculata*. *Cyp17a2*, a member of the cytochrome P450 superfamily, plays an integral role in the synthesis of C18, C19, and C21 steroids in head kidney and gonads [75]. In *O. latipes*, homozygous mutation of *Cyp17a2*^*−/−*^ results in reduced secretion of progesterone and cortisol, leading to a decrease in sperm motility and infertility in males, and progesterone can induce spermatogenesis and regulate sperm motility [76]. In this study, *Cyp17a2* exhibited significant male-biased expression from 30 to 180 dpf (Fig. 9D), suggesting that *Cyp17a2* plays a crucial role in spermatogenesis in *C. maculata* through the regulation of progesterone synthesis.

This study has significantly identified numerous biological pathways linked to sex differentiation and gonadal development, providing valuable insights for future research. In males, the main signaling pathways encompassed male sex differentiation, male gonadal development, and male gamete generation, indicating their potential crucial roles in male sex differentiation and spermatogenesis. In females, the identified signaling pathways included the development of primary female sexual characteristics, female gonad development, as well as the canonical Wnt and GnRH signaling pathways. *Foxl2*, *Cyp19a1a*, *Bmp15* and *Figla* are considered critical for ovarian differentiation and maintenance, suggesting their significant roles in female gonadal development in *C. maculata*.

## Conclusion

In summary, this study integrates histological observations, spatiotemporal comparative transcriptome analyses, and sex steroid hormone assays to provide a comprehensive overview of morphological dynamics, sexually dimorphic gene expression patterns, and sex steroid synthesis during sex differentiation and gametogenesis in male and female *C. maculata*. Male-biased genes (*Sox11a*, *Dmrt1*, *Amh*, *Amhr2*, *Gsdf*, *Ar*, *Cyp17a2*) may play crucial roles in male sex differentiation and spermatogenesis. Meanwhile, female-biased genes (*Foxl2*, *Cyp19a1a*, *Bmp15*, *Figla*, *Er*) could be pivotal in ovarian differentiation and development. It is speculated that in *C. maculata*, the potential male sex differentiation pathway, *Sox11a*–*Dmrt1*–*Sox9b*, activates downstream sex-related genes (*Amh*, *Amhr2*, *Gsdf*, *Ar*, *Cyp17a2*) for testicular development, ultimately leading to the formation of testes. Conversely, the antagonistic pathway, *Foxl2/Cyp19a1a*, activates downstream sex-related genes (*Bmp15*, *Figla*, *Er*) involved female ovarian development. Furthermore, this study identified 20–25 dpf or earlier as the morphological ovarian differentiation period and 30–35 dpf or earlier as the morphological testicular differentiation period. Therefore, it was inferred that the period preceding 30 dpf might be the critical time for sex control in *C. maculata.* Additionally, the periods of 40–60 dpf and 60–90 dpf mark the initiation of molecular sex differentiation in females and males, respectively. Moreover, the sex steroid hormones E_2_ and MT exhibit sexual dimorphism during gonadal development. These findings offer critical insights into the mechanism underlying ovarian and testicular development in *Channidae* family. Furthermore, these research outcomes hold significant importance for advancing all-male breeding of *C. maculata* in production practices.

## Perspectives and significance

Blotched snakehead exhibits pronounced sexual dimorphism in growth, leading to significant disparities in market prices between the sexes. Therefore, the cultivation of all-male populations of snakeheads holds substantial economic and ecological value. In our previous study, XY sex-reversal females, YY super-males and XY all-males of *C. maculata* were successfully generated. This breakthrough not only substantially enhances the economic yield but also provides an invaluable model for the investigation of mechanism underlying sex determination and differentiation in fish. However, current research on the mechanism of sex determination and differentiation in *C. maculata* is not comprehensive, with the potential pathways leading from bipotential gonads to either testis or ovary development yet to be accurately clarified. This study provides a comprehensive overview of gonadal morphological changes in *C. maculata*, identifies candidate genes and pathways involved in sex differentiation and gametogenesis, and determines the critical periods for sex differentiation from different levels. Future efforts aim to precisely locate the sex-determining gene by integrating the results of differential gene expression analysis during key periods of sex differentiation, along with GWAS to locate the sex-determining region and gene. Subsequently, molecular biological techniques such as CRISPR/Cas9 and gene over-expression will be used to identify the function of potential sex-determining gene, and produce high-quality monosex germplasm in *C. maculata*. These findings will offer a scientific foundation for the production practices of sex control in *C. maculata*, provide a theoretical basis for a comprehensive analysis of the sex determination and differentiation mechanism in this species, and enrich the content of fish sex chromosome evolution.

## Supplementary Information

Supplementary Material 1: Fig. S1. Global transcriptome profiles of gonads in *C. maculata*. (A) PCA score plots of the first two principal components for 40 gonadal samples. The ovaries, testes and undifferentiated gonads are shown with pink, blue and yellow backgrounds, respectively, (B) Correlation heatmap of ovaries at 90 dpf, (C) Correlation heatmap of 40 gonadal samples.

Supplementary Material 2: Fig. S2. Volcano plots of DEGs in males compared to the corresponding females at ten developmental stages. (A) 10 dpf, (B) 15 dpf, (C) 20 dpf, (D) 25 dpf, (E) 30 dpf, (F) 60 dpf, (G) 90 dpf, (H) 120 dpf, (I) 150 dpf, (J) 180 dpf. Down: down-regulated DEG; Up: up-regulated DEG; Normal: undifferentially expressed gene.

Supplementary Material 3: Fig. S3. Number of DEGs in males compared to the corresponding females at ten developmental stages. Up: up-regulated DEG. down: down-regulated DEG.

Supplementary Material 4: Fig. S4. GO enrichment of DEGs in males compared to the corresponding females at 10 dpf (A, up-regulated; B, down-regulated), 15 dpf (D, up-regulated; E, down-regulated), 20 dpf (G, up-regulated; H, down-regulated), 25 dpf (J, up-regulated; K, down-regulated) and 30 dpf (M, up-regulated; N, down-regulated); KEGG enrichment of DEGs in males compared to the corresponding females at 10 dpf (C), 15 dpf (F), 20 dpf (I), 25 dpf (L) and 30 dpf (O).

Supplementary Material 5: Fig. S5. GO enrichment of DEGs in males compared to the corresponding females at 60 dpf (A, up-regulated; B, down-regulated), 90 dpf (D, up-regulated; E, down-regulated), 120 dpf (G, up-regulated; H, down-regulated), 150 dpf (J, up-regulated; K, down-regulated) and 180dpf (M, up-regulated; N, down-regulated); KEGG enrichment of DEGs in males compared to the corresponding females at 60 dpf (C), 90 dpf (F), 120 dpf (I), 150 dpf (L) and 180 dpf (O).

Supplementary Material 6: Fig. S6. Number of DEGs of pairwise comparisons across nine sample groups (10, 15, 20, 25, 60, 90, 120, 150, and 180 dpf) relative to 30 dpf benchmark in females (A) and males (B). Up: up-regulated DEG. down: down-regulated DEG.

Supplementary Material 7: Fig. S7. Venn diagram illustrates 81 overlapping genes between 900 overlapping DEGs in females and 173 in males (A), Heatmap illustrates the expression levels of these 81 overlapping genes in testes and ovaries across ten development stages (B), GO (C) and KEGG enrichment (D) of these 81 overlapping genes.

Supplementary Material 8: Fig. S8. Chromosomal location of sex-related genes in *C. maculata*.

Supplementary Material 9: Table S1. List of GO terms enriched from DEGs in males compared to the corresponding females at 10, 15, 20, 25, 30 dpf. Table S2. List of GO terms enriched from DEGs in males compared to the corresponding females at 60, 90, 120, 150, 180 dpf. Table S3. List of KEGG pathway enriched from DEGS in males compared with corresponding females at 10, 15, 20, 25, 30 dpf. Table S4. List of KEGG pathway enriched from DEGS in males compared with corresponding females at 60, 90, 120, 150, 180 dpf. Table S5. List of sex-related DEGs in males compared with corresponding females at 10, 15, 20, 25, 30 dpf. Table S6. List of sex-related DEGs in males compared with corresponding females at 60, 90, 120, 150, 180 dpf. Table S7. List of GO terms enriched from 165 sex-related DEGs. Table S8. List of KEGG pathways enriched from 165 sex-related DEGs. Table S9. List of GO terms enriched from the female profile DEGs. Table S10. List of KEGG pathways enriched from the female profile DEGs. Table S11. List of GO terms enriched from the male profile DEGs. Table S12. List of KEGG pathways enriched from the male profile DEGs.

Supplementary Material 10: Original data for length and weight comparisons between XX and XY fish presented in Figure 1.

## Data Availability

All the data related with this project is available with the corresponding author and will be provided. No datasets were generated or analysed during the current study.

## Abbreviations

dpf: Days post-fertilization
E2: 17β-Estradiol
T: Testosterone
*Sry*: Sex-determining region of Y chromosom
*Dmrt1*: Doublesex and mab-3 related transcription factor 1
*Dmrtb1*: Dmrt-like family B with proline-rich C-terminal 1
*Dmy/Dmrt1b*^*Y*^: DM-domain gene on the Y chromosome
*Gsdf*^*Y*^: Gonadal soma derived growth factor on the Y chromosome
*Sox3*^*Y*^: SRY-related HMG-box 3 on the Y chromosome
*Amhr2*: Anti-Müllerian hormone receptor type II
*Sd*^*Y*^: Sexually dimorphic on the Y chromosome
*Amh*^*Y*^: Anti-Müllerian hormone type II receptor Y-linked
*Gdf6*^*Y*^: Growth differentiation factor 6 on the Y chromosome
DEGs: Differentially expressed genes
PGCs: Primordial germ cells
*Sox3*: Sry-box transcription factor 3
*Sox8*: Sry-box transcription factor 8
*Sox9b*: Sry-box transcription factor 9b
*Sox11a*: Sry-box transcription factor 11a
*Sox11b*: Sry-box transcription factor 11b
*Sox15*: Sry-box transcription factor 15
*Sox17*: Sry-box transcription factor 17
*Amh*: Anti-müllerian hormone
*Foxl2*: Forkhead box l2
*Wnt4*: Wingless-type mmtv integration site family member 4
*Figla*: Factor in the germline alpha
*Cyp7b1*: Cytochrome P450 family 7 subfamily b member 1
*Cyp19a1a*: Cytochrome p450 family 19 subfamily a member 1a
*Er*: Estrogen receptor
*Cyp17a1*: Cytochrome p450 family 17 subfamily a member 1
*Cyp17a2*: Cytochrome p450 family 17 subfamily a member 2
*Cyp11a1*: Cytochrome p450 family 11 subfamily a member 1
*Zp4*: Zona pellucida glycoprotein 4
*Zp3*: Zona pellucida glycoprotein 4
*Ar*: Androgen receptor
*Bmp15*: Bone morphogenetic protein 15
*Ctnd2*: Catenin delta 2
Pax4: Paired box 4
*Ccnb1*: Cyclin B1
*Ccnb2*: Cyclin B2
*Cdk1*: Cyclin-dependent kinase 1
*Dmc1*: DNA meiotic recombinase 1
*Hsd11b1*: 11-Beta-hydroxysteroid dehydrogenase 1
*Hsd17b1*: 17-Beta-hydroxysteroid dehydrogenase 1
*Hsd17b12*: 17-Beta-hydroxysteroid dehydrogenase 12
*Sf1*: Steroidogenic factor 1
*Ccnd1*: Cyclin D1
*Nrob1*: Nuclear receptor subfamily o group B member 1
*Bcl2a*: B-cell lymphoma 2a
*Wt1*: Wilms tumor 1
*Sycp1*: Synaptonemal complex protein 1
*Sycp2*: Synaptonemal complex protein 2
*Sycp3*: Synaptonemal complex protein 3*S*
*Ddx4*: Dead-box helicase 4
*Tfap2c*: Transcription factor AP-2 gamma
*Nanog*: Nanog homeobox
*Sall4*: Sal-like 4
*Id4*: Inhibitor of DNA binding 4, HLH Protein
*Spo11*: Spo11 meiotic protein covalently bound to DSB
*Zar1*: Zygote arrest 1
*Nobox*: NOBOX oogenesis homeobox
*Dazl*: Deleted in azoospermia like
*Lhx8*: LIM homeobox 8
*Gnrh3*: Gonadotropin-releasing hormone 3
*Aff1*: Af4/fmr2 family member 1
*Pgrmc1/2*: Progesterone receptor membrane component 1/2
*Akr1d1*: Aldo–keto reductase family 1 member d1
*Ugt1a1*: UDP glucuronosyltransferase family 1 member A1
*Ugt2a3*: UDP glucuronosyltransferase family 2 member A3
*Sult1a4*: Sulfotransferase family 1a member 4
*Sts*: Steroid sulfatase
*Tet1*: Ten-eleven translocation methylcytosine dioxygenase 1
*Gfar1*: Glutathione-dependent formaldehyde-activating enzyme
*Spag6*: Sperm associated antigen 6
