# Supplementary figures and images for: Histological observations and transcriptome analyses reveal the dynamic changes in the gonads of the blotched snakehead (Channa maculata) during sex differentiation and gametogenesis
Source: Biol Sex Differ. 2024 Sep 7;15:70. doi: 10.1186/s13293-024-00643-x (PMC11380785; doi:10.1186/s13293-024-00643-x)

A

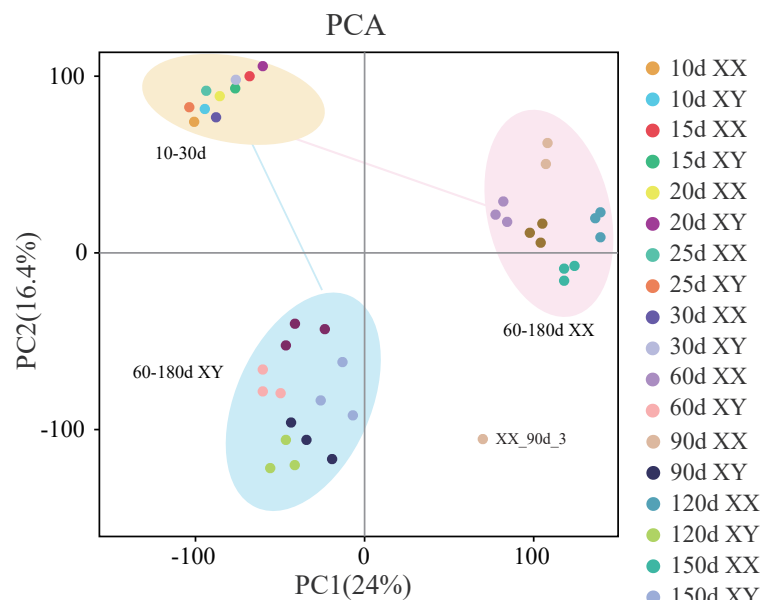

B

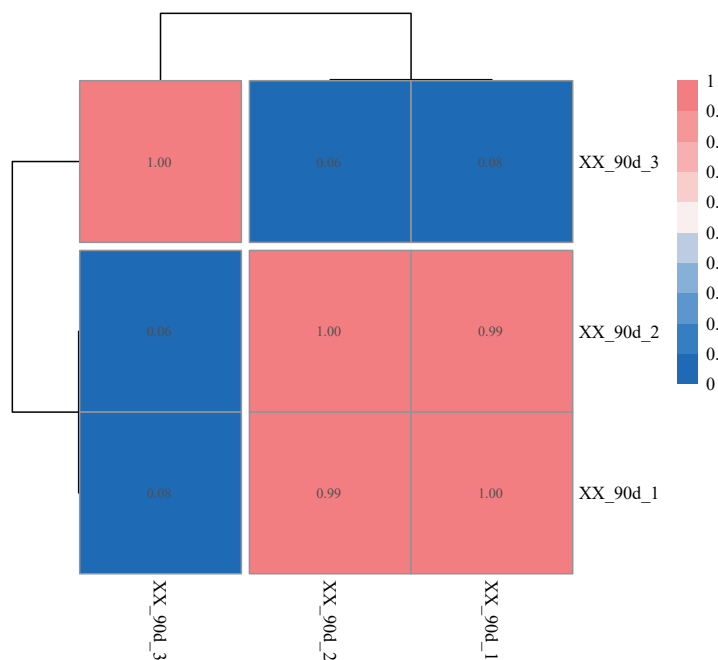

C

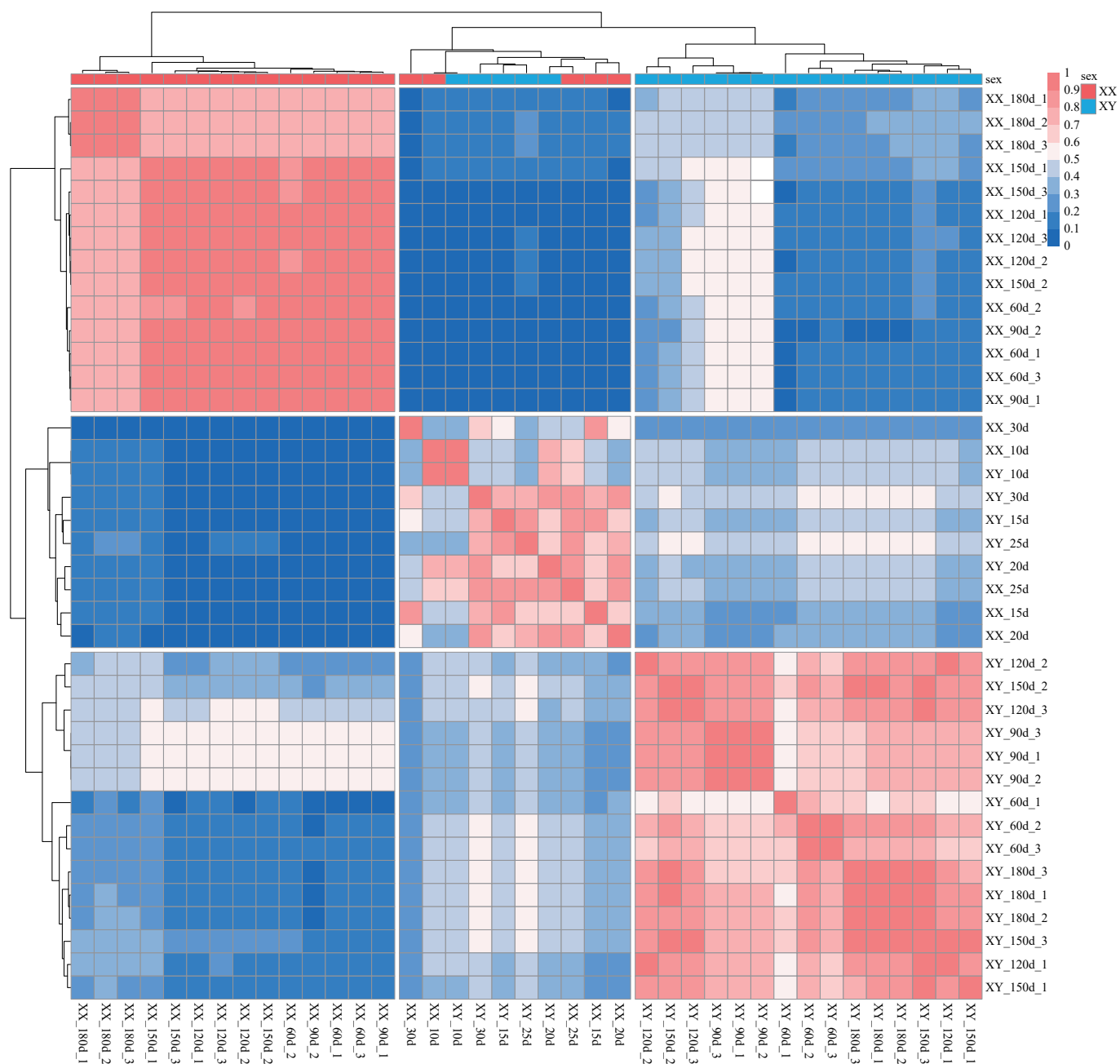

Supplement: Supplementary file 1 — Supplementary Material 1: Fig. S1. Global transcriptome profiles of gonads in C. maculata. (A) PCA score plots of the first two principal components for 40 gonadal samples. The ovaries, testes and undifferentiated gonads are shown with pink, blue and yellow backgrounds, respectively, (B) Correlation heatmap of ovaries at 90 dpf, (C) Correlation heatmap of 40 gonadal samples. [file 13293_2024_643_MOESM1_ESM.pdf]

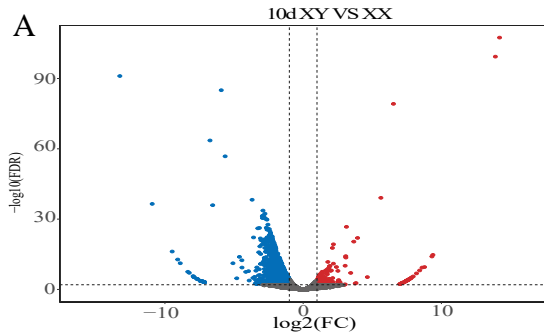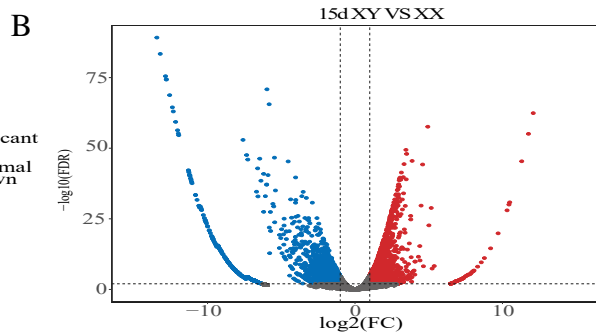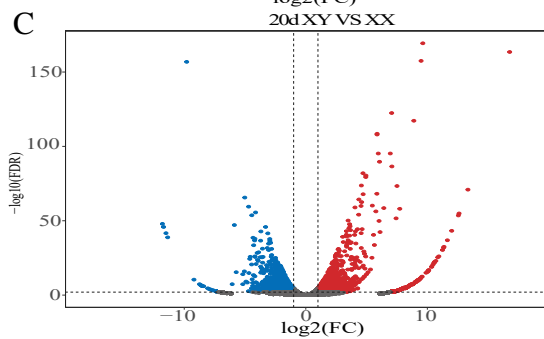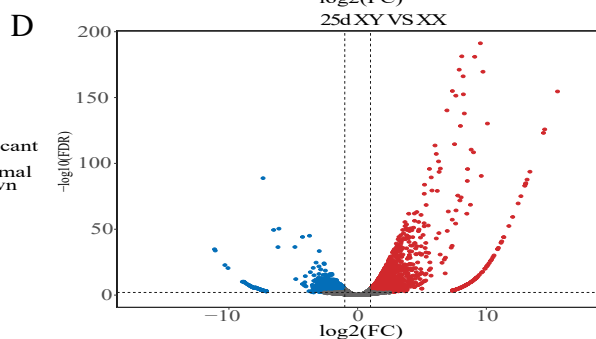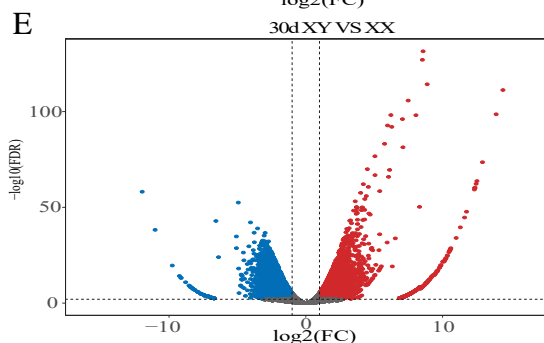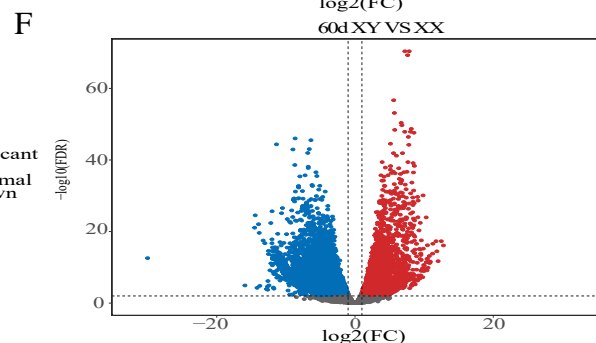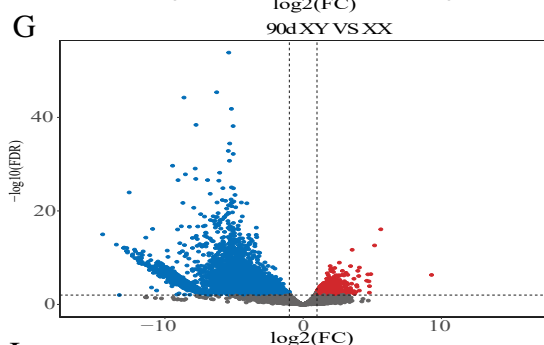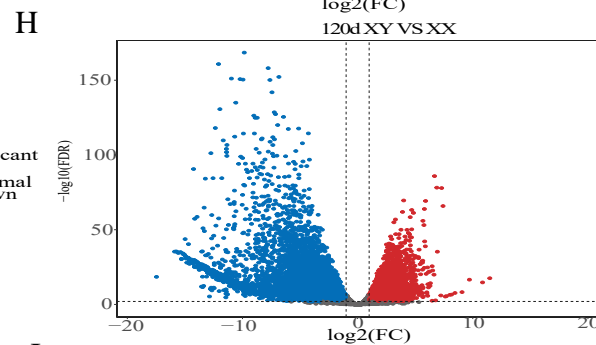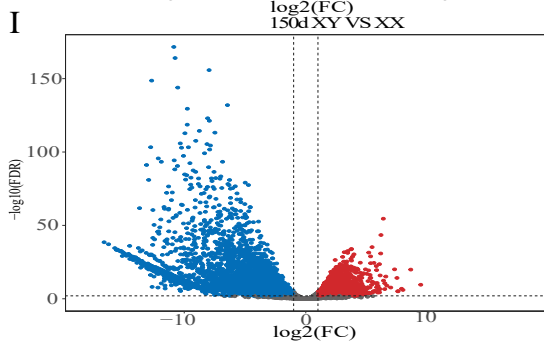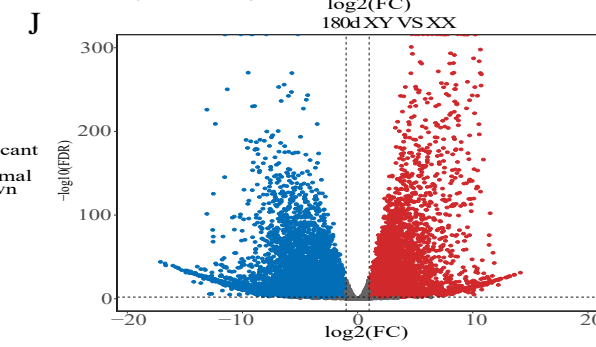

Supplement: Supplementary file 2 — Supplementary Material 2: Fig. S2. Volcano plots of DEGs in males compared to the corresponding females at ten developmental stages. (A) 10 dpf, (B) 15 dpf, (C) 20 dpf, (D) 25 dpf, (E) 30 dpf, (F) 60 dpf, (G) 90 dpf, (H) 120 dpf, (I) 150 dpf, (J) 180 dpf. Down: down-regulated DEG; Up: up-regulated DEG; Normal: undifferentially expressed gene. [file 13293_2024_643_MOESM2_ESM.pdf]

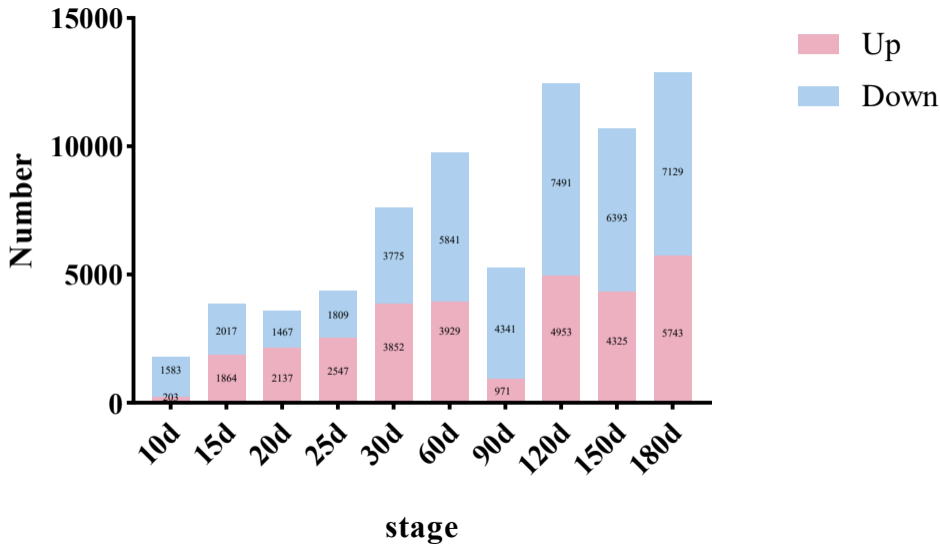

Supplement: Supplementary file 3 — Supplementary Material 3: Fig. S3. Number of DEGs in males compared to the corresponding females at ten developmental stages. Up: up-regulated DEG. down: down-regulated DEG. [file 13293_2024_643_MOESM3_ESM.pdf]

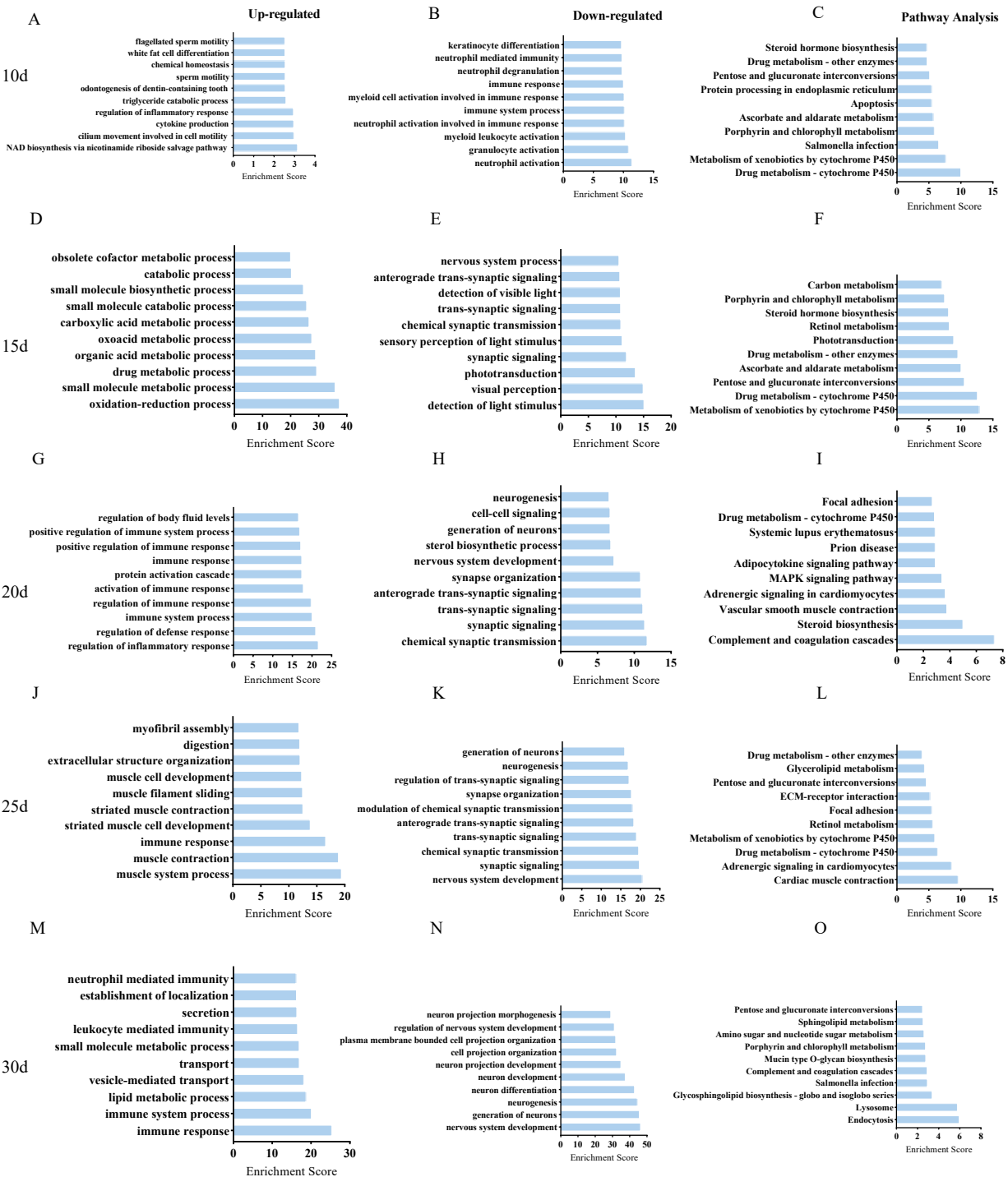

Supplement: Supplementary file 4 — Supplementary Material 4: Fig. S4. GO enrichment of DEGs in males compared to the corresponding females at 10 dpf (A, up-regulated; B, down-regulated), 15 dpf (D, up-regulated; E, down-regulated), 20 dpf (G, up-regulated; H, down-regulated), 25 dpf (J, up-regulated; K, down-regulated) and 30 dpf (M, up-regulated; N, down-regulated); KEGG enrichment of DEGs in males compared to the corresponding females at 10 dpf (C), 15 dpf (F), 20 dpf (I), 25 dpf (L) and 30 dpf (O). [file 13293_2024_643_MOESM4_ESM.pdf]

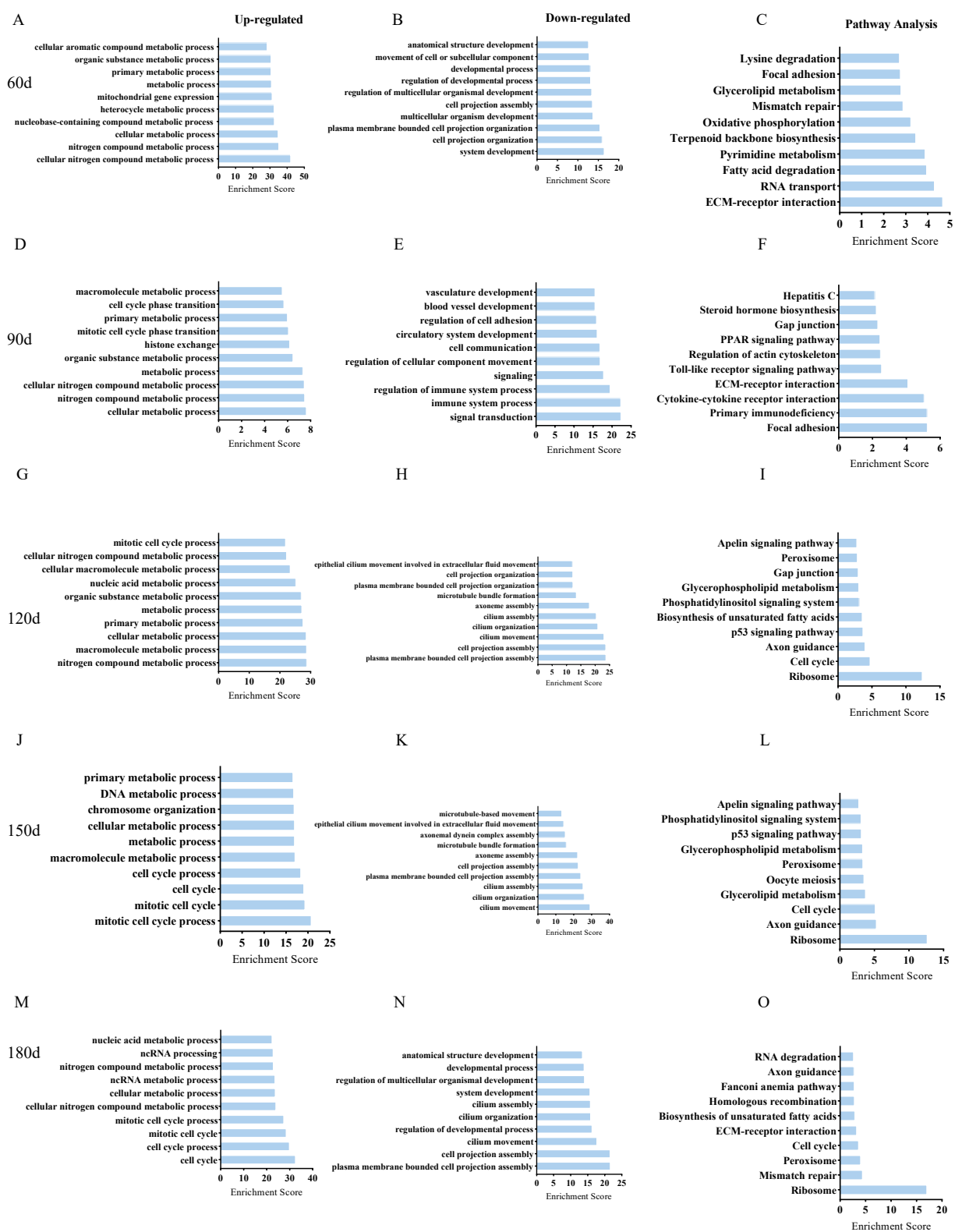

Supplement: Supplementary file 5 — Supplementary Material 5: Fig. S5. GO enrichment of DEGs in males compared to the corresponding females at 60 dpf (A, up-regulated; B, down-regulated), 90 dpf (D, up-regulated; E, down-regulated), 120 dpf (G, up-regulated; H, down-regulated), 150 dpf (J, up-regulated; K, down-regulated) and 180dpf (M, up-regulated; N, down-regulated); KEGG enrichment of DEGs in males compared to the corresponding females at 60 dpf (C), 90 dpf (F), 120 dpf (I), 150 dpf (L) and 180 dpf (O). [file 13293_2024_643_MOESM5_ESM.pdf]

A

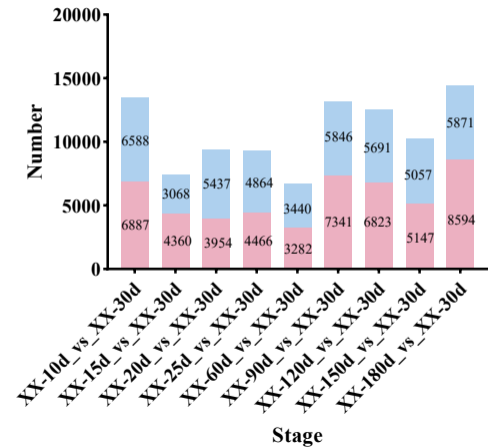

B

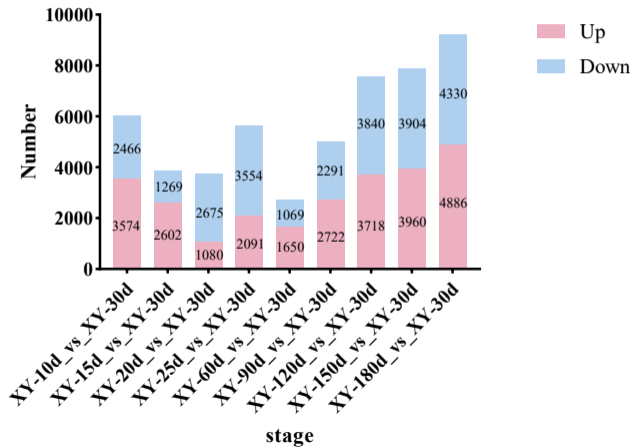

Supplement: Supplementary file 6 — Supplementary Material 6: Fig. S6. Number of DEGs of pairwise comparisons across nine sample groups (10, 15, 20, 25, 60, 90, 120, 150, and 180 dpf) relative to 30 dpf benchmark in females (A) and males (B). Up: up-regulated DEG. down: down-regulated DEG. [file 13293_2024_643_MOESM6_ESM.pdf]

A

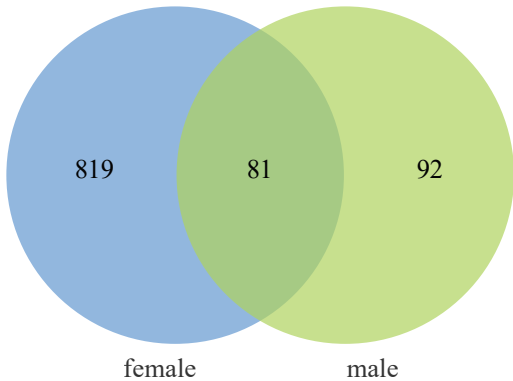

B

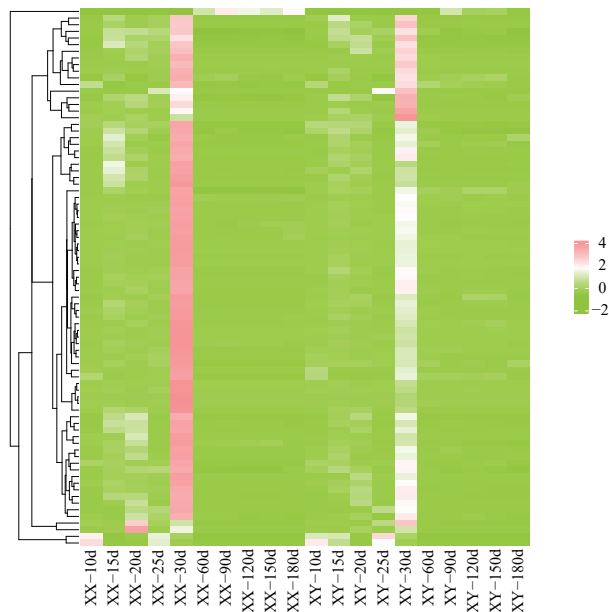

C

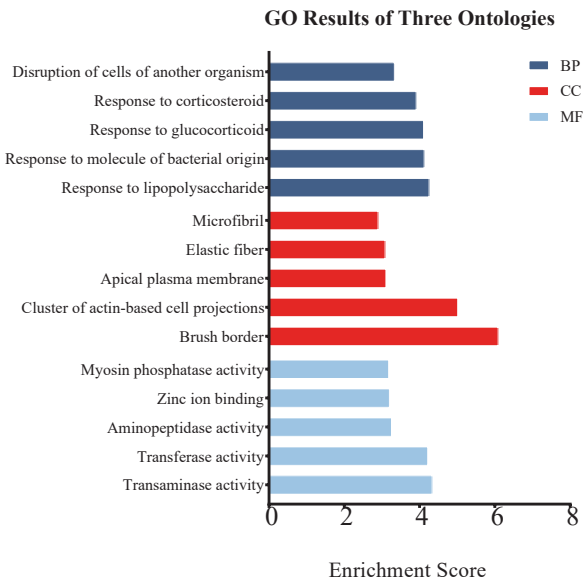

D

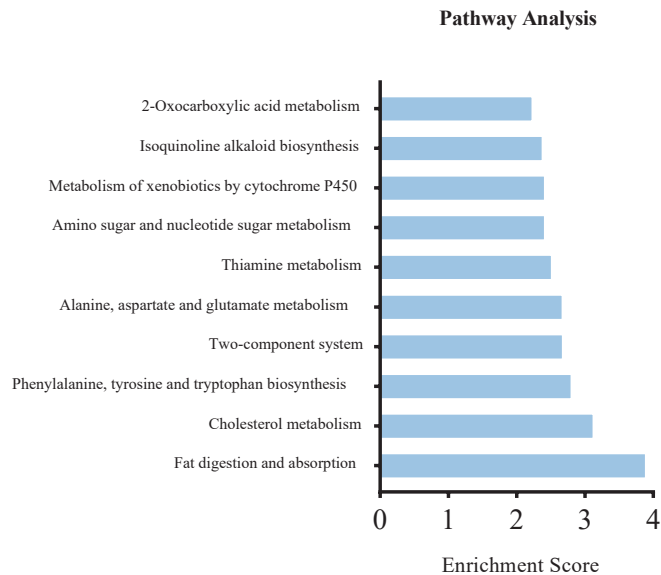

Supplement: Supplementary file 7 — Supplementary Material 7: Fig. S7. Venn diagram illustrates 81 overlapping genes between 900 overlapping DEGs in females and 173 in males (A), Heatmap illustrates the expression levels of these 81 overlapping genes in testes and ovaries across ten development stages (B), GO (C) and KEGG enrichment (D) of these 81 overlapping genes. [file 13293_2024_643_MOESM7_ESM.pdf]

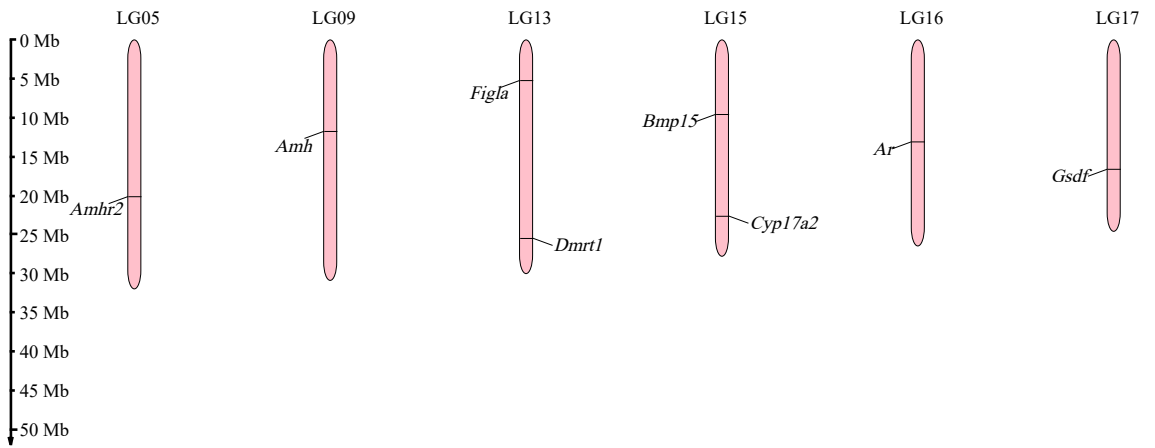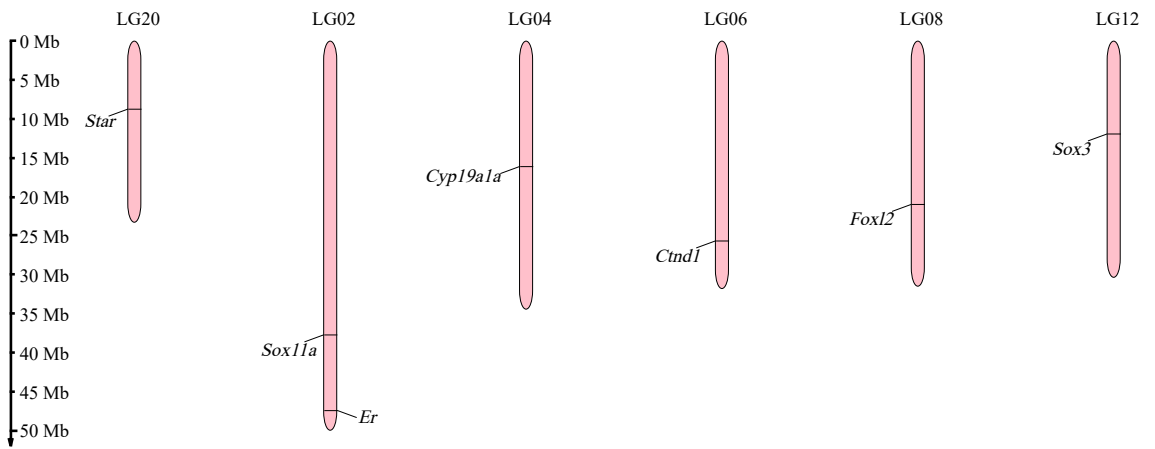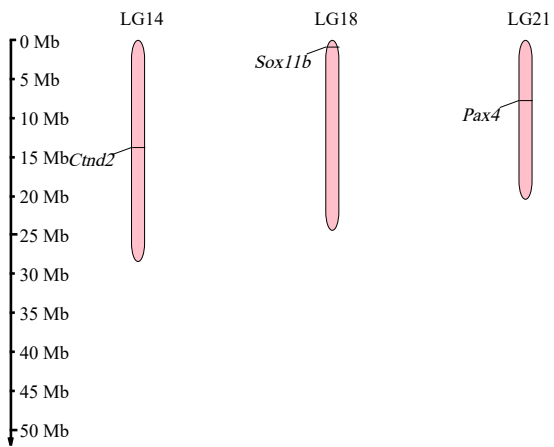

Supplement: Supplementary file 8 — Supplementary Material 8: Fig. S8. Chromosomal location of sex-related genes in C. maculata. [file 13293_2024_643_MOESM8_ESM.pdf]
