# Supplementary material for: Histological observations and transcriptome analyses reveal the dynamic changes in the gonads of the blotched snakehead (Channa maculata) during sex differentiation and gametogenesis
Source: Biol Sex Differ. 2024 Sep 7;15:70. doi: 10.1186/s13293-024-00643-x (PMC11380785; doi:10.1186/s13293-024-00643-x)
Supplement: Supplementary file 9 — Supplementary Material 9: Table S1. List of GO terms enriched from DEGs in males compared to the corresponding females at 10, 15, 20, 25, 30 dpf. Table S2. List of GO terms enriched from DEGs in males compared to the corresponding females at 60, 90, 120, 150, 180 dpf. Table S3. List of KEGG pathway enriched from DEGS in males compared with corresponding females at 10, 15, 20, 25, 30 dpf. Table S4. List of KEGG pathway enriched from DEGS in males compared with corresponding females at 60, 90, 120, 150, 180 dpf. Table S5. List of sex-related DEGs in males compared with corresponding females at 10, 15, 20, 25, 30 dpf. Table S6. List of sex-related DEGs in males compared with corresponding females at 60, 90, 120, 150, 180 dpf. Table S7. List of GO terms enriched from 165 sex-related DEGs. Table S8. List of KEGG pathways enriched from 165 sex-related DEGs. Table S9. List of GO terms enriched from the female profile DEGs. Table S10. List of KEGG pathways enriched from the female profile DEGs. Table S11. List of GO terms enriched from the male profile DEGs. Table S12. List of KEGG pathways enriched from the male profile DEGs. [file 13293_2024_643_MOESM9_ESM.docx]

**Supplementary Tables**

**Table S1** List of GO terms enriched from DEGs in males compared to the corresponding females at 10, 15, 20, 25, 30 dpf.

| **Stage** | **GO ID** | **GO term** | **-Log10(p-value)** | **Gene number** | **Male vs. Female**  **Up/Down** |
| --- | --- | --- | --- | --- | --- |
| 10 dpf | GO:0034356 | NAD biosynthesis via nicotinamide riboside salvage pathway | 3.159837875 | 2 | up |
|  | GO:0060294 | cilium movement involved in cell motility | 2.978283461 | 2 | up |
|  | GO:0001816 | cytokine production | 2.970944705 | 4 | up |
|  | GO:0050727 | regulation of inflammatory response | 2.960954394 | 6 | up |
|  | GO:0019433 | triglyceride catabolic process | 2.592615043 | 2 | up |
|  | GO:0042475 | Odontogenesis of dentin-containing tooth | 2.554926484 | 3 | up |
|  | GO:0097722 | sperm motility | 2.554926484 | 3 | up |
|  | GO:0048878 | chemical homeostasis | 2.553905322 | 11 | up |
|  | GO:0050872 | white fat cell differentiation | 2.542712428 | 2 | up |
|  | GO:0030317 | flagellated sperm motility | 2.535454061 | 3 | up |
|  | GO:0042119 | neutrophil activation | 11.43667003 | 57 | down |
|  | GO:0036230 | granulocyte activation | 10.87679238 | 56 | down |
|  | GO:0002274 | myeloid leukocyte activation | 10.38954228 | 60 | down |
|  | GO:0002283 | neutrophil activation involved in immune response | 10.19332146 | 54 | down |
|  | GO:0002376 | immune system process | 10.19058066 | 175 | down |
|  | GO:0002275 | myeloid cell activation involved in immune response | 10.16907027 | 56 | down |
|  | GO:0006955 | immune response | 10.0545352 | 117 | down |
|  | GO:0043312 | neutrophil degranulation | 9.805881551 | 53 | down |
|  | GO:0002446 | neutrophil mediated immunity | 9.795787066 | 54 | down |
|  | GO:0030216 | keratinocyte differentiation | 9.709105373 | 22 | down |
| 15 dpf | GO:0055114 | oxidation-reduction process | 37.48834971 | 164 | up |
|  | GO:0044281 | small molecule metabolic process | 35.96821349 | 236 | up |
|  | GO:0017144 | drug metabolic process | 29.37658436 | 116 | up |
|  | GO:0006082 | organic acid metabolic process | 28.98545648 | 149 | up |
|  | GO:0043436 | oxoacid metabolic process | 27.63151032 | 145 | up |
|  | GO:0019752 | carboxylic acid metabolic process | 26.66977301 | 135 | up |
|  | GO:0044282 | small molecule catabolic process | 25.82539785 | 83 | up |
|  | GO:0044283 | small molecule biosynthetic process | 24.6831668 | 104 | up |
|  | GO:0009056 | catabolic process | 20.40275722 | 193 | up |
|  | GO:0051186 | obsolete cofactor metabolic process | 20.08883444 | 85 | up |
|  | GO:0009583 | detection of light stimulus | 15.14283201 | 34 | down |
|  | GO:0007601 | visual perception | 14.99695009 | 44 | down |
|  | GO:0007602 | phototransduction | 13.57653067 | 25 | down |
|  | GO:0099536 | synaptic signaling | 11.95339042 | 79 | down |
|  | GO:0050953 | sensory perception of light stimulus | 11.17665147 | 37 | down |
|  | GO:0007268 | chemical synaptic transmission | 10.94881451 | 77 | down |
|  | GO:0099537 | trans-synaptic signaling | 10.89996076 | 76 | down |
|  | GO:0009584 | detection of visible light | 10.84966665 | 25 | down |
|  | GO:0098916 | anterograde trans-synaptic signaling | 10.75774106 | 75 | down |
|  | GO:0050877 | nervous system process | 10.6031345 | 130 | down |
| 20 dpf | GO:0050727 | regulation of inflammatory response | 21.6664123 | 74 | up |
|  | GO:0031347 | regulation of defense response | 21.01239139 | 112 | up |
|  | GO:0002376 | immune system process | 20.09136008 | 251 | up |
|  | GO:0050776 | regulation of immune response | 19.85303715 | 120 | up |
|  | GO:0002253 | activation of immune response | 17.86542565 | 68 | up |
|  | GO:0072376 | protein activation cascade | 17.46059651 | 26 | up |
|  | GO:0006955 | immune response | 17.45270507 | 165 | up |
|  | GO:0050778 | positive regulation of immune response | 17.18928036 | 88 | up |
|  | GO:0002684 | positive regulation of immune system process | 16.94185752 | 119 | up |
|  | GO:0050878 | regulation of body fluid levels | 16.65047501 | 83 | up |
|  | GO:0007268 | chemical synaptic transmission | 11.78907069 | 67 | down |
|  | GO:0099536 | synaptic signaling | 11.44591862 | 66 | down |
|  | GO:0099537 | trans-synaptic signaling | 11.18964577 | 65 | down |
|  | GO:0098916 | anterograde trans-synaptic signaling | 10.97620225 | 64 | down |
|  | GO:0050808 | synapse organization | 10.87117349 | 55 | down |
|  | GO:0007399 | nervous system development | 7.269059768 | 215 | down |
|  | GO:0016126 | sterol biosynthetic process | 6.836634224 | 13 | down |
|  | GO:0048699 | generation of neurons | 6.748283659 | 168 | down |
|  | GO:0007267 | cell-cell signaling | 6.738530906 | 90 | down |
|  | GO:0022008 | neurogenesis | 6.603860196 | 174 | down |
| 25 dpf | GO:0003012 | muscle system process | 19.45947579 | 78 | up |
|  | GO:0006936 | muscle contraction | 18.93376277 | 68 | up |
|  | GO:0006955 | immune response | 16.64728372 | 177 | up |
|  | GO:0055002 | striated muscle cell development | 13.87854915 | 59 | up |
|  | GO:0006941 | striated muscle contraction | 12.56485957 | 35 | up |
|  | GO:0030049 | muscle filament sliding | 12.51472655 | 19 | up |
|  | GO:0055001 | muscle cell development | 12.345119 | 59 | up |
|  | GO:0043062 | extracellular structure organization | 12.064875 | 71 | up |
|  | GO:0007586 | digestion | 11.99607103 | 31 | up |
|  | GO:0030239 | myofibril assembly | 11.85241222 | 39 | up |
|  | GO:0007399 | nervous system development | 20.56707545 | 318 | down |
|  | GO:0099536 | synaptic signaling | 19.83252239 | 94 | down |
|  | GO:0007268 | chemical synaptic transmission | 19.63166633 | 94 | down |
|  | GO:0099537 | trans-synaptic signaling | 19.08099729 | 92 | down |
|  | GO:0098916 | anterograde trans-synaptic signaling | 18.40153025 | 90 | down |
|  | GO:0050804 | modulation of chemical synaptic transmission | 18.05046111 | 88 | down |
|  | GO:0050808 | synapse organization | 17.77507223 | 77 | down |
|  | GO:0099177 | regulation of trans-synaptic signaling | 17.18445301 | 86 | down |
|  | GO:0022008 | neurogenesis | 16.95068687 | 255 | down |
| 30 dpf | GO:0006955 | immune response | 25.41844403 | 279 | up |
|  | GO:0002376 | immune system process | 20.14953372 | 398 | up |
|  | GO:0006629 | lipid metabolic process | 18.80705937 | 239 | up |
|  | GO:0016192 | vesicle-mediated transport | 18.1791566 | 328 | up |
|  | GO:0006810 | transport | 17.04256051 | 610 | up |
|  | GO:0044281 | small molecule metabolic process | 17.00347024 | 324 | up |
|  | GO:0002443 | leukocyte mediated immunity | 16.59024262 | 129 | up |
|  | GO:0046903 | secretion | 16.4268706 | 225 | up |
|  | GO:0051234 | establishment of localization | 16.31912322 | 630 | up |
|  | GO:0002446 | neutrophil mediated immunity | 16.26597623 | 111 | up |
|  | GO:0007399 | nervous system development | 46.41321487 | 656 | down |
|  | GO:0048699 | generation of neurons | 45.73430087 | 529 | down |
|  | GO:0022008 | neurogenesis | 44.64826816 | 545 | down |
|  | GO:0030182 | neuron differentiation | 42.91837266 | 414 | down |
|  | GO:0048666 | neuron development | 37.58615565 | 355 | down |
|  | GO:0031175 | neuron projection development | 35.02546453 | 306 | down |
|  | GO:0030030 | cell projection organization | 32.50536732 | 382 | down |
|  | GO:0120036 | plasma membrane bounded cell projection organization | 32.03616004 | 378 | down |
|  | GO:0051960 | regulation of nervous system development | 31.24217834 | 324 | down |

**Table S2** List of GO terms enriched from DEGs in males compared to the corresponding females at 60, 90, 120, 150, 180 dpf.

| **Stage** | **GO ID** | **GO term** | **-Log10(p-value)** | **Gene number** | **Male vs. Female**  **Up/Down** |
| --- | --- | --- | --- | --- | --- |
| 60 dpf | GO:0034641 | cellular nitrogen compound metabolic process | 42.19513673 | 698 | up |
|  | GO:0006807 | nitrogen compound metabolic process | 35.25651555 | 1108 | up |
|  | GO:0044237 | cellular metabolic process | 34.89807709 | 1183 | up |
|  | GO:0006139 | nucleobase-containing compound metabolic process | 32.67556824 | 586 | up |
|  | GO:0046483 | heterocycle metabolic process | 32.54904308 | 613 | up |
|  | GO:0140053 | mitochondrial gene expression | 31.38906501 | 72 | up |
|  | GO:0008152 | metabolic process | 31.01960342 | 1224 | up |
|  | GO:0044238 | primary metabolic process | 30.77172446 | 1136 | up |
|  | GO:0071704 | organic substance metabolic process | 30.76876104 | 1181 | up |
|  | GO:0006725 | cellular aromatic compound metabolic process | 28.4821056 | 611 | up |
|  | GO:0048731 | system development | 16.49954577 | 1211 | down |
|  | GO:0030030 | cell projection organization | 15.97545197 | 463 | down |
|  | GO:0120036 | plasma membrane bounded cell projection organization | 15.46197999 | 457 | down |
|  | GO:0007275 | multicellular organism development | 13.74963221 | 1344 | down |
|  | GO:0030031 | cell projection assembly | 13.60666759 | 167 | down |
|  | GO:2000026 | regulation of multicellular organismal development | 13.44989036 | 632 | down |
|  | GO:0050793 | regulation of developmental process | 13.17143274 | 754 | down |
|  | GO:0032502 | developmental process | 13.11438916 | 1446 | down |
|  | GO:0006928 | movement of cell or subcellular component | 12.79710999 | 485 | down |
|  | GO:0048856 | anatomical structure development | 12.66685883 | 1401 | down |
| 90 dpf | GO:0044237 | cellular metabolic process | 7.648886564 | 296 | up |
|  | GO:0006807 | nitrogen compound metabolic process | 7.507160754 | 276 | up |
|  | GO:0034641 | cellular nitrogen compound metabolic process | 7.473279299 | 167 | up |
|  | GO:0008152 | metabolic process | 7.342288283 | 309 | up |
|  | GO:0071704 | organic substance metabolic process | 6.490949222 | 294 | up |
|  | GO:0043486 | histone exchange | 6.196142236 | 11 | up |
|  | GO:0044772 | mitotic cell cycle phase transition | 6.099419025 | 26 | up |
|  | GO:0044238 | primary metabolic process | 6.006166603 | 280 | up |
|  | GO:0044770 | cell cycle phase transition | 5.698943143 | 26 | up |
|  | GO:0043170 | macromolecule metabolic process | 5.555620996 | 234 | up |
|  | GO:0007165 | signal transduction | 22.40171428 | 745 | down |
|  | GO:0002376 | immune system process | 22.35580769 | 429 | down |
|  | GO:0002682 | regulation of immune system process | 19.55582632 | 279 | down |
|  | GO:0023052 | signaling | 17.84350849 | 754 | down |
|  | GO:0051270 | regulation of cellular component movement | 16.94035495 | 244 | down |
|  | GO:0007154 | cell communication | 16.84573883 | 773 | down |
|  | GO:0072359 | circulatory system development | 16.13792963 | 233 | down |
|  | GO:0030155 | regulation of cell adhesion | 15.98431575 | 166 | down |
|  | GO:0001568 | blood vessel development | 15.59660844 | 132 | down |
|  | GO:0001944 | vasculature development | 15.5944617 | 137 | down |
| 120 dpf | GO:0006807 | nitrogen compound metabolic process | 28.81350849 | 1373 | up |
|  | GO:0043170 | macromolecule metabolic process | 28.7764757 | 1208 | up |
|  | GO:0044237 | cellular metabolic process | 28.63353134 | 1472 | up |
|  | GO:0044238 | primary metabolic process | 27.59316208 | 1428 | up |
|  | GO:0008152 | metabolic process | 27.21303088 | 1540 | up |
|  | GO:0071704 | organic substance metabolic process | 27.0160904 | 1484 | up |
|  | GO:0090304 | nucleic acid metabolic process | 25.2526897 | 565 | up |
|  | GO:0044260 | cellular macromolecule metabolic process | 23.44083681 | 1027 | up |
|  | GO:0034641 | cellular nitrogen compound metabolic process | 22.23825483 | 785 | up |
|  | GO:1903047 | mitotic cell cycle process | 21.92242801 | 229 | up |
|  | GO:0120031 | plasma membrane bounded cell projection assembly | 23.82584535 | 208 | down |
|  | GO:0030031 | cell projection assembly | 23.77233485 | 215 | down |
|  | GO:0003341 | cilium movement | 23.14156143 | 62 | down |
|  | GO:0044782 | cilium organization | 20.980103 | 170 | down |
|  | GO:0060271 | cilium assembly | 20.46637872 | 167 | down |
|  | GO:0035082 | axoneme assembly | 18.04283863 | 57 | down |
|  | GO:0001578 | microtubule bundle formation | 13.48369685 | 70 | down |
|  | GO:0120036 | plasma membrane bounded cell projection organization | 12.29521628 | 511 | down |
|  | GO:0030030 | cell projection organization | 12.24926535 | 515 | down |
|  | GO:0003351 | epithelial cilium movement involved in extracellular fluid movement | 12.14240229 | 31 | down |
| 150 dpf | GO:1903047 | mitotic cell cycle process | 20.80392056 | 207 | up |
|  | GO:0000278 | mitotic cell cycle | 19.3834351 | 227 | up |
|  | GO:0007049 | cell cycle | 19.11017986 | 300 | up |
|  | GO:0022402 | cell cycle process | 18.42310208 | 277 | up |
|  | GO:0043170 | macromolecule metabolic process | 17.11040628 | 1021 | up |
|  | GO:0008152 | metabolic process | 16.99893961 | 1317 | up |
|  | GO:0044237 | cellular metabolic process | 16.97811582 | 1251 | up |
|  | GO:0051276 | chromosome organization | 16.91980282 | 233 | up |
|  | GO:0006259 | DNA metabolic process | 16.799033 | 173 | up |
|  | GO:0044238 | primary metabolic process | 16.63725869 | 1215 | up |
|  | GO:0003341 | cilium movement | 29.20209688 | 63 | down |
|  | GO:0044782 | cilium organization | 25.99393893 | 161 | down |
|  | GO:0060271 | cilium assembly | 25.30190767 | 158 | down |
|  | GO:0120031 | plasma membrane bounded cell projection assembly | 24.0414636 | 185 | down |
|  | GO:0030031 | cell projection assembly | 22.63210613 | 188 | down |
|  | GO:0035082 | axoneme assembly | 22.31449221 | 57 | down |
|  | GO:0001578 | microtubule bundle formation | 15.97399415 | 67 | down |
|  | GO:0070286 | axonemal dynein complex assembly | 15.39330614 | 30 | down |
|  | GO:0003351 | epithelial cilium movement involved in extracellular fluid movement | 14.55851783 | 31 | down |
| 180 dpf | GO:0007049 | cell cycle | 32.60518009 | 409 | up |
|  | GO:0022402 | cell cycle process | 29.98232371 | 374 | up |
|  | GO:0000278 | mitotic cell cycle | 28.5983117 | 300 | up |
|  | GO:1903047 | mitotic cell cycle process | 27.50320485 | 266 | up |
|  | GO:0034641 | cellular nitrogen compound metabolic process | 24.08156964 | 891 | up |
|  | GO:0044237 | cellular metabolic process | 23.84392266 | 1638 | up |
|  | GO:0034660 | ncRNA metabolic process | 23.70595723 | 183 | up |
|  | GO:0006807 | nitrogen compound metabolic process | 23.00772863 | 1519 | up |
|  | GO:0034470 | ncRNA processing | 22.89992708 | 132 | up |
|  | GO:0090304 | nucleic acid metabolic process | 22.4446584 | 619 | up |
|  | GO:0120031 | plasma membrane bounded cell projection assembly | 21.67534918 | 205 | down |
|  | GO:0030031 | cell projection assembly | 21.63291178 | 212 | down |
|  | GO:0003341 | cilium movement | 17.73205099 | 57 | down |
|  | GO:0050793 | regulation of developmental process | 16.33620188 | 909 | down |
|  | GO:0044782 | cilium organization | 15.9040418 | 160 | down |
|  | GO:0060271 | cilium assembly | 15.84418981 | 158 | down |
|  | GO:0048731 | system development | 15.7056988 | 1429 | down |
|  | GO:2000026 | regulation of multicellular organismal development | 14.10070254 | 747 | down |
|  | GO:0032502 | developmental process | 13.99400352 | 1728 | down |

**Table S3** List of KEGG pathway enriched from DEGS in males compared with corresponding females at 10, 15, 20, 25, 30 dpf.

| **Stage** | **Term** | **Pathway** | **Gene number** | **-Log10(p-value)** |
| --- | --- | --- | --- | --- |
| XY10d vs. XX10d | ko00982 | Drug metabolism - cytochrome P450 | 22 | 10.03219 |
|  | ko00980 | Metabolism of xenobiotics by cytochrome P450 | 19 | 7.664356 |
|  | ko05132 | Salmonella infection | 56 | 6.564105 |
|  | ko00860 | Porphyrin and chlorophyll metabolism | 16 | 5.888253 |
|  | ko00053 | Ascorbate and aldarate metabolism | 13 | 5.722658 |
|  | ko04210 | Apoptosis | 41 | 5.4769 |
|  | ko04141 | Protein processing in endoplasmic reticulum | 43 | 5.466795 |
|  | ko00040 | Pentose and glucuronate interconversions | 14 | 5.148763 |
|  | ko00983 | Drug metabolism - other enzymes | 20 | 4.761351 |
|  | ko00140 | Steroid hormone biosynthesis | 17 | 4.710675 |
| XY15d vs. XX15d | ko00980 | Metabolism of xenobiotics by cytochrome P450 | 33 | 12.92081 |
|  | ko00982 | Drug metabolism - cytochrome P450 | 33 | 12.62289 |
|  | ko00040 | Pentose and glucuronate interconversions | 27 | 10.5706 |
|  | ko00053 | Ascorbate and aldarate metabolism | 23 | 10.06368 |
|  | ko00983 | Drug metabolism - other enzymes | 39 | 9.578743 |
|  | ko04744 | Phototransduction | 26 | 8.916622 |
|  | ko00830 | Retinol metabolism | 36 | 8.226891 |
|  | ko00140 | Steroid hormone biosynthesis | 31 | 8.11619 |
|  | ko00860 | Porphyrin and chlorophyll metabolism | 25 | 7.485456 |
|  | ko01200 | Carbon metabolism | 54 | 7.026553 |
| XY20d vs. XX20d | ko04610 | Complement and coagulation cascades | 12 | 7.381396 |
|  | ko00100 | Steroid biosynthesis | 13 | 5.015565 |
|  | ko04270 | Vascular smooth muscle contraction | 48 | 3.788362 |
|  | ko04261 | Adrenergic signaling in cardiomyocytes | 57 | 3.672181 |
|  | ko04010 | MAPK signaling pathway | 93 | 3.417702 |
|  | ko04920 | Adipocytokine signaling pathway | 27 | 2.919445 |
|  | ko05020 | Prion disease | 7 | 2.913346 |
|  | ko05322 | Systemic lupus erythematosus | 7 | 2.913346 |
|  | ko00982 | Drug metabolism - cytochrome P450 | 17 | 2.862688 |
|  | ko04510 | Focal adhesion | 67 | 2.689756 |
| XY25d vs. XX25d | ko04260 | Cardiac muscle contraction | 57 | 9.62155 |
|  | ko04261 | Adrenergic signaling in cardiomyocytes | 82 | 8.595035 |
|  | ko00982 | Drug metabolism - cytochrome P450 | 26 | 6.407628 |
|  | ko00980 | Metabolism of xenobiotics by cytochrome P450 | 25 | 5.965825 |
|  | ko00830 | Retinol metabolism | 33 | 5.637347 |
|  | ko04510 | Focal adhesion | 91 | 5.484963 |
|  | ko04512 | ECM-receptor interaction | 47 | 5.218151 |
|  | ko00040 | Pentose and glucuronate interconversions | 20 | 4.654286 |
|  | ko00561 | Glycerolipid metabolism | 30 | 4.362018 |
|  | ko00983 | Drug metabolism - other enzymes | 30 | 3.956757 |
| XY30d vs. XX30d | ko04144 | Endocytosis | 180 | 5.964647239 |
|  | ko04142 | Lysosome | 96 | 5.799264069 |
|  | ko00603 | Glycosphingolipid biosynthesis - globo and isoglobo series | 14 | 3.40023994 |
|  | ko05132 | Salmonella infection | 132 | 2.951425859 |
|  | ko04610 | Complement and coagulation cascades | 11 | 2.927660042 |
|  | ko00512 | Mucin type O-glycan biosynthesis | 21 | 2.803079641 |
|  | ko00860 | Porphyrin and chlorophyll metabolism | 26 | 2.773308302 |
|  | ko00520 | Amino sugar and nucleotide sugar metabolism | 36 | 2.633785079 |
|  | ko00600 | Sphingolipid metabolism | 35 | 2.547947427 |
|  | ko00040 | Pentose and glucuronate interconversions | 23 | 2.496864367 |

**Table S4** List of KEGG pathway enriched from DEGS in males compared with corresponding females at 60, 90, 120, 150, 180 dpf.

| **Stage** | **Term** | **Pathway** | **Gene number** | **-Log10(p-value)** |
| --- | --- | --- | --- | --- |
| XY60d vs. XX60d | ko04512 | ECM-receptor interaction | 80 | 4.689703429 |
|  | ko03013 | RNA transport | 100 | 4.316626054 |
|  | ko00071 | Fatty acid degradation | 33 | 3.958376814 |
|  | ko00240 | Pyrimidine metabolism | 47 | 3.888284656 |
|  | ko00900 | Terpenoid backbone biosynthesis | 19 | 3.467317209 |
|  | ko00190 | Oxidative phosphorylation | 74 | 3.244088649 |
|  | ko03430 | Mismatch repair | 17 | 2.88677741 |
|  | ko00561 | Glycerolipid metabolism | 46 | 2.792379099 |
|  | ko04510 | Focal adhesion | 154 | 2.765926957 |
|  | ko00310 | Lysine degradation | 51 | 2.725664039 |
| XY90d vs. XX90d | ko04510 | Focal adhesion | 106 | 5.261515201 |
|  | ko05340 | Primary immunodeficiency | 14 | 5.248153337 |
|  | ko04060 | Cytokine-cytokine receptor interaction | 100 | 5.070433553 |
|  | ko04512 | ECM-receptor interaction | 51 | 4.107589661 |
|  | ko04620 | Toll-like receptor signaling pathway | 47 | 2.554044361 |
|  | ko04810 | Regulation of actin cytoskeleton | 91 | 2.485788324 |
|  | ko03320 | PPAR signaling pathway | 32 | 2.45685292 |
|  | ko04540 | Gap junction | 48 | 2.326511844 |
|  | ko00140 | Steroid hormone biosynthesis | 24 | 2.242669209 |
|  | ko05160 | Hepatitis C | 6 | 2.151769685 |
| XY120d vs. XX120d | ko03010 | Ribosome | 101 | 12.42648561 |
|  | ko04110 | Cell cycle | 110 | 4.757033365 |
|  | ko04360 | Axon guidance | 45 | 4.012030941 |
|  | ko04115 | p53 signaling pathway | 75 | 3.710580463 |
|  | ko01040 | Biosynthesis of unsaturated fatty acids | 31 | 3.599534759 |
|  | ko04070 | Phosphatidylinositol signaling system | 85 | 3.177611714 |
|  | ko00564 | Glycerophospholipid metabolism | 79 | 3.085904905 |
|  | ko04540 | Gap junction | 94 | 3.015623477 |
|  | ko04146 | Peroxisome | 70 | 2.883934807 |
|  | ko04371 | Apelin signaling pathway | 119 | 2.800453636 |
| XY150d vs. XX150d | ko03010 | Ribosome | 92 | 12.66499891 |
|  | ko04360 | Axon guidance | 43 | 5.310892062 |
|  | ko04110 | Cell cycle | 98 | 5.16379334 |
|  | ko00561 | Glycerolipid metabolism | 50 | 3.739754455 |
|  | ko04114 | Oocyte meiosis | 84 | 3.520351413 |
|  | ko04146 | Peroxisome | 63 | 3.361245831 |
|  | ko00564 | Glycerophospholipid metabolism | 70 | 3.322315063 |
|  | ko04115 | p53 signaling pathway | 64 | 3.140187413 |
|  | ko04070 | Phosphatidylinositol signaling system | 74 | 3.101983533 |
|  | ko04371 | Apelin signaling pathway | 103 | 2.773967217 |
| XY180d vs. XX180d | ko03010 | Ribosome | 112 | 17.06952188 |
|  | ko03430 | Mismatch repair | 22 | 4.448902584 |
|  | ko04146 | Peroxisome | 78 | 4.08701045 |
|  | ko04110 | Cell cycle | 112 | 3.676286691 |
|  | ko04512 | ECM-receptor interaction | 93 | 3.285023479 |
|  | ko01040 | Biosynthesis of unsaturated fatty acids | 31 | 2.929781055 |
|  | ko03440 | Homologous recombination | 32 | 2.844180474 |
|  | ko03460 | Fanconi anemia pathway | 51 | 2.834732294 |
|  | ko04360 | Axon guidance | 44 | 2.7695674 |
|  | ko03018 | RNA degradation | 60 | 2.68250669 |

**Table S5** List of sex-related DEGs in males compared with corresponding females at 10, 15, 20, 25, 30 dpf.

| **Stage** | **Gene name** | **Gene full name** | **Function** | **Male vs. Female**  **Up/down** |
| --- | --- | --- | --- | --- |
| 10 dpf | *Acta2* | Actin alpha 2 | Smooth muscle cell function; cell migration and proliferation | down |
|  | *Npm1* | Nucleophosmin 1 | regulation of cell growth and division | down |
|  | *Actn4* | Alpha-actinin-4 | retinoic acid receptor signaling pathway | down |
|  | *Calr* | Calreticulin | cell adhesion; migration and proliferation | down |
|  | *Myh11* | Myosin heavy chain 11 | smooth muscle cell function; cell migration and proliferation | down |
|  | *Myl6* | Myosin light chain 6 | regulates cell growth, differentiation and migration | down |
|  | *Serinc2* | Serine incorporator 2 | cell proliferation and migration | down |
|  | *Casp3* | Caspase-3 | response to estradiol | down |
|  | *Hsd17b12* | Hydroxysteroid (17-beta) dehydrogenase 12 | regulation of steroid biosynthetic process | down |
|  | *Anxa1* | Annexin a1 | response to estradiol | down |
|  | *Nrob1* | Nuclear receptor subfamily 0 group b member 1 | male sex determination; spermatogenesis; Sertoli cell differentiation; male gonad development | up |
|  | *Sycp1* | Synaptonemal complex protein 1 | meiosis of germ cells; pairing and crossing over of homologous chromosomes | down |
|  | *Er* | Estrogen receptor | response to estradiol; development of the ovaries | down |
|  | *Sdc4* | Syndecan 4 | cell adhesion; migratino signalig | down |
|  | *Bcl2* | Bcl-2-like protein 11 | male gonad development; spermatogenesis | down |
|  | *Bap31* | B-cell receptor-associated protein | spermatogenesis | down |
|  | *Anc* | Angiotensinogen | Ovarian follicle rupture; response to estradiol | up |
| 15 dpf | *Lbh* | Protein lbh | negative regulation of intracellular estrogen receptor signaling pathway | down |
|  | *Tfap2c* | Transcription factor ap-2 gamma | embryonic development; cell proliferation and differentiation | down |
|  | *Lrp2* | Low-density lipoprotein receptorrelated protein 2 | male gonad development | down |
|  | *Gata4* | Gata binding protein 4 | gonadal development and maturation | up |
|  | *Emx2* | Empty spiracles homeobox 2 | formation of the reproductive ridge | down |
|  | *Gfra1* | Gdnf family receptor alpha 1 | regulation of male germ cell survival and differentiation | down |
|  | *Ctnnd2* | Catenin delta 2 | cell adhesion and signal transduction | down |
|  | *Wdr19* | Wd repeat-containing protein 19 | gonad development; in utero embryonic development | down |
|  | *Er* | Estrogen receptor | response to estradiol; development of the ovaries | up |
|  | *Sdc4* | Syndecan 4 | cell adhesion migration signaling | up |
|  | *Star* | Steroidogenic acute regulatory protein | mitochondrial response to estrogen; regulation of steroid biosynthetic process; male gonad development; response to steroid hormone; response to gonadotropin | up |
|  | *Hsd17b12* | Hydroxysteroid (17-beta) dehydrogenase 12 | regulation of steroid biosynthetic process | up |
|  | *Dmrt2* | Double sex- and mab-3-related | sex differentiation | up |
|  | *Hsd17b12a* | Hydroxysteroid 17-beta dehydrogenase 12a | regulation of steroid biosynthetic process | down |
|  | *Hsd3b1* | 3β-hydroxysteroid dehydrogenase type 1 | steroid hormone biosynthesis | up |
|  | *Igf2* | Insulin-like growth factor ii | response to estradiol; female pregnancy | up |
|  | *Piwil1* | P-element induced wimpy testis like protein 1 | play a key role in the development and maintenance of germ cells | down |
| 20 dpf | *Star* | Steroidogenic acute regulatory protein | mitochondrial response to estrogen; regulation of steroid biosynthetic process; male gonad development; response to steroid hormone; response to gonadotropin | up |
|  | *Txnip* | Thioredoxin-interacting protein | response to estradiol; response to progesterone | up |
|  | *Hspa5* | Heat shock protein family a (hsp70) member 5 | regulation of apoptosis | up |
|  | *Hsd3b1* | 3β-hydroxysteroid dehydrogenase type 1 | steroid hormone biosynthesis; ketone body metabolism | up |
|  | *Wt1* | Wilms tumor 1 | gonadal development | up |
|  | *Nrob1* | Nuclear receptor subfamily 0 group b member 1 | male sex determination; spermatogenesis; Sertoli cell differentiation; male gonad development | up |
|  | *Hsd17b12a* | Hydroxysteroid 17-beta dehydrogenase 12a | regulation of steroid biosynthetic process | down |
|  | *Cpeb1* | Cytoplasmic polyadenylation element binding protein 1 | Oocyte maturation and early embryonic development | down |
|  | *Map1b* | Microtubule-associated protein 1b | Microtubule-associated protein 1B | up |
|  | *Mapk1* | Mitogen-activated protein kinase 1 | response to estrogen | down |
|  | *Ptch1* | Protein patched homolog 1 | response to estradiol | up |
|  | *Piwil1* | P-element induced wimpy testis like protein 1 | play a key role in the development and maintenance of germ cells | down |
| 25 dpf | *Myh11* | Myosin heavy chain 11 | smooth muscle cell function; cell migration and proliferation | up |
|  | *Apob* | Apolipoprotein b-100 | spermatogenesis | up |
|  | *Wt1* | Wilms tumor 1 | gonadal development | up |
|  | *Hsd3b1* | 3β-hydroxysteroid dehydrogenase type 1 | steroid hormone biosynthesis; ketone body metabolism | up |
|  | *Star* | Steroidogenic acute regulatory protein | mitochondrial  response to estrogen; regulation of steroid biosynthetic process; male gonad development; response to steroid hormone; response to gonadotropin | up |
|  | *Tspan33* | Tetraspanin 33 | cell proliferation; migration and differentitation | up |
|  | *Celf4* | Cugbp elav-like family member 4 | cell migration; apoptosis and stress | down |
|  | *Sycp1* | Synaptonemal complex protein 1 | meiosis of germ cells; pairing and crossing over of homologous chromosomes | up |
|  | *Er* | Estrogen receptor | response to estradiol; development of the ovaries | up |
|  | *Amh* | Anti-mullerian hormone | development of primary male sexual characteristics | up |
|  | *Fshr* | Follicle-stimulating hormone receptor | primary ovarian follicle growth; ovulation cycle process; ovarian follicle development; follicle-stimulating hormone signaling pathway; spermatid development; spermatogenesis; spermatogenesis exchange of chromosomal proteins; Sertoli cell development; Sertoli cell proliferation; male gonad development | up |
|  | *Piwil2* | P-element induced wimpy testis like protein 2 | play a key role in the development and maintenance of germ cells | up |
|  | *Wnt4* | Wnt family member 4 | regulation of endothelial and steroidogenic cell migration; female gonda development | down |
|  | *Cyp19a1a* | Aromatase | Female sex differentiation; female gonad development; female sex determination | up |
|  | *Mapk1* | Mitogen-activated protein kinase 1 | response to estrogen | down |
| 30 dpf | *Lhx8* | Lim homeobox 8 | Oocyte differentiation and survival | down |
|  | *Src* | Proto-oncogene tyrosine-protein kinase src | regulation of intracellular estrogen receptor signaling pathway; progesterone receptor signaling pathway | down |
|  | *Egr4* | Early growth response 4 | cell proliferation; differentiation and apoptosis | down |
|  | *Vgf* | Neurosecretory protein vgf | ovarian follicle development | down |
|  | *Ctndd2* | Catenin delta 2 | cell adhesion and signal transduction | down |
|  | *Sox11b* | Sry-box transcription factor 11b | sex differentiation | down |
|  | *Foxo3* | Forkhead box protein o3 | oocyte maturation; ovulation from ovarian follicle; antral ovarian follicle growth | down |
|  | *Podxl2* | Podocalyxin like 2 | cell adhesion and migration | down |
|  |  | Sry-box transcription factor 11a | neural development; tissue reconstruction and cell fate determination | down |
|  | *Sox3* | Transcription factor sox-3 | sex determination; spermatid differentiation; Sertoli cell development | down |
|  | *Sox4* | Transcription factor sox-4 | positive regulation of canonical Wnt signaling pathway | down |
|  | *Myl6* | Myosin light chain 6 | regulates cell growth; differentiation and migration | up |
|  | *Hsd17b12* | Hydroxysteroid (17-beta) dehydrogenase 12 | regulation of steroid biosynthetic process | up |
|  | *Ang* | Angiotensinogen | ovarian follicle rupture; response to estradiol | up |
|  | *Apob* | Apolipoprotein b-100 | Spermatogenesis | up |
|  | *Bcl2* | Bcl-2-like protein 11 | male gonad development; spermatogenesis | up |
|  | *Rspo1* | R-spondin 1 | female gonda development | up |
|  | *Gata4* | Gata binding protein 4 | gonadal development and maturation | up |
|  | *Igf2* | Insulin-like growth factor ii | response to estradiol; female pregnancy | up |
|  | *Calr* | Calreticulin | cell adhesion; migration and proliferation | up |
|  | *Piwil1* | P-element induced wimpy testis like protein 1 | play a key role in the development and maintenance of germ cells | up |

**Table S6** List of sex-related DEGs in males compared with corresponding females at 60, 90, 120, 150, 180 dpf.

| **Stage** | **Gene name** | **Gene full name** | **Function** | **Male vs. Female**  **Up/down** |
| --- | --- | --- | --- | --- |
| 60 dpf | *Cyp17a1* | Cytochrome p450 family 17 subfamily a member 1 | regulation of steroid biosynthetic process | down |
|  | *Cyp17a2* | Cytochrome p450 family 17 subfamily a member 2 | regulation of steroid biosynthetic process | down |
|  | *Inhbb* | Inhibin beta b chain | positive regulation of ovulation; oocyte development; positive regulation of follicle stimulating hormone secretion | down |
|  | *Lrp2* | Low-density lipoprotein receptorrelated protein 2 | male gonad development; | down |
|  | *Bmp7* | Bone morphogenetic protein 7 | response to estradiol | down |
|  | *Star* | Steroidogenic acute regulatory protein | mitochondrial response to estrogen; regulation of steroid biosynthetic process; male gonad development response to steroid hormone; response to gonadotropin | down |
|  | *Wnt2b* | Protein wnt-2b | male gonad development | down |
|  | *Vegfa* | Vascular endothelial growth factor a | ovarian follicle development | down |
|  | *Star2* | Steroidogenic acute regulatory protein | mitochondrial response to estrogen; regulation of steroid biosynthetic process; male gonad development response to steroid hormone; response to gonadotropin | down |
|  | *Pdgfa* | Platelet-derived growth factor receptor alpha | male genitalia development | down |
|  | *Frem2* | Fras1 related extracellular matrix protein 2 | cell adhesion and migration | down |
|  | *Fshr* | Follicle-stimulating hormone receptor | primary ovarian follicle growth; ovulation cycle process; ovarian follicle development; follicle-stimulating hormone signaling pathway; spermatid development; spermatogenesis; spermatogenesis exchange of chromosomal proteins; Sertoli cell development; Sertoli cell proliferation; male gonad development | down |
|  | *Gata4* | Gata binding protein 4 | gonadal development and maturation | down |
|  | *Nr5a1* | Nuclear receptor subfamily 5 group a | formation of the reproductive ridge | down |
|  | *Gsdf* | Gonadal soma-derived factor | sex determination and differentiation | down |
|  | *Sox8* | Sry-box transcription factor 8 | sex determination and differentiation | down |
|  | *Gas2* | Growth arrest-specific protein 2 | initiation of primordial ovarian follicle growth; ovulation; antral ovarian follicle growth | down |
|  | *Celf4* | Cugbp elav-like family member 4 | cell migration, apoptosis and stress | down |
|  | *Hsd3b1* | 3β-hydroxysteroid dehydrogenase type 1 | steroid hormone biosynthesis; ketone body metabolism | down |
|  | *Dmc1* | Meiotic recombination protein dmc1/lim15 homolog | oogenesis; ovarian follicle development; oocyte maturation; female gamete generation | down |
|  | *Lhx8* | Lim homeobox 8 | Oocyte differentiation and survival | down |
|  | *Amh* | Anti-mullerian hormone | development of primary male sexual characteristics | down |
|  | *Tex11* | Testis-expressed protein 11 | male gonad development | down |
|  | *Spata22* | Spermatogenesis associated 22 | meiosis in germ cells | down |
|  | *Rec8* | Meiotic recombination protein rec8 homolog | male meiosis; seminiferous tubule development; spermatid development; spermatogenesis; oocyte maturation; | down |
|  | *Amhr2* | Anti-mullerian hormone receptor type 2 | male sexual differentiation and the regression of female reproductive structures (Mullerian ducts) during fetal development | down |
|  | *Ar* | Androgen receptor | male sexual differentiation and development | down |
|  | *Cd38* | Cyclic adp-ribose hydrolase | cell signaling and calcium signaling | down |
|  | *Nanos2* | Nanos c2hc-type zinc finger 2 | development and maintenance of germ cells | down |
|  | *Dmrtb1* | Doublesex and mab-3 related transcription factor b1 | male gonad development | down |
|  | *Thy1* | Thymocyte differentiation antigen 1 | cell adhesion migration; neuronal cell development and immune regulation | down |
|  | *Ace* | Angiotensin-converting enzyme | spermatogenesis | down |
|  | *Smad6* | Mothers against decapentaplegic homolog 6 | response to estrogen | down |
|  | *Qrich2* | Glutamine rich 2 | morphology and function of the sperm flagellum | down |
|  | *Tgfb1* | Transforming growth factor beta-1 proprotein | response to estradiol; response to progesterone | down |
|  | *Sox9* | Sry-box transcription factor 9 | male gonad development | down |
|  | *Dmrt2* | Double sex- and mab-3-related | sex differentiation | down |
|  | *Inha* | Inhibin alpha chain | ovarian follicle development; negative regulation of follicle-stimulating hormone secretion; male gonad development | down |
|  | *Tet1* | Tet methylcytosine dioxygenase 1 | DNA methylation and demethylation | down |
|  | *Zmiz1* | Zinc finger miz domain-containing protein 1 | vitellogenesis | down |
|  | *Bcl6b* | B-cell lymphoma 6 protein | spermatogenesis | down |
|  | *Adcy3* | Adenylate cyclase type 3 | acrosome reaction; flagellated sperm motility; single fertilization | down |
|  | *Irx5* | Iroquois-class homeodomain protein irx-5 | gonad development | down |
|  | *Gata1* | Erythroid transcription factor | male gonad development | down |
|  | *Ash1l* | Histone-lysine n-methyltransferase ash1l | flagellated sperm motility; uterine gland development | down |
|  | *Dmrt1* | Double sex- and mab-3-related transcription factor 1 | proliferation; Sertoli cell differentiation; spermatogenesis; germ cell migration; sex differentiation; Sertoli cell development; oocyte development; developmental process involved in reproduction | down |
|  | *Inhba* | Inhibin beta a chain | male gonad development; ovarian follicle development | down |
|  | *Wnt4* | Wnt family member 4 | regulation of endothelial and steroidogenic cell migration; female gonda development | down |
|  | *Sycp1* | Synaptonemal complex protein 1 | meiosis of germ cells; pairing and crossing over of homologous chromosomes | down |
|  | *Penk* | Response to estradiol | response to estradiol | down |
|  | *Sh2b2* | Sh2b adaptor protein 2 | lipid metabolism insulin signaling | down |
|  | *Dock8* | Dedicator of cytokinesis 8 | cell proliferation and migration | down |
|  | *Robo2* | Roundabout homolog 2 | gonad development | down |
|  | *Casp3* | Caspase-3 | response to estradiol | down |
|  | *Wt1* | Wilms tumor 1 | gonadal development | down |
|  | *Acsl6* | Acyl-coa synthetase long chain family member 6 | vitellogenesis | down |
|  | *Nrip1* | Nuclear receptor-interacting protein 1 | ovarian follicle rupture; ovulation; cellular response to estradiol stimulus | down |
|  | *Foxo3* | Forkhead box protein o3 | oocyte maturation; ovulation from ovarian follicle; antral ovarian follicle growth | down |
|  | *Rspo1* | R-spondin 1 | female gonda development | down |
|  | *Dvl1* | Dishevelled segment polarity protein 1 | cell fate determination, proliferation and migration | down |
|  | *Sycp2* | Synaptonemal complex protein 2 | female meiotic nuclear division; male meiotic nuclear division; male genitalia morphogenesis | down |
|  | *Kdm2b* | Lysine-specific demethylase 2b | spermatogenesis | down |
|  | *Mmp14* | Matrix metalloproteinase-14 | response to estrogen; ovarian follicle development | down |
|  | *Hormad1* | Horma domain containing 1 | plays a key role in meiosis of germ cells | down |
|  | *Rara* | Retinoic acid receptor alpha | response to estradiol; germ cell development; female pregnancy | down |
|  | *Sox17* | Sry-box transcription factor 17 | embryonic development and blood vessel formation | down |
|  | *Ncor1* | Nuclear receptor corepressor 1 | negative regulation of androgen receptor signaling pathway | down |
|  | *Spo11* | Meiotic recombination protein spo11 | Oogenesis; ovarian follicle development; spermatid development | down |
|  | *Ptch1* | Protein patched homolog 1 | response to estradiol | down |
|  | *Sycp3* | Synaptonemal complex protein 3 | meiosis of germ cells; pairing and crossing over of homologous chromosomes | down |
|  | *Etv4* | Ets variant transcription factor 4 | Proliferation and differentiation of embryonic stem cells | down |
|  | *Txnip* | Thioredoxin-interacting protein | response to estradiol; response to progesterone | down |
|  | *Zmiz1* | Zinc finger miz domain-containing protein 1 | female meiosis chromosome segregation; male meiosis chromosome segregation | down |
|  | *Ube3a* | Ubiquitin-protein ligase e3a | ovarian follicle development; sperm entry; response to progesterone; androgen receptor signaling pathway | down |
|  | *Ctnnd2* | Catenin delta 2 | cell adhesion and signal transduction | up |
|  | *Brd2b* | Bromodomain-containing protein 2b | spermatogenesis | up |
|  | *Dnd1* | Dead end homolog 1 | survival and maintenance of germ cells | up |
|  | *Ang* | Angiotensinogen | ovarian follicle rupture; response to estradiol | up |
|  | *Nsdhl* | Nad(p) dependent steroid dehydrogenase-like | 3-beta-hydroxy-delta5-steroid dehydrogenase activity; 3-beta-hydroxysteroid dehydrogenase/ 3-beta-hydroxy-delta5-steroid dehydrogenase activity; 3-beta-hydroxysteroid dehydrogenase | up |
|  | *Dedd* | Death effector domain-containing protein | spermatogenesis | up |
|  | *Vegfa* | Vascular endothelial growth factor a | ovarian follicle development | up |
|  | *Cbl* | E3 ubiquitin-protein ligase cbl | male gonad development; response to testosterone | up |
|  | *Prdx4* | Peroxiredoxin-4 | male gonad development | up |
|  | *Cnot3* | Ccr4-not transcription complex subunit 3 | negative regulation of intracellular estrogen receptor signaling pathway | up |
|  | *Src* | Src proto-oncogene, non-receptor tyrosine kinase | regulation of intracellular estrogen receptor signaling pathway; progesterone receptor signaling pathway | up |
|  | *Ang* | Angiotensinogen | ovarian follicle rupture; response to estradiol | up |
|  | *Spy1* | Speedy protein a | male meiotic nuclear division | up |
|  | *Cpeb1* | Cytoplasmic polyadenylation element binding protein 1 | Oocyte maturation and early embryonic development | up |
|  | *Sall4* | Spalt-like transcription factor 4 | embryonic development and stem cell pluripotency | up |
|  | *Figla* | Factor in the germline alpha | female gonad development | up |
|  | *Calr* | Calreticulin | cell adhesion migration and proliferation | up |
|  | *Sox11b* | Sry-box transcription factor 11b | sex differentiation | up |
|  | *Sdc4* | Syndecan 4 | Cell adhesion migration and proliferation | up |
|  | *Lbh* | Protein lbh | negative regulation of intracellular estrogen receptor signaling pathway | up |
|  | *Hspa5* | Heat shock protein family a (hsp70) member 5 | regulation of apoptosis | up |
|  | *Foxo3* | Forkhead box protein o3 | oocyte maturation; ovulation from ovarian follicle; antral ovarian follicle growth | up |
|  | *Rgs14* | Regulator of g protein signaling 14 | G protein signaling; cell cycle regulation | up |
|  | *Nanos3* | Nanos c2hc-type zinc finger 3 | development and maintenance of germ cells | up |
|  | *Wee2* | Wee1-like protein kinase 2 | female meiotic nuclear division; female pronucleus assembly; negative regulation of oocyte maturation | up |
|  | *Hsd17b12a* | Hydroxysteroid 17-beta dehydrogenase 12a | regulation of steroid biosynthetic process | up |
|  | *Zglp1* | Gata-type zinc finger protein 1 | spermatogenesis; oocyte development | up |
|  | *Bmp15* | Bone morphogenetic protein 15 | involved in folliculogenesis | up |
|  | *Nanog* | Nanog homeobox | self-renewal and pluripotency of embryonic stem cells | up |
|  | *Sfrp1* | Secreted frizzled-related protein 1 | male gonad development; development of primary male sexual characteristics; female gonad development; cellular response to estradiol stimulus; negative regulation of androgen receptor signaling pathway | up |
|  | *Sox3* | Transcription factor sox-3 | sex determination; spermatid differentiation; Sertoli cell development | up |
|  | *Zp3* | Zona pellucida sperm-binding protein 3 | oocyte development; positive regulation of ovarian follicle development; positive regulation of antral ovarian follicle growth; negative regulation of binding of sperm to zona pellucida | up |
|  | *Tfap2C* | Transcription factor ap-2 gamma | embryonic development; cell proliferation and differentiation | up |
|  | *Zp4* | Zona pellucida sperm-binding protein 4 | negative regulation of binding of sperm to zona pellucida | up |
| 90 dpf | *Gata4* | Gata binding protein 4 | gonadal development and maturation | down |
|  | *Spata22* | Spermatogenesis associated 22 | meiosis in germ cells | down |
|  | *Qrich2* | Glutamine rich 2 | morphology and function of the sperm flagellum | down |
|  | *Cyp11a1* | Cytochrome p450 family 11 subfamily a member 1 | steroid hormone biosynthesis | down |
|  | *Cyp17a1* | Cytochrome p450 family 17 subfamily a member 1 | regulation of steroid biosynthetic process | down |
|  | *Gsdf* | Gonadal soma-derived factor | sex determination and differentiation | down |
|  | *Igf1* | Insulin-like growth factor 1 | regulation of cell growth cell division and metabolism | down |
|  | *Colla2* | Collagen type i alpha 2 chain | Cell proliferation, migration and differentitation | down |
|  | *Dmc1* | Meiotic recombination protein dmc1/lim15 homolog | oogenesis; ovarian follicle development; oocyte maturation; female gamete generation | down |
|  | *Acta2* | Alpha smooth muscle actin (a-smooth muscle actin) | smooth muscle cell function | down |
|  | *Myh11* | Myosin heavy chain 11 | smooth muscle cell function | down |
|  | *Cyp19a1a* | Aromatase | female sex differentiation; female gonad development; female sex determination | up |
|  | *Dmrtb1* | Doublesex and mab-3 related transcription factor b1 | male gonad development | down |
|  | *Ar* | Androgen receptor | male sexual differentiation and development | down |
|  | *Tex11* | Testis-expressed protein 11 | male gonad development | down |
|  | *Inha* | Inhibin alpha chain | ovarian follicle development; negative regulation of follicle-stimulating hormone secretion; male gonad development | down |
|  | *Fshr* | Follicle-stimulating hormone receptor | primary ovarian follicle growth; ovulation cycle process; ovarian follicle development; follicle-stimulating hormone signaling pathway; spermatid development; spermatogenesis, migration and differentitation | down |
|  | *Cyp17a2* | Cytochrome p450 family 17 subfamily a member 2 | regulation of steroid biosynthetic process | down |
|  | *Star* | Steroidogenic acute regulatory protein | mitochondrial response to estrogen; regulation of steroid biosynthetic process; male gonad development response to steroid hormone; response to gonadotropin | down |
|  | *Sycp2* | Synaptonemal complex protein 2 | female meiotic nuclear division; male meiotic nuclear division; male genitalia morphogenesis | down |
|  | *Nr5a1* | Nuclear receptor subfamily 5 group a | formation of the reproductive ridge | down |
|  | *Folr1* | Folate receptor alpha | fusion of sperm to egg plasma membrane involved in single fertilization; sperm-egg recognition | down |
| 120 dpf | *Apob* | Apolipoprotein b-100 | spermatogenesis | down |
|  | *Tex11* | Testis-expressed protein 11 | male gonad development | down |
|  | *Nr5a1* | Nuclear receptor subfamily 5 | formation of the reproductive ridge | down |
|  | *Inhbb* | Inhibin beta b chain | positive regulation of ovulation; oocyte development; positive regulation of follicle stimulating hormone secretion | down |
|  | *Igf1* | Insulin-like growth factor 1 | regulation of cell growth; cell division and metabolism | down |
|  | *Spata22* | Spermatogenesis associated 22 | meiosis in germ cells | down |
|  | *Star* | Steroidogenic acute regulatory protein | mitochondrial response to estrogen; regulation of steroid biosynthetic process; male gonad development response to steroid hormone; response to gonadotropin | down |
|  | *Gsdf* | Gonadal soma-derived factor | sex determination and differentiation | down |
|  | *Dmc1* | Meiotic recombination protein dmc1/lim15 homolog | oogenesis; ovarian follicle development; oocyte maturation; female gamete generation | down |
|  | *Acsl6* | Acyl-coa synthetase long chain family member 6 | vitellogenesis | down |
|  | *Bcl2* | Bcl-2-like protein 11 | male gonad development; spermatogenesis | down |
|  | *Dmrtb1* | Doublesex and mab-3 related transcription factor b1 | male gonad development | down |
|  | *Qrich2* | Glutamine rich 2 | morphology and function of the sperm flagellum | down |
|  | *Ar* | Androgen receptor | male sexual differentiation and development | down |
|  | *Amhr2* | Anti-mullerian hormone receptor type 2 | male sexual differentiation and the regression of female reproductive structures (M眉llerian ducts) during fetal development | down |
|  | *Inha* | Inhibin alpha chain | ovarian follicle development; negative regulation of follicle-stimulating hormone secretion; male gonad development | down |
|  | *Gata1* | Erythroid transcription factor | male gonad development | down |
|  | *Cyp11a1* | Cytochrome p450 family 11 subfamily a member 1 | steroid hormone biosynthesis | down |
|  | *Nanos2* | Nanos c2hc-type zinc finger 2 | development and maintenance of germ cells | down |
|  | *Cyp17a1* | Cytochrome p450 family 17 subfamily a member 1 | regulation of steroid biosynthetic process | down |
|  | *Fshr* | Follicle-stimulating hormone receptor | primary ovarian follicle growth; ovulation cycle process; ovarian follicle development; follicle-stimulating hormone signaling pathway; spermatid development; spermatogenesis; spermatogenesis exchange of chromosomal proteins; Sertoli cell development; Sertoli cell proliferation; male gonad development | down |
|  | *Vegfa* | Vascular endothelial growth factor a | ovarian follicle development | down |
|  | *Gata4* | Gata binding protein 4 | gonadal development and maturation | down |
|  | *Dmrt1* | Double sex- and mab-3-related transcription factor 1 | proliferation; Sertoli cell differentiation; spermatogenesis; germ cell migration; sex differentiation; Sertoli cell development; oocyte development; developmental process involved in reproduction | down |
|  | *Bmp7* | Bone morphogenetic protein 7 | response to estradiol | down |
|  | *Sox9* | Sry-box transcription factor 9 | male gonad development | down |
|  | *Rec8* | Meiotic recombination protein rec8 homolog | male meiosis I; seminiferous tubule development; spermatid development; spermatogenesis; oocyte maturation; | down |
|  | *Celf4* | Cugbp elav-like family member 4 | cell migration, apoptosis and stress | down |
|  | *Gas2* | Growth arrest-specific protein 2 | initiation of primordial ovarian follicle growth; ovulation; antral ovarian follicle growth | down |
|  | *Sycp1* | Synaptonemal complex protein 1 | meiosis of germ cells; pairing and crossing over of homologous chromosomes | down |
|  | *Wnt4* | Wnt family member 4 | regulation of endothelial and steroidogenic cell migration; female gonda development | down |
|  | *Nr5a1* | Nuclear receptor subfamily 5 | formation of the reproductive ridge | down |
|  | *Cyp17a2* | Cytochrome p450 family 17 subfamily a member 2 | regulation of steroid biosynthetic process | down |
|  | *Sh2b2* | Sh2b adaptor protein 2 | lipid metabolism insulin signaling | down |
|  | *Lrp2* | Low-density lipoprotein receptorrelated protein 2 | male gonad development; | down |
|  | *Spag6* | Sperm-associated antigen 6 | spermatogenesis; cell proliferation apoptosis | down |
|  | *Gjb1* | Gap junction beta-1 protein | epididymis development | down |
|  | *Podxl2* | Podocalyxin like 2 | cell adhesion and migration | down |
|  | *Tet1* | Tet methylcytosine dioxygenase 1 | DNA methylation and demethylation | down |
|  | *Nrip1* | Nuclear receptor-interacting protein 1 | ovarian follicle rupture; ovulation; cellular response to estradiol stimulus | down |
|  | *Casp3* | Caspase-3 | response to estradiol | down |
|  | *Hsd3b1* | 3β-hydroxysteroid dehydrogenase type 1 | steroid hormone biosynthesis; ketone body metabolism | down |
|  | *Amh* | Anti-mullerian hormone | development of primary male sexual characteristics | down |
|  | *Star2* | Steroidogenic acute regulatory protein | mitochondrial response to estrogen; regulation of steroid biosynthetic process; male gonad development response to steroid hormone; response to gonadotropin | down |
|  | *Ash1l* | Histone-lysine n-methyltransferase ash1l | flagellated sperm motility; uterine gland development | down |
|  | *Ace* | Angiotensin-converting enzyme | spermatogenesis | down |
|  | *Pim2* | Proviral integration site 2 | cell cycle cell survival and cell proliferation | down |
|  | *Rara* | Retinoic acid receptor alpha | response to estradiol; germ cell development; female pregnancy | down |
|  | *Hormad1* | Horma domain containing 1 | plays a key role in meiosis of germ cells | down |
|  | *Dock8* | Dedicator of cytokinesis 8 | cell proliferation and migration | down |
|  | *Anxa1* | Annexin a1 | response to estradiol | down |
|  | *Bcl6b* | B-cell lymphoma 6 protein | spermatogenesis | down |
|  | *Tgfb1* | Transforming growth factor beta-1 proprotein | response to estradiol; response to progesterone | down |
|  | *Mmp14* | Matrix metalloproteinase-14 | response to estrogen; ovarian follicle development | down |
|  | *Robo2* | Roundabout homolog 2 | gonad development | down |
|  | *Wt1* | Wilms tumor 1 | gonadal development | down |
|  | *Folr1* | Folate receptor alpha | fusion of sperm to egg plasma membrane involved in single fertilization; sperm-egg recognition | down |
|  | *Sycp2* | Synaptonemal complex protein 2 | female meiotic nuclear division; male meiotic nuclear division; male genitalia morphogenesis | down |
|  | *Sycp3* | Synaptonemal complex protein 3 | meiosis of germ cells; pairing and crossing over of homologous chromosomes | down |
|  | *Frem2* | Fras1 related extracellular matrix protein 2 | cell adhesion and migration | down |
|  | *Zmiz1* | Zinc finger miz domain-containing protein 1 | vitellogenesis | down |
|  | *Thy1* | Thymocyte differentiation antigen 1 | cell adhesion migration; neuronal cell development and immune regulation | down |
|  | *Adcy3* | Adenylate cyclase type 3 | acrosome reaction; flagellated sperm motility; single fertilization | down |
|  | *Serinch2* | Serine incorporator 2 | cell proliferation and migration | down |
|  | *Foxo3* | Forkhead box protein o3 | oocyte maturation; ovulation from ovarian follicle; antral ovarian follicle growth | down |
|  | *Dvl1* | Dishevelled segment polarity protein 1 | cell fate determination, proliferation and migration | down |
|  | *Wnt2b* | Protein wnt-2b | male gonad development | down |
|  | *Wdr19* | Wd repeat-containing protein 19 | gonad development; in utero embryonic development | down |
|  | *Pdgfa* | Platelet-derived growth factor receptor alpha | male genitalia development | down |
|  | *Kdm2b* | Lysine-specific demethylase 2b | spermatogenesis | down |
|  | *Usp9x* | Probable ubiquitin carboxyl terminal hydrolase faf-x | female gamete generation; cell migration | down |
|  | *Sox8* | Sry-box transcription factor 8 | sex determination and differentiation | down |
|  | *Inhba* | Inhibin beta a chain | male gonad development; ovarian follicle development | down |
|  | *Lhx9* | Lim homeobox 9 | Oocyte differentiation and survival | down |
|  | *Myl6* | Myosin light chain 6 | regulates cell growth, differentiation and migration | down |
|  | *Sox4* | Transcription factor sox-4 | positive regulation of canonical Wnt signaling pathway | up |
|  | *Ctnnd2* | Catenin delta 2 | cell adhesion and signal transduction | up |
|  | *Rora* | Retinoic acid-related orphan receptor alpha | biological clock regulation neurodevelopment; immune response and cellular stress response | up |
|  | *Hsd17b12* | Hydroxysteroid (17-beta) dehydrogenase 12 | regulation of steroid biosynthetic process | up |
|  | *Apc2* | Anaphase-promoting complex subunit 2 | megagametogenesis | up |
|  | *Fsd1* | Fibronectin type iii and spry domain containing 1 | cell adhesion migration and signaling | up |
|  | *Figla* | Factor in the germline alpha | female gonad development | up |
|  | *Ang* | Angiotensinogen | ovarian follicle rupture; response to estradiol | up |
|  | *Nsdhl* | Nad(p) dependent steroid dehydrogenase-like | 3-beta-hydroxy-delta5-steroid dehydrogenase activity; 3-beta-hydroxysteroid dehydrogenase/ 3-beta-hydroxy-delta5-steroid dehydrogenase activity; 3-beta-hydroxysteroid dehydrogenase | up |
|  | *Sdc4* | Syndecan 4 | cell adhesion migration and signaling | up |
|  | *Foxl2* | Forkhead box protein l2 | positive regulation of follicle-stimulating hormone secretion; granulosa cell differentiation; positive regulation of luteinizing hormone secretion | up |
|  | *Ror2* | Tyrosine-protein kinase transmembrane receptor ror2 | male genitalia development; female gonad development; estrogen metabolic process | up |
|  | *Tie1* | Tyrosine kinase with immunoglobulin and epidermal growth factor homology domains 1 | regulation of endothelial cell migration; proliferation and survival | up |
|  | *Sox11b* | Sry-box transcription factor 11b | sex differentiation | up |
|  | *Wee2* | Wee1-like protein kinase 2 | female meiotic nuclear division；female pronucleus assembly; negative regulation of oocyte maturation | up |
|  | *Nanog* | Nanog homeobox | self-renewal and pluripotency of embryonic stem cells | up |
|  | *Kitlg* | Kit ligand | male gonad development；ovarian follicle development | up |
|  | *Zp3* | Zona pellucida sperm-binding protein 3 | oocyte development; positive regulation of ovarian follicle development; positive regulation of antral ovarian follicle growth; negative regulation of binding of sperm to zona pellucida | up |
|  | *Foxo3* | Forkhead box protein o3 | oocyte maturation; ovulation from ovarian follicle; antral ovarian follicle growth | up |
|  | *Lbh* | Protein lbh | negative regulation of intracellular estrogen receptor signaling pathway | up |
|  | *Sox11a* | Sry-box transcription factor 11a | Neural development, tissue reconstruction and cell fate determination | up |
|  | *Rgs14* | Regulator of g protein signaling 14 | G protein signaling; cell cycle regulation | up |
|  | *Zp4* | Zona pellucida sperm-binding protein 4 | negative regulation of binding of sperm to zona pellucida | up |
|  | *Src* | Proto-oncogene tyrosine-protein kinase src | regulation of intracellular estrogen receptor signaling pathway; progesterone receptor signaling pathway | up |
|  | *Nanos3* | Nanos c2hc-type zinc finger 3 | development and maintenance of germ cells | up |
|  | *Zglp1* | Gata-type zinc finger protein 1 | spermatogenesis; oocyte development | up |
|  | *Bmp15* | Bone morphogenetic protein 15 | involved in folliculogenesis | up |
|  | *Tfap2c* | Transcription factor ap-2 gamma | embryonic development | up |
|  | *Sfrp1* | Secreted frizzled-related protein 1 | male gonad development; development of primary male sexual characteristics; female gonad development; cellular response to estradiol stimulus; negative regulation of androgen receptor signaling pathway | up |
|  | *Hsd17b12a* | Hydroxysteroid 17-beta dehydrogenase 12a | regulation of steroid biosynthetic process | up |
|  | *Sox3* | Transcription factor sox-3 | sex determination; spermatid differentiation; Sertoli cell development | up |
| 150 dpf | *Igf1* | Insulin-like growth factor 1 | regulation of cell growth cell division and metabolism | down |
|  | *Spata22* | Spermatogenesis associated 22 | meiosis in germ cells | down |
|  | *Apob* | Apolipoprotein b-100 | spermatogenesis | down |
|  | *Nr5a1* | Nuclear receptor subfamily 5  Group a | formation of the reproductive ridge | down |
|  | *Tex11* | Testis-expressed protein 11 | male gonad development | down |
|  | *Inhbb* | Inhibin beta b chain | positive regulation of ovulation; oocyte development; positive regulation of follicle stimulating hormone secretion | down |
|  | *Dmrtb1* | Doublesex and mab-3 related transcription factor b1 | male gonad development | down |
|  | *Qrich2* | Glutamine rich 2 | morphology and function of the sperm flagellum | down |
|  | *Dmc1* | Meiotic recombination protein dmc1/lim15 homolog | oogenesis; ovarian follicle development; oocyte maturation; female gamete generation | down |
|  | *Gsdf* | Gonadal soma-derived factor | sex determination and differentiation | down |
|  | *Star* | Steroidogenic acute regulatory protein | mitochondrial response to estrogen; regulation of steroid biosynthetic process; male gonad development response to steroid hormone; response to gonadotropin | down |
|  | *Acsl6* | Acyl-coa synthetase long chain family member 6 | vitellogenesis | down |
|  | *Podxl2* | Podocalyxin like 2 | cell adhesion and migration | down |
|  | *Amhr2* | Anti-mullerian hormone receptor type 2 | male sexual differentiation and the regression of female reproductive structures (Mullerian ducts) during fetal development | down |
|  | *Nanos2* | Nanos c2hc-type zinc finger 2 | development and maintenance of germ cells | down |
|  | *Cyp17a1* | Cytochrome p450 family 17 subfamily a member 1 | regulation of steroid biosynthetic process | down |
|  | *Cyp11a1* | Cytochrome p450 family 11 subfamily a member 1 | steroid hormone biosynthesis | down |
|  | *Dmrt1* | Double sex- and mab-3-related transcription factor 1 | proliferation; Sertoli cell differentiation; spermatogenesis; germ cell migration; sex differentiation; Sertoli cell development; oocyte development; developmental process involved in reproduction | down |
|  | *Gata1* | Erythroid transcription factor | male gonad development | down |
|  | *Spag6* | Sperm-associated antigen 6 | spermatogenesis; cell proliferation apoptosis | down |
|  | *Bcl2* | Bcl-2-like protein 11 | male gonad development; spermatogenesis | down |
|  | *Inha* | Inhibin alpha chain | ovarian follicle development; negative regulation of follicle-stimulating hormone secretion; male gonad development | down |
|  | *Gas2* | Growth arrest-specific protein 2 | initiation of primordial ovarian follicle growth; ovulation; antral ovarian follicle growth | down |
|  | *Rec8* | Meiotic recombination protein rec8 homolog | male meiosis I; seminiferous tubule development; spermatid development; spermatogenesis; oocyte maturation; | down |
|  | *Ar* | Androgen receptor | male sexual differentiation and development | down |
|  | *Sycp1* | Synaptonemal complex protein 1 | meiosis of germ cells; pairing and crossing over of homologous chromosomes | down |
|  | *Fshr* | Follicle-stimulating hormone receptor | primary ovarian follicle growth; ovulation cycle process; ovarian follicle development; follicle-stimulating hormone signaling pathway; spermatid development; spermatogenesis; spermatogenesis exchange of chromosomal proteins; Sertoli cell development; Sertoli cell proliferation; male gonad development | down |
|  | *Sox9* | Sry-box transcription factor 9 | male gonad development | down |
|  | *Gata4* | Gata binding protein 4 | gonadal development and maturation | down |
|  | *Vegfa* | Vascular endothelial growth factor a | ovarian follicle development | down |
|  | *Sh2b2* | Sh2b adaptor protein 2 | lipid metabolism insulin signaling | down |
|  | *Cyp17a2* | Cytochrome p450 family 17 subfamily a member 2 | regulation of steroid biosynthetic process | down |
|  | *Wnt4* | Wnt family member 4 | regulation of endothelial and steroidogenic cell migration; female gonda development | down |
|  | *Lrp2* | Low-density lipoprotein receptorrelated protein 2 | male gonad development; | down |
|  | *Amh* | Anti-mullerian hormone | development of primary male sexual characteristics | down |
|  | *Celf4* | Cugbp elav-like family member 4 | cell migration, apoptosis and stress | down |
|  | *Hsd3b1* | 3β-hydroxysteroid dehydrogenase type 1 | steroid hormone biosynthesis, ketone body metabolism | down |
|  | *Gjb1* | Gap junction beta-1 protein | epididymis development | down |
|  | *Nrip1* | Nuclear receptor-interacting protein 1 | ovarian follicle rupture; ovulation; cellular response to estradiol stimulus | down |
|  | *Pim2* | Proviral integration site 2 | cell cycle, cell survival and cell proliferation | down |
|  | *Rara* | Retinoic acid receptor alpha | response to estradiol; germ cell development; female pregnancy | down |
|  | *Folr1* | Folate receptor alpha | fusion of sperm to egg plasma membrane involved in single fertilization; sperm-egg recognition | down |
|  | *Hormad1* | Horma domain containing 1 | plays a key role in meiosis of germ cells | down |
|  | *Serinc2* | Serine incorporator 2 | cell proliferation and migration | down |
|  | *Robo2* | Roundabout homolog 2 | gonad development | down |
|  | *Sycp2* | Synaptonemal complex protein 2 | female meiotic nuclear division; male meiotic nuclear division; male genitalia morphogenesis | down |
|  | *Sycp3* | Synaptonemal complex protein 3 | meiosis of germ cells; pairing and crossing over of homologous chromosomes | down |
|  | *Penk* | Response to estradiol | response to estradiol | down |
|  | *Anxa1* | Annexin a1 | response to estradiol | down |
|  | *Sox8* | Sry-box transcription factor 8 | sex determination and differentiation | down |
|  | *Usp9x* | Probable ubiquitin carboxyl terminal hydrolase faf-x | female gamete generation; cell migration | down |
|  | *Piwil1* | P-element induced wimpy testis like protein 1 | play a key role in the development and maintenance of germ cells | down |
|  | *Fkbp4* | Peptidyl-prolyl cis-trans isomerase fkbp4 | male sex differentiation; reproductive structure development; steroid hormone receptor complex assembly; prostate gland development; androgen receptor signaling pathway | down |
|  | *Fkbp4* | High mobility group protein hmgi-c | spermatogenesis | down |
|  | *Wdr19* | Wd repeat-containing protein 19 | gonad development; in utero embryonic development | down |
|  | *Star2* | Steroidogenic acute regulatory protein | mitochondrial response to estrogen; regulation of steroid biosynthetic process; male gonad development response to steroid hormone; response to gonadotropin | down |
|  | *Wt1* | Wilms tumor 1 | gonadal development | down |
|  | *Myl6* | Myosin light chain 6 | regulates cell growth, differentiation and migration | down |
|  | *Dmrt2* | Double sex- and mab-3-related | sex differentiation | up |
|  | *Hsd17b12* | Hydroxysteroid (17-beta) dehydrogenase 12 | regulation of steroid biosynthetic process | up |
|  | *Ctnnd2* | Catenin delta 2 | cell adhesion and signal transduction | up |
|  | *Nsdhl* | Sterol-4-alpha-carboxylate 3-dehy-drogenase | decarboxylating 3-beta-hydroxy-delta5-steroid dehydrogenase activity | up |
|  | *Figla* | Factor in the germline alpha | female gonad development | up |
|  | *Cnot3* | Ccr4-not transcription complex subunit 3 | negative regulation of intracellular estrogen receptor signaling pathway | up |
|  | *Fsd1* | Fibronectin type iii and spry domain containing 1 | cell adhesion migration and signaling | up |
|  | *Zp4* | Zona pellucida sperm-binding protein 4 | negative regulation of binding of sperm to zona pellucida | up |
|  | *Tie1* | Tyrosine kinase with immunoglobulin and epidermal growth factor homology domains 1 | regulation of endothelial cell migration, proliferation and survival | up |
|  | *Zp3* | Zona pellucida sperm-binding protein 3 | oocyte development; positive regulation of ovarian follicle development; positive regulation of antral ovarian follicle growth; negative regulation of binding of sperm to zona pellucida | up |
|  | *Rgs14* | Regulator of g protein signaling 14 | G protein signaling; cell cycle regulation | up |
|  | *Sdc4* | Syndecan 4 | cell adhesion migration | up |
|  | *Foxo3* | Forkhead box protein o3 | oocyte maturation; ovulation from ovarian follicle; antral ovarian follicle growth | up |
|  | *Kitlg* | Kit ligand | male gonad development; ovarian follicle development | up |
|  | *Sox11b* | Sry-box transcription factor 11b | sex differentiation | up |
|  | *Apc2* | Anaphase-promoting complex subunit 2 | megagametogenesis | up |
|  | *Src* | Proto-oncogene tyrosine-protein kinase src | regulation of intracellular estrogen receptor signaling pathway; progesterone receptor signaling pathway | up |
|  | *Ror2* | Tyrosine-protein kinase transmembrane receptor ror2 | male genitalia development; female gonad development; estrogen metabolic process | up |
|  | *Bmp15* | Bone morphogenetic protein 15 | involved in folliculogenesis | up |
|  | *Lbh* | Protein lbh | negative regulation of intracellular estrogen receptor signaling pathway | up |
|  | *Tfap2c* | Transcription factor ap-2 gamma | embryonic development, cell proliferation and differentiation | up |
|  | *Foxl2* | Forkhead box protein l2 | positive regulation of follicle-stimulating hormone secretion; granulosa cell differentiation; positive regulation of luteinizing hormone secretion | up |
|  | *Sox11a* | Sry-box transcription factor 11a | Neural development; tissue reconstruction | up |
|  | *Sfrp1* | Secreted frizzled-related protein 1 | male gonad development; development of primary male sexual characteristics; female gonad development; cellular response to estradiol stimulus; negative regulation of androgen receptor signaling pathway | up |
|  | *Zglp1* | Gata-type zinc finger protein 1 | spermatogenesis; oocyte development | up |
|  | *Hsd17b12a* | Hydroxysteroid 17-beta dehydrogenase 12a | regulation of steroid biosynthetic process | up |
|  | *Sox3* | Transcription factor sox-3 | sex determination; spermatid differentiation; Sertoli cell development | up |
| 180 dpf | *Qrich2* | Glutamine rich 2 | morphology and function of the sperm flagellum | down |
|  | *Ar* | Androgen receptor | male sexual differentiation and development | down |
|  | *Apob* | Apolipoprotein b-100 | spermatogenesis | down |
|  | *Cyp17a1* | Cytochrome p450 family 17 subfamily a member 1 | regulation of steroid biosynthetic process | down |
|  | *Star* | Steroidogenic acute regulatory protein | mitochondrial response to estrogen; regulation of steroid biosynthetic process; male gonad development response to steroid hormone; response to gonadotropin | down |
|  | *Nr5a1* | Nuclear receptor subfamily 5 group a | formation of the reproductive ridge | down |
|  | *Lhx8* | Lim homeobox 8 | Oocyte differentiation and survival | down |
|  | *Cyp17a2* | Cytochrome p450 family 17 subfamily a member 2 | regulation of steroid biosynthetic process | down |
|  | *Cyp11a1* | Cytochrome p450 family 11 subfamily a member 1 | steroid hormone biosynthesis | down |
|  | *Lrp2* | Low-density lipoprotein receptor related protein 2 | male gonad development; | down |
|  | *Gsdf* | Gonadal soma-derived factor | sex determination and differentiation | down |
|  | *Dmrt1* | Double sex- and mab-3-related transcription factor 1 | proliferation; Sertoli cell differentiation; spermatogenesis; germ cell migration; sex differentiation; Sertoli cell development; oocyte development; developmental process involved in reproduction | down |
|  | *Folr1* | Folate receptor alpha | fusion of sperm to egg plasma membrane involved in single fertilization; sperm-egg recognition | down |
|  | *Spata22* | Spermatogenesis associated 22 | meiosis in germ cells | down |
|  | *Hsd3b1* | 3β-hydroxysteroid dehydrogenase type 1 | steroid hormone biosynthesis; ketone body metabolism | down |
|  | *Inhbb* | Inhibin beta b chain | positive regulation of ovulation; oocyte development; positive regulation of follicle stimulating hormone secretion | down |
|  | *Bcl2* | Bcl-2-like protein 11 | male gonad development; spermatogenesis | down |
|  | *Inhbb* | Inhibin beta b chain | positive regulation of ovulation; oocyte development; positive regulation of follicle stimulating hormone secretion | down |
|  | *Inha* | Inhibin alpha chain | ovarian follicle development; negative regulation of follicle-stimulating hormone secretion; male gonad development | down |
|  | *Fshr* | Follicle-stimulating hormone receptor | primary ovarian follicle growth; ovulation cycle process; ovarian follicle development; follicle-stimulating hormone signaling pathway; spermatid development; spermatogenesis; spermatogenesis exchange of chromosomal proteins; Sertoli cell development; Sertoli cell proliferation; male gonad development | down |
|  | *Gata4* | Gata binding protein 4 | gonadal development and maturation | down |
|  | *Sox9* | Sry-box transcription factor 9 | male gonad development | down |
|  | *Vegfa* | Vascular endothelial growth factor a | ovarian follicle development | down |
|  | *Gas2* | Growth arrest-specific protein 2 | initiation of primordial ovarian follicle growth; ovulation; antral ovarian follicle growth | down |
|  | *Sycp2* | Synaptonemal complex protein 2 | female meiotic nuclear division; male meiotic nuclear division; male genitalia morphogenesis | down |
|  | *Amhr2* | Anti-mullerian hormone receptor type 2 | male sexual differentiation and the regression of female reproductive structures (Mullerian ducts) during fetal development | down |
|  | *Sycp1* | Synaptonemal complex protein 1 | meiosis of germ cells; pairing and crossing over of homologous chromosomes | down |
|  | *Amh* | Anti-mullerian hormone | development of primary male sexual characteristics | down |
|  | *Celf4* | Cugbp elav-like family member 4 | cell migration, apoptosis and stress | down |
|  | *Frem2* | Fras1 related extracellular matrix protein 2 | cell adhesion and migration | down |
|  | *Bmp7* | Bone morphogenetic protein 7 | response to estradiol | down |
|  | *Tet1* | Tet methylcytosine dioxygenase 1 | DNA methylation and demethylation | down |
|  | *Smad6* | Mothers against decapentaplegic homolog 6 | response to estrogen | down |
|  | *Spag6* | Sperm-associated antigen 6 | spermatogenesis; cell proliferation apoptosis | down |
|  | *Acsl6* | Acyl-coa synthetase long chain family member 6 | vitellogenesis | down |
|  | *Myh11* | Myosin heavy chain 11 | smooth muscle cell function, cell migration and proliferation | down |
|  | *Ash1l* | Histone-lysine n-methyltransferase ash1l | flagellated sperm motility; uterine gland development | down |
|  | *Col1a2* | Collagen type i alpha 2 chain | cell proliferation and migration | down |
|  | *Gata1* | Erythroid transcription factor | male gonad development | down |
|  | *Foxo3* | Forkhead box protein o3 | oocyte maturation; ovulation from ovarian follicle; antral ovarian follicle growth | down |
|  | *Zmiz1* | Zinc finger miz domain-containing protein 1 | vitellogenesis | down |
|  | *Ctnnd2a* | Catenin delta 2a | cell adhesion and signal transduction | down |
|  | *Zmiz1* | Zinc finger miz domain-containing protein 1 | female meiosis chromosome segregation; male meiosis chromosome segregation | down |
|  | *Wt1* | Wilms tumor 1 | gonadal development | down |
|  | *Penk* | Response to estradiol | response to estradiol | down |
|  | *Wnt4* | Wnt family member 4 | regulation of endothelial and steroidogenic cell migration; female gonda development | down |
|  | *Etv4* | Ets variant transcription factor 4 | Proliferation and differentiation of embryonic stem cells | down |
|  | *Nrip1* | Nuclear receptor-interacting protein 1 | ovarian follicle rupture; ovulation; cellular response to estradiol stimulus | down |
|  | *Gata3* | Gata binding protein 3 | cell development and differentiation | down |
|  | *Podxl2* | Podocalyxin like 2 | cell adhesion and migration | down |
|  | *Sox17* | Sry-box transcription factor 17 | embryonic development and blood vessel formation | down |
|  | *Pdgfa* | Platelet-derived growth factor receptor alpha | male genitalia development | down |
|  | *Bcl6b* | B-cell lymphoma 6 protein | spermatogenesis | down |
|  | *Id4* | Dna-binding protein inhibitor id-4 | seminal vesicle morphogenesis | down |
|  | *Tgfb1* | Transforming growth factor beta-1 proprotein | response to estradiol; response to progesterone | down |
|  | *Vcam1* | Vascular cell adhesion molecule 1 | migration differentiation and proliferation of gonadal cells | down |
|  | *Ptch1* | Protein patched homolog 1 | response to estradiol | down |
|  | *Wdr19* | Wd repeat-containing protein 19 | gonad development; in utero embryonic development | down |
|  | *Mmp14* | Matrix metalloproteinase-14 | response to estrogen; ovarian follicle development | down |
|  | *Kit* | Mast/stem cell growth factor receptor kit | ovarian follicle development; germ cell development; germ cell migration; male gonad development; spermatid development; spermatogenesis | down |
|  | *Taf4* | Transcription initiation factor tfiid subunit 4 | ovarian follicle development | down |
|  | *Dock8* | Dedicator of cytokinesis 8 | cell proliferation and migration | down |
|  | *Fgfr3* | Fibroblast growth factor receptor 3 | cell growth, differentiation and development | down |
|  | *Robo2* | Roundabout homolog 2 | gonad development | down |
|  | *Lhx9* | Lim homeobox 9 | Oocyte differentiation and survival | down |
|  | *Usp9x* | Probable ubiquitin carboxyl terminal hydrolase faf-x | female gamete generation; cell migration | down |
|  | *Dlg1* | Disks large 1 tumor suppressor protein | ovarian follicle cell development | down |
|  | *Prdm1* | Pr domain zinc finger protein 1) | cell differentiation and immune response | down |
|  | *Emx2* | Empty spiracles homeobox 2 | formation of the reproductive ridge | down |
|  | *Pim2* | Proviral integration site 2 | cell cycle, cell survival and cell proliferation | down |
|  | *Txnip* | Thioredoxin-interacting protein | response to estradiol; response to progesterone | down |
|  | *Myl6* | Myosin light chain 6 | regulates cell growth, differentiation and migration | down |
|  | *Anxa1* | Annexin a1 | response to estradiol | down |
|  | *Serinc2* | Serine incorporator 2 | cell proliferation and migration | down |
|  | *Ube3a* | Ubiquitin-protein ligase e3a | ovarian follicle development; sperm entry; response to progesterone; androgen receptor signaling pathway | down |
|  | *Sox4* | Transcription factor sox-4 | positive regulation of canonical Wnt signaling pathway | down |
|  | *Dvl1* | Dishevelled segment polarity protein 1 | cell fate determination, proliferation and migration | down |
|  | *Wnt2b* | Protein wnt-2b | male gonad development | down |
|  | *Hsd17b12* | Hydroxysteroid (17-beta) dehydrogenase 12 | regulation of steroid biosynthetic process | up |
|  | *Sox8* | Sry-box transcription factor 8 | sex determination and differentiation | up |
|  | *Dedd* | Death effector domain-containing protein | spermatogenesis | up |
|  | *Npm1* | Nucleophosmin 1 | regulation of cell growth and division | up |
|  | *Src* | Proto-oncogene tyrosine-protein kinase src | regulation of intracellular estrogen receptor signaling pathway; progesterone receptor signaling pathway | up |
|  | *Ace* | Angiotensin-converting enzyme | spermatogenesis | up |
|  | *Vegfa* | Vascular endothelial growth factor a | ovarian follicle development | up |
|  | *Wee1b* | Wee1-like protein kinase 1-b | female meiotic nuclear division; female pronucleus assembly; negative regulation of oocyte maturation | up |
|  | *Rora* | Retinoic acid-related orphan receptor alpha | biological clock regulation neurodevelopment; immune response and cellular stress response | up |
|  | *Ddx4* | Dead-box helicase 4 | survival and maintenance of germ cells | up |
|  | *Wnt9b* | Protein wnt-9b | male genitalia development | up |
|  | *Foxl2* | Forkhead box protein l2 | positive regulation of follicle-stimulating hormone secretion; granulosa cell differentiation; positive regulation of luteinizing hormone secretion | up |
|  | *Tie1* | Tyrosine kinase with immunoglobulin and epidermal growth factor homology domains 1 | regulation of endothelial cell migration, proliferation and survival | up |
|  | *Sox11b* | Sry-box transcription factor 11b | sex differentiation | up |
|  | *Ror2* | Tyrosine-protein kinase transmembrane receptor ror2 | male genitalia development; female gonad development; estrogen metabolic process | up |
|  | *Lbh* | Protein lbh | negative regulation of intracellular estrogen receptor signaling pathway | up |
|  | *Apc2* | Anaphase-promoting complex subunit 2 | megagametogenesis | up |
|  | *Spo11* | Meiotic recombination protein spo11 | Oogenesis; ovarian follicle development; spermatid development | up |
|  | *Dnd1* | Dead end homolog 1 | survival and maintenance of germ cells | up |
|  | *Foxo3* | Forkhead box protein o3 | oocyte maturation; ovulation from ovarian follicle; antral ovarian follicle growth | up |
|  | *Wee2* | Wee1-like protein kinase 2 | female meiotic nuclear division; female pronucleus assembly; negative regulation of oocyte maturation | up |
|  | *Ctnnd2* | Catenin delta 2 | cell adhesion and signal transduction | up |
|  | *Sall4* | Spalt-like transcription factor 4 | embryonic development and stem cell pluripotency | up |
|  | *Cpeb1* | Cytoplasmic polyadenylation element binding protein 1 | Oocyte maturation and early embryonic development | up |
|  | *Sox11a* | Sry-box transcription factor 11a | neural development  tissue reconstruction and cell fate determination | up |
|  | *Rgs14* | Regulator of g protein signaling 14 | G protein signaling; cell cycle regulation | up |
|  | *Nsdhl* | Sterol-4-alpha-carboxylate 3-dehy-drogenase | 3-beta-hydroxy-delta5-steroid dehydrogenase activity; 3-beta-hydroxysteroid dehydrogenase/ 3-beta-hydroxy-delta5-steroid dehydrogenase activity; 3-beta-hydroxysteroid dehydrogenase | up |
|  | *Nanos3* | Nanos c2hc-type zinc finger 3 | development and maintenance of germ cells | up |
|  | *Figla* | Factor in the germline alpha | female gonad development | up |
|  | *Hsd17b12a* | Hydroxysteroid 17-beta dehydrogenase 12a | regulation of steroid biosynthetic process | up |
|  | *Sfrp1* | Secreted frizzled-related protein 1 | male gonad development; development of primary male sexual characteristics; female gonad development; cellular response to estradiol stimulus; negative regulation of androgen receptor signaling pathway | up |
|  | *Sox3* | Transcription factor sox-3 | sex determination; spermatid differentiation; Sertoli cell development | up |
|  | *Nanog* | Nanog homeobox | self-renewal and pluripotency of embryonic stem cells | up |
|  | *Tfap2c* | Transcription factor ap-2 gamma | embryonic development; cell proliferation and differentiation | up |
|  | *Bmp15* | Bone morphogenetic protein 15 | involved in folliculogenesis | up |
|  | *Zglp1* | Gata-type zinc finger protein 1 | spermatogenesis; oocyte development | up |
|  | *Zp4* | Zona pellucida sperm-binding protein 4 | negative regulation of binding of sperm to zona pellucida | up |
|  | *Zp3* | Zona pellucida sperm-binding protein 3 | oocyte development; positive regulation of ovarian follicle development; positive regulation of antral ovarian follicle growth; negative regulation of binding of sperm to zona pellucida | up |

**Table S7** List of GO terms enriched from 165 sex-related DEGs.

| **GO ID** | **GO term** | **-Log(p-value)** | **Gene number** |
| --- | --- | --- | --- |
| GO:0008585 | female gonad development | 4.732828 | 32 |
| GO:0046545 | development of primary female sexual characteristics | 4.672355 | 38 |
| GO:0046660 | female sex differentiation | 4.493495 | 28 |
| GO:0008406 | gonad development | 4.321482 | 22 |
| GO:0007548 | sex differentiation | 3.940569 | 20 |
| GO:0048608 | reproductive structure development | 3.690557 | 21 |
| GO:0019953 | sexual reproduction | 3.656292 | 18 |
| GO:0007292 | female gamete generation | 1.972061 | 15 |
| GO:0046546 | development of primary male sexual characteristics | 1.408018 | 15 |
| GO:0008584 | male gonad development | 1.408018 | 16 |
| GO:0046661 | male sex differentiation | 1.11547 | 14 |
| GO:0045137 | development of primary sexual characteristics | 4.321482 | 18 |
| GO:0005615 | extracellular space | 5.774691 | 22 |
| GO:0045495 | pole plasm | 5.571865 | 21 |
| GO:0060293 | germ plasm | 5.571865 | 23 |
| GO:0035770 | ribonucleoprotein granule | 4.345823 | 20 |
| GO:0036464 | cytoplasmic ribonucleoprotein granule | 4.345823 | 22 |
| GO:0008083 | growth factor activity | 7.308035 | 22 |
| GO:0003700 | transcription factor activity, sequence-specific DNA binding | 6.123782 | 16 |
| GO:0001071 | nucleic acid binding transcription factor activity | 6.112383 | 19 |
| GO:0005102 | receptor binding | 5.614394 | 10 |
| GO:0016229 | steroid dehydrogenase activity | 4.294136 | 15 |

**Table S8** List of KEGG pathways enriched from 165 sex-related DEGs.

| **Ko ID** | **Ko term** | **-Log(p-value)** | **Gene number** | |  |
| --- | --- | --- | --- | --- | --- |
| ko04512 | ECM-receptor interaction | 7.839654 | 20 | |  |
| ko04110 | Cell cycle | 5.772848 | 15 | |  |
| ko04114 | Oocyte meiosis | 4.848688 | 9 | |  |
| ko04912 | GnRH signaling pathway | 4.297775 | 7 | |  |
| ko00100 | Steroid biosynthesis | 4.22909 | 6 | |  |
| ko00140 | Steroid hormone biosynthesis | 4.119355 | 6 | |  |
| ko04979 | Cholesterol metabolism | 4.119355 | 4 | |  |
| ko04340 | Hedgehog signaling pathway | 3.859315 | | 4 | |
| ko04060 | Cytokine-cytokine receptor interaction | 3.523056 | | 5 | |
| ko04068 | FoxO signaling pathway | 3.518137 | | 4 | |
| ko04310 | Wnt signaling pathway | 3.405418 | | 7 | |
| ko04350 | TGF-beta signaling pathway | 3.323117 | | 6 | |
| ko04910 | Insulin signaling pathway | 2.375607 | | 3 | |
| ko04913 | Ovarian steroidogenesis | 2.356603 | | 4 | |
| ko04010 | MAPK signaling pathway | 2.297326 | | 3 | |

**Table S9** List of GO terms enriched from the female profile DEGs.

| **Go ID** | **Go term** | **-Log(p-value)** | **Gene number** |
| --- | --- | --- | --- |
| GO:0032502 | developmental process | 6.954325 | 870 |
| GO:0007049 | cell cycle | 5.910738 | 568 |
| GO:1903046 | meiotic cell cycle process | 5.192561 | 453 |
| GO:0019953 | sexual reproduction | 7.590302 | 585 |
| GO:0007276 | gamete generation | 2.785771 | 441 |
| GO:0007143 | female meiotic nuclear division | 4.531332 | 353 |
| GO:0007292 | female gamete generation | 4.7078 | 408 |
| GO:0008585 | female gonad development | 4.23682 | 224 |
| GO:0046545 | development of primary female sexual characteristics | 3.9105913 | 265 |
| GO:0000278 | mitotic cell cycle | 5.212617 | 501 |
| GO:0042995 | cell projection | 19.8472 | 1091 |
| GO:0120025 | plasma membrane bounded cell projection | 19.64057 | 1225 |
| GO:0005856 | cytoskeleton | 19.64057 | 935 |
| GO:0005815 | microtubule organizing center | 10.24519 | 622 |
| GO:0043005 | neuron projection | 9.836311 | 552 |
| GO:0003674 | molecular function | 13.87293 | 893 |
| GO:0005515 | protein binding | 11.31692 | 576 |
| GO:0008092 | cytoskeletal protein binding | 6.496294 | 486 |
| GO:0005524 | ATP binding | 5.481692 | 541 |
| GO:0008289 | lipid binding | 5.413591 | 255 |

**Table S10** List of KEGG pathways enriched from the female profile DEGs.

| **Ko ID** | **Ko term** | **-Log(p-value)** | **Gene number** |
| --- | --- | --- | --- |
| ko00140 | Steroid hormone biosynthesis | 20.30518 | 148 |
| ko04114 | Oocyte meiosis | 19.12835 | 115 |
| ko04512 | ECM-receptor interaction | 17.96772 | 159 |
| ko04914 | Progesterone-mediated oocyte maturation | 14.38517 | 76 |
| ko04068 | FoxO signaling pathway | 6.1324 | 116 |
| ko04910 | Insulin signaling pathway | 5.990154 | 108 |
| ko04913 | Ovarian steroidogenesis | 3.427251 | 41 |
| ko04310 | Wnt signaling pathway | 4.000108 | 137 |
| ko04912 | GnRH signaling pathway | 4.310514 | 71 |
| ko04350 | TGF-beta signaling pathway | 5.777888 | 65 |

**Table S11** List of GO terms enriched from the male profile DEGs.

| **GO ID** | **GO term** | **-Log(p-value)** | **Gene number** |
| --- | --- | --- | --- |
| GO:0007140 | male meiotic nuclear division | 13.87367 | 263 |
| GO:0048232 | male gamete generation | 16.9333 | 275 |
| GO:0007060 | male meiosis chromosome segregation | 11.84635 | 302 |
| GO:0007141 | male meiosis I | 18.84635 | 243 |
| GO:2000018 | regulation of male gonad development | 12.94877 | 395 |
| GO:0060179 | male mating behavior | 9.385882 | 621 |
| GO:2000254 | regulation of male germ cell proliferation | 9.385882 | 103 |
| GO:0046661 | male sex differentiation | 9.385882 | 229 |
| GO:0030238 | male sex determination | 5.841872 | 135 |
| GO:0008584 | male gonad development | 5.841872 | 101 |
| GO:0005622 | intracellular | 69.38116 | 1236 |
| GO:0044424 | obsolete intracellular part | 69.20221 | 1399 |
| GO:0043229 | intracellular organelle | 53.41728 | 1022 |
| GO:0043227 | membrane-bounded organelle | 42.12946 | 1212 |
| GO:0099081 | supramolecular polymer | 8.875335 | 232 |
| GO:0003674 | molecular function | 41.40148 | 1893 |
| GO:0003824 | catalytic activity | 25.80449 | 861 |
| GO:0005515 | protein binding | 23.06294 | 433 |
| GO:0016740 | transferase activity | 8.365812 | 148 |
| GO:0008092 | cytoskeletal protein binding | 4.953216 | 301 |

**Table S12** List of KEGG pathways enriched from the male profile DEGs.

| **Ko ID** | **Ko term** | **-Log(p-value)** | **Gene number** |
| --- | --- | --- | --- |
| ko04512 | ECM-receptor interaction | 17.87689 | 125 |
| ko04912 | GnRH signaling pathway | 6.724309 | 112 |
| ko04010 | MAPK signaling pathway | 7.513167 | 126 |
| ko04350 | TGF-beta signaling pathway | 5.501732 | 127 |
| ko04013 | MAPK signaling pathway - fly | 6.234183 | 131 |
| ko04110 | Cell cycle | 16.10361 | 125 |
| ko03010 | Ribosome | 28.71639 | 241 |
| ko04510 | Focal adhesion | 18.40408 | 162 |
| ko03320 | PPAR signaling pathway | 18.40408 | 144 |
| ko00982 | Drug metabolism - cytochrome P450 | 16.82333 | 198 |
